# Enhanced postoperative surveillance versus standard of care to reduce mortality among adult surgical patients in Africa (ASOS-2): a cluster-randomised controlled trial

**DOI:** 10.1016/S2214-109X(21)00291-6

**Published:** 2021-08-19

**Authors:** The ASOS-2 Investigators, Bruce M Biccard, Leon du Toit, Maia Lesosky, Tim Stephens, Landon Myer, Agya BA Prempeh, Nicola Vickery, Hyla-Louise Kluyts, Alexandra Torborg, Akinyinka Omigbodun, Adesoji Ademuyiwa, Muhammed Elhadi, Mohamed Elfagieh, Bernard Mbwele, Mpoki Ulisubisya, Lazaro Mboma, Daniel Z Ashebir, Mahlet Tesfaye Bahta, Mohammed Hassen, Mikiyas Teferi, Yakob Seman, Eugene Zoumenou, Adam Hewitt-Smith, Janat Tumukunde, Dolly Munlemvo, Atilio Morais, Apollo Basenero, Pisirai Ndarukwa, Nazinigouba Ouerdraogo, Maman Sani Chaibou, Mohyeddine Zarouf, Ahmed Rhassane El Adib, Veekash Gobin, Zimogo Sanogo, Youssouf Coulibaly, Zipporah Ngumi, Tarig Fadalla, Cynthia Iradukunda, Vénérand Barendegere, Isaac O Smalle, Mustapha Bittaye, Ahmadou Lamin Samateh, Mahmoud Elfiky, Maher Fawzy, Wakisa Mulwafu, Vanessa Msosa, Lygia Lopes, Akwasi Antwi-Kusi, Hamza D Sama, Patrice Forget, Dawid van Straaten, Rupert M Pearse, Marichen Puchert, Lucy Rolt, Kris Schwebler, Freddy Kabambi, Tebogo Mabotja, Leandys Cobas, Albino Freitas, Maria Antunes, Bartolomeu Cabo, Domingos Paulo, Carlos Camongua, Yvette Avognon, Osseni Marcos, Raymond Kintomonho, Onesime Demahou, Gisèle Hounsa, Hugues Chobli, Elie Fassinou, Aurore Zoglobossou, Blaise Tchaou, Charles Tchegnonsi, Fifame Amadji, Francine Bossa, Ernest Ahounou, Djima Alao, Roushdane Odérémi, Afissatou Montairou, Oswald Gbehade, Romaric Tobome, Adam Boukari, Patrick Bakantieba, Arouna Sambo, Fanou Lionelle, Nounagnon Gilbert, Julien Attinon, Roger Klikpezo, Aumar Dadjo, Dénis Fanou, Gilberte Hounkpe, Bachabi Fafana, Néné Nguilu, Bodourin Dossou-Yovo, Chantal Segla, Mohamed Toko, Evelyne Gnele-Dedewanou, Michel Noukounwoui, Ethienne Yado, Timothé Gouroubéra, Valéry Adjignon, Serge Mewanou, Aïcha Tchomgang, Urielle Agossou, Fernand Soton, Charbel Azanlin, Lidwine Zomahoun, Rawéléguinbasba Armel Flavien Kabore, Salam Savadogo, Fatou Fleur Rosine Sanou, Farid Belém, Victoria Hien, Cheik Tidiane Hafid W Bougouma, Sie Ahmed Ouattara, Mariam Bambara Kabore, Ouedraogo Nazinigouba, Papougnezambo Bonkoungou, Martin Lankoandé, Mireille Traoré, Patrick Sawadogo, Inès Wenmenga, Boureima Kinda, André Simporé, Christian Sapo, Salah Idriss Traore, Haoua Dipama, Lydie WR Kaboré, Salifou Napon, Télesphore G Kaboré, Arouna Louré, Pélagie PP Tondé, Christian Zoundi, Harouna Sanou, Remy Ndikumana, Carlos Nsengiyumva, Gregory Sund, Alliance Niyukuri, Axel Kwizera, Jean-Claude Niyondiko, Adolphe Manzanza Kilembe, Jean Pierre Mwema Ilunga, Nehema Hailemariam Sarah, Gabriel Mubobo Makeya, Idesbald Mwebe Mwepu, Ted Botawaosenge Likongo, Richard Kapela Mvwala, Raphael Nzau Kapend Mubunda, Noellie Kanka Mukuna, Julie Djondo Pembe, Nicolas Lumuanga Ndaye, Eric Bibonge Amisi, Mike Ilunga Madika, Joe Kembo Lungela, Didier Ndonda Mayemba, Philomene Mamba Diyoyo, Alex Mbo Ngalala, Martin Mamba Mukenga, Patricia Tito Kabuni, Dany Bolimo Mpoto, Herve Inesnku Mole, Louise Keby, Oria Andavo Buti, Anselme Phaka, Belinda Mayenge, Jean Jeacques Kabuley Kalongo, Timothe Kemfuni Mawisa, Rodrigue Tondo Ngwizani, Kuyala Leya, Dieudonne Kisile Sanduku, Timothe Nkemfuni Mawisa, Coco Nseke Mfumu, Mbuta Bolenge, Desire Kinzenzengu Kabuce, Patrick Kintieti, Amelia Mbuluku, Vicky Mahuwa, Tharcice Khonde Mabiala, Guilain Ngoy, Patrick Boloko, Nono Mazangama Mvwama, Jose Kengbanda, Pitchou Mushimbonga, Blaise Kuhapala, Nzosani Marcel, Kienze Guylain, Gerard Mboma, Sandra Zalambo Sagboze, Michel Muteya Manika, Jean Pierre Mumbere Kigayi, Roger Mukanire Cishugi, Placide Buhendwa Mugisho, Roger Baguma, Moïse Kongolo, Michel Mandungu Mbayabu, Crispin Mukendi Muamba, Edmond Banema Kapinga, Vasco Ngolela Kapinga, Guylain Tshimanga Nsumpi, Patrick Kanda Odia, Salomon Bingidimi, Gilbert Kpengbemale, Desire Hubert Bofunga Bosonga Imposo, Patricia Matondo, Servet Lelo, Jeremie Kalambayi, Patrick Kanda Odia, Mohamed Abdel-Ghaffar, Abdelrahman Soliman, Mostafa Abdelrahman, Sameh Shehata, Alia Rabee, Mohamed Abou Heba, Mohamed Rabei Abdelfattah, Tamer Ahmed Maher Ghoniem, Sherif M.K. Shehata, Mohamed Lotfy, Ahmed ElHaddad, Bereket Gebremeskel, Girmay Fisseha, Mebrahtu Abay, Degena Bahrey, Assefa Hika, Abdurezak Ali, Kindie Moges, Jemal Ahmed, Desalegn Abdisa, Abebe Megersa, Wendwosen Abayne, Haftom Berhane, Reiye Esayas, Fitsum Kifle, Kokeb Desita, Abebe Addise, Dagim Shimelash, Shitalem Tadesse, Bezaye Zemedkun, Peniel Kenna, Ayenew Yirdie, Abinet Sisay, Tebikew Gashu, Fassil Mihretu, Yesuf Ahmed, Bekele Debebe, Abdureuf Misgea, Amare Agmas, Rahel Assefa, Abdurahman Gelmo, Seifu Alemu, Brook Damtachew, Merid Mersha, Yaekob Chemere, Samuel Fekadu, Sintayehu Regasa, Bonsa Sileshi, Desalegn Wosen, Rebira Adamu, Gersam Mulugeta, Teshome Bacha, Zewude Gudisa, Kebebe Bekele, Alelign Tasew, Habtamu Gezahegn, Daniel Atlaw, Damtew Solomon, Habtemariam Gebresillasie, Girmaye Tesfaye, Negussie Sarbecha, Biniyam Sahiledengle, Kenbon Seyoum, Gemechu Ganfure, Yohannes Tekalegn, Gosa Tesfaye, Temesgen Ayichew, Shibiru Sendaba, Musefa Redwan, Eyasu Muse, Girma Nina, Bizuneh Sime, Addisalem Tadege, Anbesse Jima, Nugusu Ayalew, Dagmawi Workneh, Daniel Teferi, Momodou T Nyassi, Landing N Sanyang, Omar Jallow, Abdoulie Keita, Kitabu Jammeh, Charles Roberts, Patrick Idoko, Kebba Marenah, Masirending Njie, Musa Marena, Karamba Suwareh, Simon Boissey, Fatoumatta Jarjusey, Awa Jah, Awa Sanyang, Dado Jabbi, Kajali Camara, Armando Correa, John Jabang, Lamin Jaiteh, Lamin Dampha, Aminata Manneh, Abdoulie Keita, Baboucarr Sowe, Abdoulie Bah, Edrisa Jawo, Victoria Okoje, Momodou Baro, Yaya Bah, Mustapha Njie, Sainabou Mbowe, Ebrima Kanteh, Sarjo Ceesay, Alagie Manneh, Evans Atito-Narh, Adwoa Wilson, Romeo Hussey, Emmanuel Okine, Jemima Kwarteng, Ridge Ntiamoah, Samuel Dadzie, Mark Aseti, Naa Sowah, Akosua Appiah, Charles Bankah, Patrick Mburugu, Thomas Chokwe, Patrick Olang, Vernon Gacii, Susane Nabulindo, Antony Gatheru, Timothy Mwiti, Caroline Mwangi, Julius Muriithi, Daniel Ojuka, Omondi Ogutu, Evans Masitara, Mohamed Chaudhry, Reuben Kamundi, Annmarie Kangangi, Thomas Massaquoi, Stephen Takow, Felister Moraa, Aziz Munubi, Moses Kimani, Adili Wobenjo, Linda Nguu, Vincent Omeddo, Rose Malaba, Ambrose Nabwana, Anita Mwancha, Alexandria Mugaa, John Wamwaki, Joyce Chege, Seymour Sinari, Andrew Ndonga, Rose Shitsinzi, Walter Akello, Winfred Kimani, Elisha Kirwa, Seno Saruni, Andrew Wainaina, Ernest Nshom, Aidah Kenseko, Kizito Shisanya, Purity Wanjiru, Julliah Cherotich, Judy Kimutai, Benson Wahome, Grace Wangui, Dennis Wamalwa, Stephen Mwangi, John Chege, Tabitha Wanjiku, Carolyne Njoki, Wachira Waititu, Conrad Ambani, Samuel Murimi, Sharon Waithira, Nilson Mouti, Collins Kibet, John Kibet, Virginia Sokobe, Beatrice Jeymah, Antony Kamadi, Faith Gichuri, Steve Moses, David Wasike, Favours Adeya, Caesar Bitta, Stephen Ogendo, Killian Kariuki, Hdaya BenAbdalla, Taha Suliaman, Fatima Ali Abokhzam, Mohaned Isa, Mohammed Huwaysh, Asma Bourawi, Kais Alzubaidy, Mohammed Albaraesi, Sumayyah Bahroun, Abdulmueti Alhadi, Ahmed Msherghi, Amira Mohamed, Ala Khaled, Nouran Aljadi, Elham Bareig, Khaled Elgazwi, Adel Elgazwi, Ibrahim El-busife, Safa Owhida, Almahdi Eltwati, Samah Elakeili, Taha Abubaker, Fatima Elkhfeefi, Soha Younis Hasan, Amal Alttaira, Imbarkah Elmraied, Rim Wishah, Omar Abugassa, Hazem Ahmed, Amera Ellafi, Shoukrie Shoukrie, Nawal Aldokali, Aws ElGammudi, Akram Alkaseek, Hoda Elhaddad, Ayyah Alqaarh, Ahlam Brish, Malek Abudsnnuga, Salsabil Albuaishi, Mohammed Albashri, Marwa Morgom, Mohammed Alawami, Eman Shawesh, Abdullah Almabrouk, Moaz Alwarfalli, Nagia Abeid, Anis Buzreg, Ans Malek, Ameerah Abayu, Butaina Abdulhafith, Wedad Abouruwes, Marim Albakuri, Sabria Almuammari, Esam Alsaghair, Fatheia Alreshi, Hassan Badi, Rabiee Alfetoey, Naji Zubia, Bushray Almiqlash, Abdulsalam Alshuhoumi, Rayet Al-islam Ben Jouira, Amaal Dier, Essra Gebril, Mohammed Abdelkabir, Sana Moussa, Marwa Alfitori, Mabroukah Azbeda, Hajar Alamin, Ejmeya Barka, Omlsaad Mosbah, Rema Abdasalam, Miftah Hiyoum, Maryam Abd allateef, Ibrahim Altomi, Mahdi Alsakloul, Ekhlas Karami, Munyah Alriqeeq, Mabruka Omar, Ashraf Samer, Aml Aemeesh, Ahmad Bouhuwaish, Ahmed Msherghi, Ahmed Elusta, Sultan Ahmeed, Wesal AlFighi Hassan, Mouadah Ali Altayr, Mohamed Addalla, Abrar Geddeda, Kheria Khoja, Doaa Alhaj, Muaad Etturki, Ahmed Elhadi, Ibrahim Ellojli, Anshirah Shuwayyah, Ahmed Elfaghih, Malak Alduwayb, Mohamed Aleiyan, Wedad Aboubreeq, Soliman Alkassem, Sami Ashour, Hayat Ben Hasan, Najat Ben Hasan, Ali Yahya, Palesa Chisala, Edward Kommwa, Lusayo Simwinga, Agness Chalira, Precious Kachitsa, Onias Mtalimanja, Drissa Traoré, Moussa Sissoko, Moussa Camara, Adama Koita, Sekou Koumaré, Omar Sacko, Mahamadou Coulibaly, Lamine Soumaré, Soumaïla Keita, Sidiki Keita, Hamadoun Dicko, Boubacar Diallo, Boureima Bengaly, Mohamed Keita, Siaka Diallo, Drissa Ouattara, Nouhoun Ongoiba, Seydina Beye, Honoré Berthe, Mamadou Diakite, Mamadou Sima, Adégné Togo, Bakary Dembele, Djibo Diango, Moussa Samake, Youssouf Traoré, Louis Traoré, Ongoïba Oumar, Sogoba Gaoussou, Issaga Traoré, Sidy Sangaré, Doua Kanté, Lassana Cissé, Thiam Souleymane, Keita Koniba, Sundaresan Maiyalagan, Julien Chong, Adil Mohit, Khushyant Mungar, Shankaran Vinayagam, Kevin Ramlochun, Jamie Sim, Desai Sneha, Vishaal Kissoon, Yan Tseung, Mohamed Aboobakar, Nitish Fokeerah, Ravi Ramsewak, Jayprakash Gopall, Meetheelesh Abeeluck, Varun Seewoo, Divyanand Jankee, Ashveen Puryag, Senthil Beemadoo, Yashraj Deenoo, Abhisek Goureah, Munawwara Makoon, Hemanshu Rambojan, Beeharry Shanjugsingh, Kevin Viraswami, Shehzaad Joomye, Ashwant Bhugwandass, Bibi Deelawar, Vakil Leelodharry, Luckshmanraj Mungur, Sajid Aungraheeta, Sirsingh Bhajoo, Manpreet Rajcoomar, Rishi Seetaram, Subha Gaya, Gini Batra, Yoshvin Sunnassee, Shailendra Petkar, Sbai Hicham, Labib Smail, Ait Laalim Said, Motaai Youssef, Mouhssine Doumiri, Mustapha Alilou, Nora Farnaoui, Mustapha Bensghir, Abdelghafour Elkoundi, Abdelhamid Jaafari, Abderhmann Elwali, Mohammed Meziane, Walid Atmani, Houssam Rebahi, Hajar Chichou, Safae Zarouf, Abderraouf Soummani, Abou Elhassan Taoufik, Meryem Essafti, Aminata Oumou Traoré, Hamzaoui Hamza, Adnane Berdai, El allani Linda, Salhi Oussama, Nelson Mucopo, Machado Banze, Mouzinho Saide, Tomas Sitoi, Artur Machava, Antonio Carlos, Amilton Guidione, Antonio Saide de Carvalho, Natacha Gemo, Samiro Sema Camal, Arsénio Cuna, Ornelos Madeira, Ladino Assuade, Dercio Amde Fernandes, Dulce Alexandre Machavae Fernandes, Mandua Sebastião, Bernard Sikombe, Matti Kandjimi, Ayoub Shekimweri, Diana Shilomboleni, Mbaundju Kandjii, Leonard Kabongo, Cholastic Hangero, Ike Ndjoze, Ruben Nailonga, Immanuel Uukonga, Uutoni Nakanyala, Lavinia Johannes, Adrian Haruzuvi, Chris Terblanche, Natangwe Shimenda, Delwina Katjipu, Hilma Shalimba, Juliah Kaweendwa, Ali Mbuyi, John Oyedele, Mapumba Mulolo, Peter Njuki, Mutombo Ndaie, Akutu Munyika, Hilma Katangolo, David Tjiyokola, Tawanda Mhene, Archbald Masiambiri, Paidamoyo Mandudzo, Sandra Kapepiso, Haziel Mavesere, Cedia Tjihoto, Daylight Manyere, Charlotte Kauraisa, Learnmore Garanowako, Michael Tune, William DeKlerk, Benvenue Ndolo, Maria Angula, Ndapewoshali Hishekwa, Elizabeth Nandjendja, Elsabe Tsauses, Eunice Mouton, Kudzai Katandawa, Sophia Bruwer, Jaydee Van Staden, Beata Siteketa, Beata Kaholongo, Martha Ntinda, Pueya Nashidengo, Johanna Kandjumbwa, Lahia Lipumbu, Moussa Sirfi, Fouma Djibo, Moutari Mahaman, Abdoulaye Mahaman Bachir, Maikassoua Mamane, Adakal Ousseini, Maman Noury Hamissou Souley, Rabo Oumarou, Rekia Idrissa, Moussa Ichaou, Amina Saley, Abdoulay Seyni, Sahabi Amadou, Mahamane Sani Mahamane Laminou, Issoufou Moustapha Camara, Moussa Gagara, Hadjara Rabiou Daddy, Harissou Adamou, Ibrahim Amadou Magagi, Oumarou Habou, Sabo Ramatou, Saidu Kadas, Rabiu Mohammed, Abubakar Ballah, Tella Olalekan, Kefas Bwala, Mohammed Adamu, Adamu Isa, Ademola Adeyeye, Samuel Fayose, Akinola Akinmade, Taiwo Ajayi, Elizabeth Nwasor, Saidu Yakubu, Euphemia Ugwu, George Mukoro, Muhammed Ahmed, Gideon Akafa, Ahmad Lawal, Daniel Nwoye, Michael Odigbo, Zulaihatu Sarkin-Pawa, Tunde Sholadoye, Benjamin Fomete, Hamisu Yakubu, Abdulkadir Kabiru, Samaila Timothy, Ali Yusuf, Rabiu Mohammed, Tasiu Saadu, Babangida Mohammed, Abdulghaffar Yunus, Ganiyat Olagunju, Muhammad Aminu, Mohammad Idris, Musliu Tolani, Nasiru Dalhat, Samuel Gana, Talent Adike, Lofty-John Anyanwu, Abdurrahman Sheshe, Sani Aji, Mamuda Atiku, Raphael Attah, Abubakar Muhammed, Rasaki Oseni, Halima Salisu-Kabara, Benjamin Nkemjika, Omotayo Salami, Adekunle Akadri, Bukola Olayinka, Clement Onuoha, Umar Usman Jamaare, Auwalu Saminu Jibrin, Sani Giade Abdullahi, Ibrahim Ishaku, Adenike Odewabi, John Bamigboye, Oladapo Kuforiji, Chidiebere Ogo, Stella Ogunmuyiwa, Abdussemee Abdurrazzaaq, Adebayo Tanimola, Michael Adeyanju, Oluwatimilehin Andero, Temitope Ojo, Olusi Adedotun, Gbadamosi Kehinde, Jimoh Buraimoh, Kabiru Muhammad, Sophia Baidoo, Patrick Okoli, Azeez Adigun, Ekene Ezeonye, Kabir Isa, Yetunde Aremu-Kasumu, Kamil Shoretire, Peter Enesi, Amechi Ezike, Olatunde Olawoye, Emmanuel Ugwu, Christopher Ukah, Abolade Olugbenga, Nwachukwu Chidiebere, Nasiru Abdulraman, Adebiyi Olusegun, Alisa Halisa, Semiat Yusuf, Jamila Salisu, Chidiebube Okoro, Abdul Suleiman, Fabian Onowighose, Aliyu Farinyaro, Suleiman Baba, Umar Abdulmajid, Aisha Abdurrahman, Ogochukwu Obi, Olatunde Alabi, William Adeyemi, Jelili Salau, Jones Taiwo, Nnaemeka Nwafulume, Taiye Ibiyeye, Edith Agu, Ayodeji Danboy, James Abdulazeez, Christopher Ekwunife, Chimaobi Nnaji, Chigozirim Onyekpere, Amara Arunsi, Jude Egwim, Obianuju Nwana, Nnabuike Ojiegbe, Charles Mbamba, Paul Ngwu, Frank Imahigbe, Emmanuel Okoroji, Iloh Ikenna, Abdulrahman Mohammed, Adebayo Adeniyi, Toluwalope Ariyo, Olajide Gabriel, Tesleem Orewole, Salawu Idris, Idowu Adebara, Abiodun Okunlola, Akinwale Akinbade, Oluwasesan Afolabi, Adewumi Bakare, Olabisi Adeyemo, Benjamin Ugwu, Samuel Nuhu, Henry Embu, Erdoo Isamade, Chinedu Obikili, Amaka Ocheke, Solomon Peter, Donald Orshio, Peter Onuminya, Jack Okopi, Olufemi Bankole, Bosede Afolabi, Dapo Osinowo, Ayodeji Oluwole, Muyiwa Rotimi, Ibironke Desalu, Rufus Ojewola, Bolaji Mofikoya, Kola Owonikoko, Adeolu Adeoye, Temidayo Bobo, Taiwo Akinloye, David Ama, Ebere Okoronkwo, Muhammad Mahmud, Jamiu Adebiyi, Temitope Babalola, Mansur Muhammad, Afeez Aruna, Maryrose Osazuwa, Ayodeji Yusuf, Isiaka Lawal, Bitrus Fidelis, Rephath Pius, Cyril Jomosu, Adebayo Adedayo, Abubakar Aliyu, Adaora Agholor, Abdullateef Abdulazeez, Chabiya Bala, Eziamaka Eze, Elizabeth Ani, Uchenna Okeke, Dominica Adebayo, Okechukwu Ekwunife, Victor Modekwe, Chuka Ugwunne, Chukwuemeka Okoro, Chisom Uche, Simeon Olateju, Fred Ige-Orhionkpaibima, Adedapo Adetoye, Olurotimi Aaron, Jeremiah Abimbola, Folayemi Faponle, Olumuyiwa Ajayeoba, Olusoji Jagun, Oluwabunmi Fatungase, Adeniyi Akiseku, Chigbundu Nwokoro, Ramotalai Shoyemi, Ibukunolu Ogundele, Nankat Joseph, Salihu Bura, Chukwuka Nwezoku, Manu Bwala, Meshach Philips, Abubakar Usman, Emmanuel Filibus, Zara Umate, Nwabuoke Chukwuka, Ahmed Nuhu, Watakiri Ibrahim, James Nggada, Ali Izge, Musa Ismail, Olayinka Eyelade, Tinuola Adigun, Babatunde Osinaike, Olayinka Ogunbode, Olusola Idowu, Taiwo Lawal, Temidayo Ogundiran, Olayiwola Shittu, Omobolaji Ayandipo, Stephen Edino, Zumnan Songden, Olumide Akitoye, Bissallah Ekele, Godwin Akaba, Terkaa Atim, Akitoye Adeleke, Owoicho Okochi, Sunday Akeju, Ernest Ukpoju, Osayomwanbo Osaheni, Ifunanya Obaseki, Lateef Kehinde, Osawemwenze Monday, Stanley Nte, Adesuwa Agboifo, Omajuwa Dawodu, Precious Orhiere, David Atiti, Queeneth Kalu, Felix Effiom, Israel Kolawole, Olawale Ojo, Afusat Olabinjo, Olufemi Ige, Beatrice Ogunyemi, Olusola Oladosu, Kikelomo Adesina, Sulaiman Agodirin, Asimiyu Shittu, Audu Idrisa, Sadiq Adamu, Ahmed Nuhu, Nuhu Ali, Olayinka Adewunmi, Stephen Nwankwor, Akinwumi Olakanmi, Oluseye Ajayi, Ayotolu Ajayi, Victor Ogunmola, Oluwafunke Olakanmi, Adam Kuranga, Enoch Uche, Chukwuemeka Osuagwu, Chukwudi Ilo, Mesi Matthew, Uko Uko, Ngozi Mba, Olubusola Alagbe-Briggs, Amabra Dodiyi-Manuel, Bisola Onajin-Obembe, Bright Obasuyi, Richard Echem, Ihuoma Mike-Elechi, Job Otokwala, Mark Edubio, Catherine Eyo, Isaac Udo, Aliyu Abdulrahman, A.A. Abdullahi, Ibrahim Galadima Bello, Usman Adinoyin Mohammed, Abidemi Oyaromade, Mohammed Bello, Usman Muhammad, Emeri Mbah, Hyacinth Okereke, Almustapha Aminu, Anthony Ahmadu, Abu Rogers, Peter Samai, Sao Amara, Margaret Yankuba, Mary Josayah, Jayah Swarray Jnr, Alusine Dawo, Peter George, Mustapha Kabba, Mohamed Bah, Charles Mondeh, Ibrahim Kapuwa, Mohamed Sheku, Philip Mattia, Brima Sesay, Jones O.A. Omoshoro-Jones, Motselisi Mbeki, Estie Cloete, Philip Anderson, Busi Mrara, Annemarie Steyn, Tsakani Mhlari, Nic Proctor, Caroline Robertson, Gillian Lamacroft, Usha Singh, Sebenzile Sikhakhane, Kelly Gate, Shepherd Nzenza, John Tshimbalanga Kasonga, Sibongile Ndebele, Patrick Lufuta Kande, Jody Davids, Tino-vito Orlandi, Marischka de Jong, Hugo Stark, Francois Roodt, Jonathan Hall, Ian Nortje, Akanimo Akpakan, Vishendran Govindasamy, Ronisha Sathiram, Mohammad Kathrada, Zane Farina, Lucio Frittella, Charles Kohler, Sibuyiselwe Lubelwana, Sarwat Ikram-Hameed, Adriaan Smit, Muneerah Cassiem, Yvonne Freeman, Saaliha Goga, Larissa Cronje, Constantin Buzdugan, Subash Chirkut, Priyadeshni Singh, Sandhya Jithoo, Vivesh Rughubar, John Arnold, Rishan Bipath, Suman Mewa Kinoo, Ncumisa Khanyisa Msolo, Fleur Ackermans-Deijnen, Tshegofatso Mmasello Emma Boka, Martyn Biccard Greenwood, Shakthi Anand Jayrajh, Devarani Naidoo, Syndrini Reddy, Devandiran Harriraman Rungan, Kylene Subrayen, John Roos, Nina Tredoux, Pascal Plumacher, Anthony Reed, Harald Steinhaus, Mariesa Nock, Paul Ryan Herselman, Estie Cloete, Gareth Davies, Talitha Harvey, Franklin Muller, Willem Naude, Tania Pretorius, Johan Jochemus Swart, Merryn Walls, Prashant Gokal, Nicolette Rorke, Farzaana Dhoodhat, Precious Dzanibe, Mohammed Yusuf Hussain, Ashmita Junpath, Ameela Maharaj, Hylda Makanisi, Khalid Moosa, Ting Ting Wong, Sean Mould, Trisha Ramsamy, Roel Matos-Puig, Hayley Morgan, Nadeem Nabeebuccas, Ria Devi Naidoo, Viantha Pather, Vasheel Vasheel Bahadur, Renilda Pillay, Zahnne Fullerton, Nicole Bell, Bongisa Grey, Vincent Lorenzo Visentin, Hendrik Adriaan Van Zyl, Terri Anne Killingbeck, Emile Maneveldt, Gerhard Thiart, Magdelena May Venter, Oostewalt Swart, Mariette Grobelaar, Carel Cairns, David Bishop, Christien Steenkamp, Thandekile Khumalo, Noel Naidoo, Ross Murray, Martin Kopieniak, Melusi Sishange, Mxolisi Brian Ndimande, Megan Jaworska, Megan Jaworska, Sarwat Ikram, Bence Rainier, Renier J Liebenberg, Helena D Zwiegers, Philip M Nortje, Kamal Bhagwan, Estie Cloete, Margot Flint, Robert Dyer, Simone Adams, Yoshua Bwambale, Danny Ngomo, Patrice Kanku, Nivashen Pillay, Alexa de Castro, Atisha Maharaj, Janine Carim, Jenna Leigh Taylor, Karl M Köhne, Leanne W Drummond, Leanne Temlett, Lieze Geldenhuys, Lucy Rolt, Yvonne Seilbea, Kathryn Naidoo, Nicola A Kalafatis, Stefné Verwey, Thulile Biyase, Theroshnie Kisten, Belinda S Kusel, Timothy Craig Hardcastle, Richard Magagula, Christian Kampik, Kuzolunga Xulu, Sivuyisiwe Solala, Mia Sayed, Basil Enicker, Anil Madaree, Innocent Mukama, Gladmore Madombwe, Nonhlanhla Zulu, Nompumelelo Gasa, Nokuzula Kanjana, Sebenzile Buthelezi, Thembelihle Buthelezi, Andries Brink, Francois Potgieter, Busisiwe Mrara, Freddy Kabambi, Zaynab Alexander, Charles Choto, Paula Ima, Zintle Gxagxisa, Baphethuxolo Ningiza, Gillian Lamacraft, Jerome Mogorosi, Nadia du Plessis, Leonie de Man, Suné Thompson, Gerrit van Heerden, Edwin W Turton, Pieter M van der Linde, Josephine K Teme-Pitse, Reitumetse Tladi, Gillian D Saffy, Ene-Mari Roscher, Kristel Fortune, George Barnard, Tiisetso Makhasane, Evan Bowen, Akangcha Pal, Rachel Moore, Maria Fourtounas, Mary Augusta Adam, Renessa Arumugan, Gabriella Hyman, Jaclyn Jonosky, Maninginingi Makondo, Heveshan Moodley, Phillip Munda, Mzwandile Nyalungu, Victor Olusola, Sohan Zane Pinto, Tristan Pillay, Lucinda Singh, Paul Mwindekuma Wondoh, John Devar, Jones O.A. Omoshoro-Jones, Boitumelo Baloyi-Mnisi, Zach Koto, Tebogo Mabotja, Matlou Ernest Mabitsela, Sibongile Ruth Ndlovu, Branny Mthelebofu, Colin Beck, Matthew Dold, Alice Fan, Shannon MacQueen, Thembani Matabata, Catherine Mpehle, Charné Kulenkampf, Tsakani McCreath Mhlari, Simangele Cecilia Nyoka-Mokgalong, Felix Thumba Masinge, Randhir Ramnath Gunpath, Maropeng Petrus Pat Mothwa, Jo-Anne Asenath Mothwa, Danai Mhlanga, Jamie-Lyn Colly, Aunel Mallier Peter, Khalid Ben Hameda, Pulane Mokae, Stella Josephine Moumakoe, Kelechi Ekeh, Nezingu Lengo, Marnus Booyens, Inge Louise Seale, Pieter Daniel Theron, Nicolaas Abraham Schuman, Amber Carlyn Sonn, Jacobus Lukas Stander, Nadia Cloete, Marius Cloete, Catherine Ann Makepeace, Ronel van der Westhuizen, Leanne Robyn Messiahs, Amy Ruth Visagie, Fatima Vawda, Frans Christiaan Voster, Deepika Dhilraj, Oliver Smith, Stefan Bolon, Daniel Montwedi, Motsilisi Mbeki, Jayde Wyngaard, Mthunzi Ngcelwane, Thomas Kleyenstuber, Phyllis Phukubye, Liesel Schärf, Grace Laker, Elizabeth Semenya, Reinhard Dembskey, Thomas Tarlton, Tapiwa Jiri, Ngoie Hubert Mushid, Nhlanhla Samuel Ngwenya, Hazel Morongoa Mogodi, Carmen Sinevici, Anthony Osarogie Usenbo, Naledi Fodo, Anesu Chimini, Ntetelelo Sikobi, Sinovuyo Nokwange, Mluleki Noqhamza, Qumba Thembisa, Kajake Anantha Padmanabha Bhat, Rabin Mathew, Katrin Middleton, Abdus-sami Adewunmi, Craig Dickson, Humairah Bulbulia, Bianka Bester, Michelle de Klerk, Christia Benade, Francois Viljoen, Caroline Robertson, Monique Fischer, Khalid Alfaki, Abdalmalik Awad, Abdelsalam Algray, Mohammed Elsiddig, Suha Mohamed, Salih Mahmoud, Muhammed Osman, Asia Elgailany, Mazin Suliman, Hanaa Mohammed, Lina Aljeally, Mohammed Dirar, Mohammed Osman, Mazin Mohamed, Mohamed Elhasan, Abrar Widatalla, Abubakr Abubakr, Eman Mohamed, Alshareef Nour, Ntonto Doris Gama, Dolorosa Khetsiwe Shabangu, Cynthia Iradikunda, Samuel Mkoko, Paul Kisanga, Emmanuel Lema, Benson Lyimo, Mohamed Binde, Alphonce Chandika, Salim Salim, Sylvia Jumbe, Abel Makubi, Vihar Kotecha, Felician Kachinde, Museleta Nyakiroto, Emmanuel Jitambi, Venant Geofrey, Johaphes Josiah, Phinius Makubi, Frank Manumbu, Suzan Mlingwa, Ernest Ibenzi, Peter Mbelle, Kato Peleus, Enid Chiwanga, Nillah Richard, Shoo Leonard, Paulo Sanka, Subira Mushi, Bashir Nyangasa, Mohamed Janabi, Naizihijwa Majani, Pedro Palangyo, Evarist Nyawawa, William Ramadhan, Faraj Lydenge, Gileard Gabriel Masenga, Sakina Rashid, Mubashir Jusabani, Ansbert Ndebea, Jenitha Cheru, Margaret Henjewele, Greyson Kilimanjaro, Sarah Sikimata, Deocles Donatus, Hazina Maduhu, Tumaini Mariro, Given Massasi, Moshi Moshi Shabani, Braison Cholela, Marco Mgeleka, Yohatinus Mbilinyi, Faraja Chiwanga, Bilton Exavery, Caspar Haule, Samson Ndile, Sirili Harya, Julieth Magandi, Deogratius Manyama, Redempta Matindi, Adam Moshi, Daudi Kitwana, Merida Makia, Philip Muhochi, Miriam Herman, Clauda Miombo, Furaha Kahindo, Langtone Kishebuka, Elijah Ussiri, Gloria Kinasa, Patrick Adel, Eric Malaba, Vensesla Sakwari, Sadot Lugereka, Mohamed Mungia, George Mocha, Herman Wella, Cecilia Protas, Patrick Karua, Ahmada Kashagama, Faraja Mwasambugu, Suzana Kajeri, Jacquiline Mchilla, Elibariki Lucumay, Robert Maise, Amon Marti, Beatrice Mahundi, Frederika Jager, Charles Majani, Ludovick Rukeha, Tareeq Mohamed, Nabila Fuad, Winifrida Halinga, Elias Chrisant, Gilbert Msoma, Titus Kihwili, Gadiel Temu, Naima Yusuf, Rashid Saleh, Rashid Inoja, Eva Shang'a, Stella Ibrahim, Hussein Msuma, Edwin Edward, Paul Kilamile, Stephen Mwakyolile, Talkana Adja, Edem Gueouguede, Hafoudhoi Oussene Seddoh, Saliou Adam, Pilakimwe Egbohou, Mawunyo Ahomagnon, Olivier Kadjossou, Abdul-Bassiti Boukari, Mary T Nabukenya, Ruth Muhindo, Peter Waswa, Peter Kaahwa Agaba, Daphne Kabatoro, Joseph Kayong, Margaret Naggujja, Nabasiige Rehema, Phiona Nansubuga, Daniel Kavuma, Aggrey Lubikire, Hope Bisilikirwa, Godfrey Ssebaggala, Emmanuel Muwema, Humble Joan Agaba, John Kiconco, Nicholas Wataaka, Bonet Chan, Mary Juliet Nampawu, Fred Bulamba, Emmanuel Bua, Christine Mugala, Caroline Nyakato, John Paul Ochieng, Linda Kyomuhendo Jovia, George Kateregga, Rachel Alum, Lazia Najjuma, Gorret Nampiina, Andrew Kintu, Joshua Sempiira, Luzige Simon, Peter Kayima, Jacob Eyul, Erick Odwar, Rita Nkwine, Christine Namata, Elizabeth Nabakka, Denis Kakaire, Velda Mushangwe-Mtisi, Erisha Munhamo, Celestino Dhege, Juliet Hungwa, Hemish Jasi, Crispin Ntoto, Derek Matsika, Brightson Mutseyekwa, Joseph Zimbovoora, Beaulah Gudyanga, Dennis Mazingi, Chenesa Mbanje, Busisiwe Mlambo, Michael Chiwanga, Harunavamwe N Chifamba, Sarudzai Zhou, Esta Hove, Shamiso Dende, Beauty Manjengwa, Penias Kapisa, Mashava, Caritas Chiura, Locadia Katsukunya, Godfrey Muguti, Doreen Mashava, Elton Ndhlovu, Zanele Mangwangwa, Nombulelo Dube

## Abstract

**Background:**

Risk of mortality following surgery in patients across Africa is twice as high as the global average. Most of these deaths occur on hospital wards after the surgery itself. We aimed to assess whether enhanced postoperative surveillance of adult surgical patients at high risk of postoperative morbidity or mortality in Africa could reduce 30-day in-hospital mortality.

**Methods:**

We did a two-arm, open-label, cluster-randomised trial of hospitals (clusters) across Africa. Hospitals were eligible if they provided surgery with an overnight postoperative admission. Hospitals were randomly assigned through minimisation in recruitment blocks (1:1) to provide patients with either a package of enhanced postoperative surveillance interventions (admitting the patient to higher care ward, increasing the frequency of postoperative nursing observations, assigning the patient to a bed in view of the nursing station, allowing family members to stay in the ward, and placing a postoperative surveillance guide at the bedside) for those at high risk (ie, with African Surgical Outcomes Study Surgical Risk Calculator scores ≥10) and usual care for those at low risk (intervention group), or for all patients to receive usual postoperative care (control group). Health-care providers and participants were not masked, but data assessors were. The primary outcome was 30-day in-hospital mortality of patients at low and high risk, measured at the participant level. All analyses were done as allocated (by cluster) in all patients with available data. This trial is registered with ClinicalTrials.gov, NCT03853824.

**Findings:**

Between May 3, 2019, and July 27, 2020, 594 eligible hospitals indicated a desire to participate across 33 African countries; 332 (56%) were able to recruit participants and were included in analyses. We allocated 160 hospitals (13 275 patients) to provide enhanced postoperative surveillance and 172 hospitals (15 617 patients) to provide standard care. The mean age of participants was 37·1 years (SD 15·5) and 20 039 (69·4%) of 28 892 patients were women. 30-day in-hospital mortality occurred in 169 (1·3%) of 12 970 patients with mortality data in the intervention group and in 193 (1·3%) of 15 242 patients with mortality data in the control group (relative risk 0·96, 95% CI 0·69–1·33; p=0·79). 45 (0·2%) of 22 031 patients at low risk and 309 (5·6%) of 5500 patients at high risk died. No harms associated with either intervention were reported.

**Interpretation:**

This intervention package did not decrease 30-day in-hospital mortality among surgical patients in Africa at high risk of postoperative morbidity or mortality. Further research is needed to develop interventions that prevent death from surgical complications in resource-limited hospitals across Africa.

**Funding:**

Bill & Melinda Gates Foundation and the World Federation of Societies of Anaesthesiologists.

**Translations:**

For the Arabic, French and Portuguese translations of the abstract see Supplementary Materials section.

## Introduction

Surgical diseases represent a major part of the global public health burden.^1^ The *Lancet* Commission on Global Surgery was established to ensure the adequate provision of safe surgery for patients in low-income and middle-income countries.^2^ However, postoperative deaths are the third leading contributor to global mortality.^3^ Mortality is higher following surgery in low-income and middle-income countries than in high-income countries.^4^, ^5^, ^6^ The African Surgical Outcomes Study (ASOS) showed that the risk of mortality following surgery in patients across Africa was twice as high as the global average.^4^ Most deaths in Africa occur on hospital wards after surgery, suggesting that many lives could be saved through the early identification of postoperative physiological deterioration in surgical patients.^4^, ^7^

Physiological deterioration following postoperative complications and resulting in death is referred to as failure to rescue.^8^ Predisposing factors include low hospital volumes, low numbers of nursing staff,^9^ and scarce postoperative care facilities,^5^ which are characteristic of the surgical environment in Africa^4^ and provide an opportunity to rescue surgical patients from postoperative mortality.^5^ In high-income countries, failure to rescue is mitigated by systematic monitoring of surgical patients, facilitating early interventions to treat complications.^9^ It is unclear whether this approach is feasible or effective in African hospitals where resources are limited.

**Research in context**

**Evidence before this study**

Each year, 4·2 million people die worldwide within 30 days of surgery. Half of these deaths occur in low-income and middle-income countries. Postoperative deaths are the third leading contributor to global mortality. The first African Surgical Outcomes Study (ASOS) showed that, despite their low risk profile, the risk of mortality following surgery in patients across Africa was twice as high as the global average. Almost all these deaths occurred on hospital wards after surgery, suggesting that many lives could be saved by the early identification of patients at high risk with effective surveillance for physiological deterioration associated with postoperative complications. A literature review showed that the use of early warning systems inconsistently decreases mortality and morbidity resulting from in-hospital physiological deterioration in patients. This finding is attributed to the difficulty in escalating care, the role of clinical judgment in responding to deterioration, and the intermittent assessment of the patient. Although the application of early warning systems increases nurses' performance of care, the escalation of care needed by nurses and junior clinicians is dependent on organisational factors, and knowing how to respond to deterioration. To mitigate some of the barriers to the use of early warning systems, it might be appropriate to identify the patient at high risk before deterioration, increase surveillance for deterioration, and provide guidance on early management in the case of patient deterioration. We searched PubMed with no language restrictions on July 14, 2021, using the search terms: (“postoperative monitoring”[All Fields] OR “postoperative surveillance”[All Fields]) AND (“surgical procedures, operative”[MeSH Terms] AND “Postoperative Complications”[MeSH Terms]). We did a second search using the terms: (“early warning score”[MeSH Terms] OR (“early”[All Fields] AND “warning”[All Fields] AND “score”[All Fields]) OR “early warning score”[All Fields]) AND (“surgical procedures, operative”[MeSH Terms] AND “Postoperative Complications”[MeSH Terms]). Our literature review could not identify any trials that had adopted this approach with surgical patients. Globally, we did not identify any large randomised trials assessing the efficacy of enhanced postoperative surveillance on mortality in surgical patients at high risk.

**Added value of this study**

The principal finding of the ASOS-2 trial is that a package of five interventions to enhance postoperative surveillance for physiological deterioration among surgical patients at high risk in hospitals across Africa did not decrease mortality or the incidence of severe complications. Hospital staff were able to effectively assess risk, but implementation of enhanced postoperative surveillance proved to be more difficult than was expected. Effective delivery of the surveillance package required researchers to engage a wide number of key stakeholders to deliver these interventions. An inclusive approach to interprofessional collaboration was essential to the success or failure of the trial intervention. These findings suggest that detailed mixed-methods research is required to co-design postoperative surveillance interventions that can work within resource-limited hospitals in Africa. The ASOS-2 trial substantiates the feasibility of large international clinical trials of perioperative care in Africa, despite exceptional challenges such as armed conflicts.

**Implications of all the available evidence**

Death after surgery is an important public health problem in African countries. Mortality is largely driven by postoperative complications, such as sepsis and bleeding, in the hospital ward environment. Given the substantial financial and human resource requirements of providing perioperative critical care, there is an urgent need for novel solutions to prevent progression of postoperative complications in resource-limited environments. For these solutions to have a realistic chance of successful implementation in African hospitals, the interventions need to be carefully co-designed with local health-care staff to ensure sustainable adoption.

We hypothesised that failure to rescue was a major contributor to the high mortality of patients following surgery in Africa.^4^ A potential solution could be the early identification of patients at high risk of severe morbidity and mortality, who could be allocated to enhanced postoperative surveillance to identify physiological deterioration, promoting early management interventions. However, with scarce human resources, a reallocation of personnel time to patients at high risk might decrease the care of patients at lower risk and put them at increased risk of complications and mortality. Therefore, it is necessary to evaluate the effectiveness of any intervention that involves increased postoperative surveillance for patients at high risk in a resource-limited environment. In the ASOS-2 trial, we aimed to investigate whether a package of five interventions to enhance postoperative surveillance of adult surgical patients at high risk of severe morbidity and mortality in Africa could reduce 30-day in-hospital mortality.

## Methods

### Study design and participants

We did a two-arm, open-label, cluster-randomised trial of hospitals (clusters) across Africa. Hospitals in every African country that provided surgery with an overnight postoperative admission were eligible to participate. All participating hospitals fulfilled local ethics and regulatory requirements. We included consecutive patients aged at least 18 years undergoing elective and non-elective surgery who required an overnight admission. Participants who had previously participated in ASOS-2 were excluded. Ethics approval was obtained from the Human Research Ethics Committee of the University of Cape Town (HREC 081/2018). The primary ethics committee approved a waiver of consent, with the need to provide trial broadcasting signage at participating hospitals to ensure that all patients and family members were aware that the hospital was a trial site (appendix 4 p 43). Some local ethics committees required individual written patient consent for participation, which was obtained following randomisation. This report is prepared in accordance with the CONSORT extension for Cluster Trials.^10^ The trial was done in accordance with Good Clinical Practice guidelines.

### Randomisation and masking

Each hospital was a single cluster. Eligible hospitals were randomly assigned to an arm (1:1) through minimisation in recruitment blocks to provide either enhanced postoperative surveillance for surgical patients at high risk of severe morbidity and mortality and standard care for patients at low risk (intervention group) or standard care for all patients (control group). The first recruitment block of hospitals was block randomised in a 1:1 ratio, stratified by country and level of the surgical facility (ie, tertiary, secondary, and primary) with a fixed block size of two. Subsequent recruitment blocks of hospitals were allocated to treatment arms through minimisation, by allocating hospitals to study arm subject to balancing constraints. The algorithm was coded in R (version 3.4) and simulated a large number of random allocations, then selected the first allocation that met the balancing constraints when previous cycles of study arm allocation were accounted for (appendix 4 p 44). B M Biccard enrolled clusters, M Lesosky did the randomisation, and D van Straaten informed sites of allocation.

Health-care providers and participants were not masked to group allocation at the cluster or participant level; however, M Lesosky, who did the analyses, was masked to arm allocation. Unmasking only occurred when the masked output was signed off by M Lesosky, L Myer, and B M Biccard.

### Procedures

Hospitals randomly allocated to the control group were requested to provide usual postoperative care to all patients, which was left to the discretion of the health-care providers. Hospitals randomly assigned to the intervention group were requested to provide an intervention package to all adult surgical patients identified as being at high risk, which was defined as a score of at least 10 with the ASOS Surgical Risk Calculator,^11^ and usual care to patients with a risk score of less than 10 (ie, at low risk). Risk factors in the ASOS Surgical Risk Calculator include age and American Society of Anesthesiologists score, as well as the urgency, severity, indication, and type of surgery. The intervention package was developed through informal small group meetings of evidence-based medicine and implementation science held by a team predominantly comprised of trainees and specialists within the Department of Anaesthesia and Perioperative Medicine, University of Cape Town (Cape Town, South Africa). Key studies were thoroughly discussed, and the group considered the elements that were finally agreed on to be appropriate for the context and subsequently evaluated in a pilot trial of 803 patients from 16 hospitals in eight African countries (Benin, Democratic Republic of the Congo, Kenya, Mali, Mauritius, Niger, Nigeria, and South Africa) before the main trial.^12^ Data collection was completed in 772 (96%) patients. 21 (75%) of 28 hospital respondents believed that they had provided increased postoperative surveillance to patients at high risk, with 83 (66%) of 125 patients at high risk receiving some form of increased postoperative surveillance. The post pilot survey assessed the acceptability, appropriateness, and feasibility of the ASOS-2 intervention, with 63–87% of hospital respondents indicating agreement. The package consisted of providing as many of the five following enhanced postoperative surveillance interventions as possible: admitting the patient to a higher care ward than had been planned at the time of surgery, increasing the frequency of postoperative nursing observations, assigning the patient to a bed visible from the nursing station, allowing family members to stay with the patient in the postoperative ward, and placing a postoperative surveillance bedside guide in a visible position at the bedside. This guide contained information on the leading causes of postoperative mortality in surgical patients in Africa (ie, surgical site infections, bloodstream infection and acute respiratory distress syndrome, pneumonia, acute kidney injury, postoperative bleeding, and cardiac arrest), with advice on clinical management if the patient were to deteriorate following surgery (appendix 4 p 45). All hospitals were encouraged to provide the interventions for as long as possible after surgery, but the specific nature and duration of the enhanced postoperative surveillance were at the discretion of local health-care staff.

We collected data describing all adult surgical patients at low and high risk. With the onset of the COVID-19 pandemic, the trial could only continue at a hospital if participation did not increase the risk of SARS-CoV-2 transmission among patients or investigators. Additionally, the intervention involving the family staying at the patient's bedside was removed from the enhanced postoperative surveillance package.

ASOS-2 was a pragmatic trial designed for a resource-limited environment. To minimise the impact of the trial on clinical services, we asked each hospital to either recruit up to 100 consecutive patients or to recruit for a maximum of 4 weeks, if this number was not reached. Data collection was limited to a one-page case record form, with only the primary outcome requiring verification by supporting documentation (appendix 4 pp 46–47). Sites were informed of their allocation arm approximately 4 weeks before the recruitment start date. Sites received an arm-specific presentation and checklist, which listed objectives for each week of the site initiation (appendix 4 p 48). Site initiation was signed off by an online test consisting of nine questions. Automated WhatsApp communications were developed by Praekelt, a non-profit mobile communication organisation based in Africa. Data were submitted via an online REDCap database;^13^ however, investigators could submit data to the coordinating centre for entry onto the database.

To assess study implementation, we did a prospective, mixed-methods process evaluation. A qualitative evaluation of the barriers to and facilitators of intervention delivery was done using a comparative case study approach in three countries (South Africa, Uganda, and Sierra Leone), which included ten hospitals. We interviewed hospital lead investigators, study investigators (including anaesthetists, surgeons, and ward nurses), and some hospital administrators. A quantitative evaluation of the trial implementation was done with a close-out questionnaire, including items on team composition, the trial dummy run, the component interventions of the ASOS-2 package, and 21 Likert questions testing potential influences on intervention delivery, to all hospitals in the intervention group. Due to limited internet access, sites in the Democratic Republic of the Congo were not able to participate in the post-trial questionnaire. The full results of the process evaluation will be published in a separate publication, and a summary is presented in this report.

### Outcomes

The primary outcome was in-hospital mortality for all patients (at low and high risk), censored at 30 days after surgery if the patient was still alive and in hospital. The secondary outcome was a composite of severe in-hospital complications and mortality for all patients (at low and high risk), censored at 30 days after surgery if the patient was still alive and in hospital. Both outcomes were measured at the participant level. Severe complications were defined as any of the following: surgical site or body-cavity infection, bloodstream infection or acute respiratory distress syndrome, urinary tract infection or acute kidney injury, non-fatal cardiac arrest, pneumonia, postoperative bleed, or any other complication defined as severe (appendix 4 pp 49–51). The primary outcome was verified by uploading de-identified supporting data. There was no change to the primary outcome following trial commencement. The trial design is summarised in appendix 4 (p 52).

### Statistical analysis

The sample size was informed by the ASOS trial.^4^ We considered a 25% relative risk reduction in mortality for all surgical patients to be clinically important. To decrease mortality from 2·0% to 1·5%, with a conservative intracluster correlation coefficient for the composite of severe complications and mortality of 0·015 (compared with 0·01 in ASOS), and a coefficient of variance of 0·63 for a 4-week recruitment period, the trial required 64 200 patients from 642 hospitals offering surgery across Africa. The sample size calculations for the ASOS-2 trial were based on a power of 80% (two-sided α=0·05) and a mean cluster size of 100 patients (appendix 4 p 53).^4^

A statistical analysis plan was written and published on ClinicalTrials.gov before trial completion, without access to any data. All clusters and patients were analysed according to the treatment arm to which they were originally allocated. The primary analysis was a modified intention-to-treat analysis, which included all patients recruited from randomised hospitals where the hospital had reported any patient data. Hospitals that were randomised but did not submit any patient data were not included in the modified intention-to-treat analysis on the assumption that there was no risk of exposure to the trial intervention.

For the primary effectiveness outcomes, we did a complete case analysis, excluding patients with missing data from the analysis. For the effectiveness outcomes, the risk ratio (RR) was estimated by univariable generalised estimating equation under a binomial model with a log link, assuming an exchangeable correlation structure. Clustering was assumed to be on hospitals within countries in a fully nested framework. Categorical variables are described as proportions and continuous variables are described as mean (SD) or median (IQR). Statistical analyses were done with SPSS (version 24) and R (version 3.4).

Prespecified secondary analyses included two analytical approaches to per-protocol populations based on the implementation fidelity of the enhanced postoperative surveillance intervention. In the first per-protocol analysis, we compared all patients from hospitals with data in the control group with all patients from hospitals with data in the intervention group, whereby the hospital had provided the intervention with fidelity to at least 80% of patients at high risk. Patients from hospitals where the intervention was provided with fidelity to less than 80% of patients at high risk were excluded. In the second per-protocol analysis, we compared all patients from hospitals with data in the control group with all patients in the intervention group from hospitals in the top two tertiles of implementation fidelity. Patients from intervention hospitals in the bottom tertile of implementation fidelity were excluded. We reported the hospital-level implementation fidelity as the proportion of patients at high risk who had received the intervention with fidelity. We used two definitions for implementation fidelity: provision of at least the high-risk bedside guide plus one additional component of the intervention on days 0 and 1 after surgery (definition 1) and provision of at least any two components of the intervention on days 0 and 1 after surgery, which did not necessarily have to include the high-risk bedside guide as one of the components (definition 2). Other prespecified sensitivity and subgroup analyses on the effectiveness outcomes are shown in appendix 4 (p 54).

The ethics committee waived the requirement for a Data and Safety Monitoring Board, given that the intervention package was considered to be low risk by The Human Research Ethics Committee of the Faculty of Health Sciences, University of Cape Town. However, an independent international adviser was appointed to the trial (Paul Myles, Department of Anaesthesiology and Perioperative Medicine, Alfred Hospital and Monash University, Melbourne, VIC, Australia), whose role was to decide whether hospital recruitment could continue after the planned recruitment window, and whether an interim analysis would be required before continuing the recruitment process in the event that the enrolment took longer than expected. The trial exceeded the planned recruitment period, and the international adviser supported continued recruitment without an interim analysis.

This trial is registered with ClinicalTrials.gov, NCT03853824, where the full protocol is publicly available. There were no major changes to the protocol after the initial ethical approval of version 2 (appendix 4 pp 37–42).

### Role of the funding source

The funders of the study had no role in study design, data collection, data analysis, data interpretation, or writing of the report.

## Results

Between May 3, 2019, and July 27, 2020, we randomly assigned 577 hospitals across 33 African countries, with 290 (50%) hospitals allocated to the intervention group and 287 (50%) hospitals to the control group (figure). 245 hospitals did not recruit following randomisation, predominantly because of failed stakeholder engagement and ethical approvals (111 [45%]; figure). The trial was stopped early when the COVID-19 pandemic made it difficult to recruit further hospitals, and surgery was severely curtailed to prepare for the pandemic. COVID-19 restrictions were identified on March 17, 2020, and took effect on March 18, 2020, after which no further hospitals were recruited. 5395 participants were recruited after the restrictions took effect; 2891 participants in the control group and 2504 participants in the intervention group.

**Figure:**
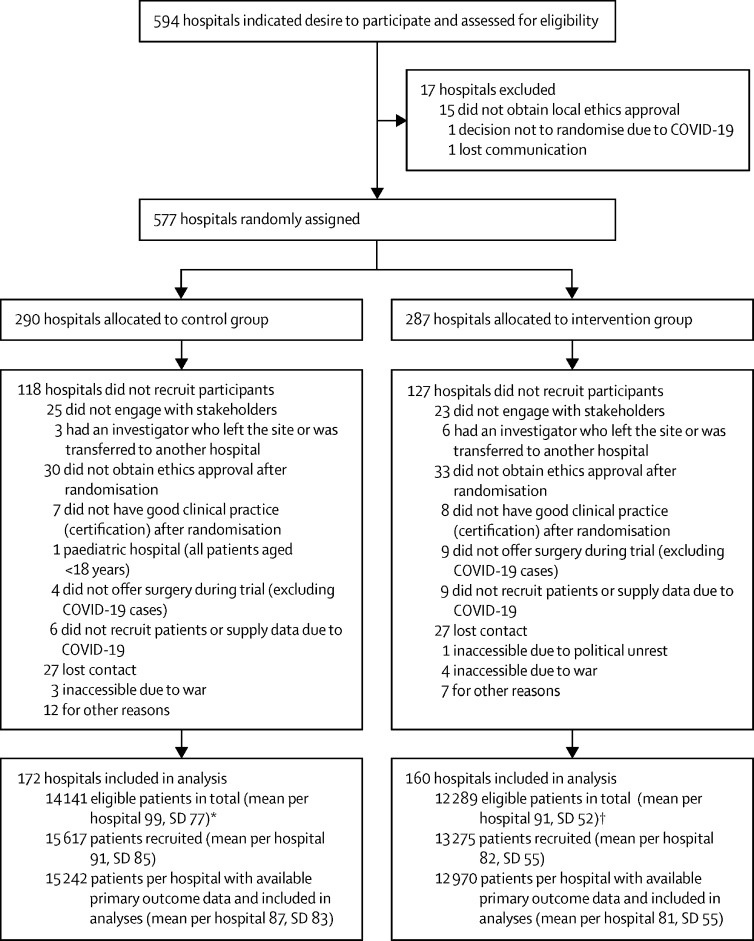
Trial profile *143 (83%) of 172 hospitals in the control group provided data on total number of eligible patients. †135 (84%) of 160 hospitals in the intervention group provided data on total number of eligible patients.

332 hospitals from 28 African countries (Angola, Benin, Burkina Faso, Burundi, Democratic Republic of the Congo, Djibouti, Egypt, Eswatini, Ethiopia, The Gambia, Ghana, Kenya, Libya, Malawi, Mali, Mauritius, Morocco, Mozambique, Namibia, Niger, Nigeria, Sierra Leone, South Africa, Sudan, Tanzania, Togo, Uganda, and Zimbabwe) recruited 28 892 patients onto the trial (of a planned sample size of 64 200 patients). 172 hospitals allocated to the control group recruited 15 617 patients and 160 hospitals allocated to the intervention group recruited 13 275 eligible patients. 245 hospitals, which also included hospitals from six other countries, were randomly assigned to groups but did not recruit. The hospitals and participants recruited per randomisation wave are shown in appendix 4 (p 55). The mean number of participants recruited per cluster was 87 (SD 73), with a coefficient of variation of 0·83. The majority of participants (14 952 [51·8%]) were recruited from tertiary hospitals (appendix 4 p 56). Hospital-level data were submitted for 184 hospitals, including 39 (21%) primary-level, 67 (36%) secondary-level, and 77 (42%) tertiary-level hospitals. Hospitals had a median of 344 beds (IQR 172–585), including four critical care beds (0–6) providing invasive ventilation (table 1). Hospitals were staffed by a median of four specialist surgeons (IQR 1–11), four specialist anaesthetists (0–10), and four specialist obstetricians (0–10; appendix 4 p 57). The median number of surgical ward patients was 25 (10–40), with a nurse–patient ratio of 1:8 (1:12–1:3) during the day, and 1:12 (1:13–1:5) during the night.

**Table 1:** Baseline cluster-level characteristics of hospitals and individual-level characteristics of patients

		**Intervention group (n=13 275)**	**Control group (n=15 617)**
**Individual-level data**			
Age, years			
	Mean (SD)	37·4 (15·8)	36·8 (15·3)
	Median (IQR)	33 (26–45)	33 (26–43)
	Data missing	39 (0·3%)	41 (0·3%)
Sex			
	Male	4119 (31·0%)	4495 (28·8%)
	Female	9059 (68·2%)	10 980 (70·3%)
	Data missing	97 (0·7%)	142 (0·9%)
**Comorbidity**			
Hypertension		2097 (15·8%)	2286 (14·6%)
	Data missing	160 (1·2%)	120 (0·8%)
HIV or AIDS		1059 (8·0%)	1151 (7·4%)
	Data missing	174 (1·3%)	125 (0·8%)
Diabetes		844 (6·4%)	872 (5·6%)
	Data missing	175 (1·3%)	123 (0·8%)
Chronic obstructive pulmonary disease or asthma		273 (2·1%)	325 (2·1%)
	Data missing	176 (1·3%)	123 (0·8%)
**American Society of Anesthesiologists category**			
1		5691 (42·9%)	7181 (46·0%)
2		5861 (44·2%)	6522 (41·8%)
3		1300 (9·8%)	1445 (9·3%)
4		196 (1·5%)	287 (1·8%)
5		21 (0·2%)	25 (0·2%)
Data missing		206 (1·6%)	157 (1·0%)
**Grade of surgery**			
Minor		2054 (15·5%)	2085 (13·4%)
Intermediate		7491 (56·4%)	8397 (53·8%)
Major		3646 (27·6%)	4963 (31·8)
Data missing		84 (0·6%)	172 (1·1%)
**Urgency of surgery**			
Elective		5648 (42·5%)	6232 (39·9%)
Urgent		3544 (26·7%)	4129 (26·4%)
Emergency		4009 (30·2%)	5109 (32·7%)
Data missing		74 (0·6%)	147 (0·9%)
**Indication for surgery**			
Non-communicable disease		5015 (37·8%)	5342 (34·2%)
Caesarean section		4961 (37·4%)	6224 (39·9%)
Trauma		1906 (14·4%)	1990 (12·7%)
Infection		1267 (9·5%)	1442 (9·2%)
Data missing		126 (0·9%)	619 (4·0%)
**Surgical speciality**			
Obstetrics		5211 (39·3%)	6554 (42·0%)
Gynaecology		1267 (9·5%)	1539 (9·9%)
Orthopaedic		1754 (13·2%)	1989 (12·7%)
Plastics and breast		439 (3·3%)	502 (3·2%)
Urology		666 (5·0%)	712 (4·6%)
Ear, nose, and throat		314 (2·4%)	228 (1·5%)
Gastrointestinal and hepatobiliary		1841 (13·9%)	2263 (14·5%)
Cardiothoracic and vascular		254 (1·9%)	263 (1·7%)
Neurosurgery		273 (2·1%)	335 (2·1%)
Other		1174 (8·8%)	1097 (7·0%)
Data missing		82 (0·6%)	135 (0·9%)
**Risk stratification**			
ASOS Surgical Risk Calculator score		6·37 (4·14)	6·38 (4·20)
	Data missing	242 (1·8%)	675 (4·3%)
High risk (ASOS score ≥10)		2548 (19·2%)	3025 (19·4%)
	Data missing	242 (1·8%)	675 (4·3%)
**Hospital-level data**			
Hospitals with data		82 (51·3%)	102 (59·3%)
Hospitals missing data		78 (48·8%)	70 (40·7%)
Hospital beds		322 (190–593)	347 (150–585)
	Data missing	1 (1·2%)	0
Operating theatres		4 (3–6)	4 (2–7)
	Data missing	0	0
Critical care beds providing invasive ventilation		4 (0–6)	4 (1–6)
Critical care beds without invasive ventilation		4 (0–8)	3 (1–6)
	Data missing	2 (2·4%)	4 (3·9%)
Physician anaesthesiologist		6 (1–12)	4 (0–10)
Physician surgeon		5 (1–12)	4 (0–10)
Physician obstetrician		4 (2–10)	4 (0–11)
	Data missing	0	0

Data are median (IQR), mean (SD), or n (%). ASOS=African Surgical Outcomes Study.

The mean age of patients overall was 37·1 (SD 15·5) years, 69·4% were women and 29·6% were men (with 0·8% missing data on sex), and 87·4% were physical status category 1 or 2 according to the American Society of Anesthesiologists (table 1). 16 791 (58·1%) of 28 892 surgeries were urgent or emergent, and 24 497 (84·8%) surgeries were intermediate or major.

In the intervention group, risk stratification using the ASOS Surgical Risk Calculator was done in 13 033 (98·2%) of 13 275 patients: preoperatively in 4778 (36·0%) patients, intraoperatively in 3767 (28·4%), postoperatively in 4514 (34·0%), and at an unknown timepoint in 216 (1·6%) patients. Approximately a fifth of patients were stratified as being high risk (ie, ASOS score ≥10; table 1). The patient ASOS Surgical Risk Calculator scores are shown in appendix 4 (p 58). The risk calculator performed as expected, with a large patient subgroup at low risk (22 402 [80·1%] of 27 975 patients with an ASOS Surgical Risk Calculator score) and a smaller subgroup at high risk (5573 [19·9%] of 27 975), with 45 (0·2%) deaths of 22 031 patients and 1171 (5·4%) severe complications of 21 832 patients among the low-risk subgroup, and 309 (5·6%) deaths of 5500 patients and 1308 (24·0%) severe complications of 5444 patients in the high-risk subgroup. The number of postoperative interventions implemented in the intervention group is shown in appendix 4 (p 59). The individual components of the intervention package delivered postoperatively are shown in table 2. At least one component of the intervention was provided to more than 95% of the patients at high risk on days 0 and 1 postoperatively. The proportion of intervention hospitals that provided the intervention with fidelity to more than 80% of patients at high risk was 40·0% (64/160) according to definition 1 of implementation fidelity, and 59·4% (95/160) according to definition 2.

**Table 2:** Individual intervention components of enhanced postoperative surveillance for patients at high risk in hospitals providing intervention

	**Patients at high risk (n=2548)**
Admitting the patient to a higher care ward than had been planned before surgery	1288 (50·5%)
Increasing the frequency of postoperative nursing observations	2144 (84·1%)
Assigning the patient to a bed visible from the nursing station	1799 (70·6%)
Allowing family members to stay with the patient in the ward	1075 (42·2%)
Placing a postoperative surveillance bedside guide in a visible position at the bedside	2008 (78·8%)

Data are n (%).

All 28 892 individuals from 332 hospitals were included in the primary analysis, of whom 27 850 (96·4%) were discharged alive or were alive in hospital at day 30, 362 (1·3%) died in hospital, and 680 (2·4%) had missing primary outcome data. The primary outcome of 30-day in-hospital mortality occurred in 169 (1·3%) of 12 970 patients in the intervention group and in 193 (1·3%) of 15 242 patients in the control group (RR 0·96, 95% CI 0·69–1·33; p=0·79; table 3). The secondary outcome of a composite of in-hospital severe complications and death occurred in 1150 (9·0%) of 12 819 patients in the intervention group and in 1405 (9·3%) of 15 182 patients in the control group (0·96, 0·75–1·23; p=0·76). The individual components of the secondary outcome are shown in table 4. No other adverse events were reported. Recoding of unobserved outcomes as either assumed alive or dead did not change the effectiveness estimates (appendix 4 p 60). There were no harms or unintended effects reported in either arm. The trial results are visually summarised in appendix 4 (p 69). The intracluster correlation coefficient was 0·009 (95% CI 0·007–0·01) for the primary outcome and 0·07 (0·05–0·09) for the secondary outcome.

**Table 3:** Effect of enhanced postoperative surveillance for surgical patients at high risk on in-hospital outcomes of all surgical patients included in the modified intention-to-treat analysis

	**Intervention group (n=13 275)**	**Control group (n=15 617)**	**Relative risk (95% CI)**	**p value**
**Primary outcome**				
In-hospital mortality	169/12 970 (1·3%)	193/15 242 (1·3%)	0·96 (0·69–1·33)	0·79
Missing outcome data	305	375	..	..
**Secondary outcome**				
In-hospital severe complications and death	1150/12 819 (9·0%)	1405/15 182 (9·3%)	0·96 (0·75–1·23)	0·76
Missing outcome data	456	435	..	..

**Table 4:** Effect of enhanced postoperative surveillance for surgical patients at high risk on the individual components of the secondary outcome

		**Intervention group (n=13 275)**	**Control group (n=15 617)**
Superficial or deep surgical site, or body cavity infection		459 (3·5%)	664 (4·3%)
	Data missing	323 (2·4%)	314 (2·0%)
Bloodstream infection or acute respiratory distress syndrome		148 (1·1%)	155 (1·0%)
	Data missing	303 (2·3%)	311 (2·0%)
Urinary tract or acute kidney injury		142 (1·1%)	149 (1·0%)
	Data missing	321 (2·4%)	316 (2·0%)
Cardiac arrest		101 (0·8%)	138 (0·9%)
	Data missing	326 (2·5%)	318 (2·0%)
Pneumonia		117 (0·9%)	149 (1·0%)
	Data missing	321 (2·4%)	316 (2·0%)
Postoperative bleed		190 (1·4%)	314 (2·0%)
	Data missing	324 (2·4%)	313 (2·0%)
Other severe complications		368 (2·8%)	327 (2·1%)
	Data missing	343 (2·6%)	334 (2·1%)

Data are n (%).

5573 patients were classified as high risk, of whom 73 (1·3%) had a missing primary outcome. Eight (2·2%) of 362 deaths had missing risk classification data. In-hospital mortality occurred in 309 (5·6%) of 5500 patients who were classified as high risk. Of the high-risk patients, 149 (5·9%) of 2523 died in the intervention group, and 160 (5·4%) of 2977 died in the control group (RR 1·11, 95% CI 0·88–1·39; p=0·39). There were 22 402 patients who were classified as low risk, of whom 371 (1·7%) had a missing primary outcome. In-hospital mortality occurred in 45 (0·2%) of 22 031 patients classified as low risk. Of the low-risk patients, 19 (0·2%) of 10 277 patients in the intervention group, and 26 (0·2%) of 11 754 patients in the control group died (0·84, 0·46–1·51; p=0·55).

Prespecified per-protocol secondary analyses of implementation fidelity of the trial intervention were consistent with the primary outcome and were not associated with decreased in-hospital mortality (appendix 4 p 61) or in-hospital severe complications (appendix 4 p 62). The primary effectiveness outcomes for the stratification variables of hospital level, recruitment wave, and income category of country (appendix 4 p 63), as well as individual patient and surgical characteristics for the entire cohort (appendix 4 pp 64–67) and patients at high risk (appendix 4 p 68), were consistent with the primary analysis.

The process evaluation identified variation in local hospital trial preparation, with 62 (69%) of 90 respondents considering their colleagues sufficiently prepared to deliver the ASOS-2 interventions, whereas nine (10%) disagreed. Variation was also identified in the receptiveness of the trial within the local context, and was associated with variation in delivery of intervention components. Most hospital leaders led small teams to deliver the interventions and collect trial data. Motivation was high among hospital leaders who saw the project as important; however, the qualitative case studies identified several contextual mediators that influenced the teams' ability to deliver the intervention. The risk assessment outputs of the ASOS Surgical Risk Calculator were not always trusted when they did not fit with individual clinicians' views of the patient's risk. Although 52 (58%) of the 90 respondents reported that the ward nursing staff found the workload of the ASOS-2 interventions acceptable, the assumption that additional physiological observations could be delivered as a cost-neutral intervention by ward nurses was challenged. Even though 61 (68%) of 90 respondents reported that nurses saw value in the ASOS-2 interventions, this work was still expressed as an additional burden. Most local teams found managing the data collection and intervention delivery a challenge that required more than the limited resources available. The quantitative analysis showed that 36 (40%) of 89 respondents found that they spent more time on data collection than on implementing the ASOS-2 interventions; however, 30 (34%) of 89 respondents disagreed that they spent more time on data collection than on implementation.

The questionnaire data completed after the trial confirmed the associations between these influences and the intervention fidelity across the trial. Teams reporting a belief in the effectiveness of the ASOS-2 intervention, as well as nursing and surgical staff engagement, were associated with greater intervention fidelity. Conversely, when teams reported spending more time on data collection than on implementing the interventions, there was lower fidelity. Furthermore, the element of the ASOS-2 intervention designed to facilitate the translation of increased surveillance into detection and treatment of complications (ie, the bedside guide) was not perceived as impactful.

## Discussion

The principal finding of this large pragmatic trial was that an intervention to promote enhanced postoperative surveillance for patients at high risk of postoperative severe morbidity or mortality did not decrease in-hospital mortality or the incidence of severe complications among adult surgical patients in Africa. However, the enhanced postoperative surveillance intervention was not associated with additional harm relative to usual care. Providing enhanced care to surgical patients at high risk in an environment with limited personnel also did not increase the postoperative risk for patients at low risk.

Failure to rescue (death following a postoperative complication) is an important quality improvement indicator,^8^ which shows differences in outcomes at a national^14^ and international level,^15^ and explains some of the increased mortality following surgery in Africa.^4^ Albeit small, pre-intervention and post-intervention studies have reported mixed outcomes when escalating therapy in response to early warning signs.^9^, ^16^ Simulation studies have shown that the use of cognitive aids (similar to the ASOS-2 bedside guide) can decrease the omission of critical management steps,^17^ with fewer critical errors.^18^ Feedback on patient outcomes can reduce major adverse events following surgery.^19^ Although these studies from well resourced environments provide support for the trial hypothesis, the ASOS-2 intervention did not decrease postoperative mortality in a low-resource environment.

The following factors might have contributed to the inability of the ASOS-2 intervention to improve surgical outcomes. Although the trial intervention was designed to limit service demands in an environment with a limited health-care workforce, the intervention is considered to be a complex intervention due to the need for behaviour change and the involvement of multiple stakeholders.^20^ Process evaluation data suggest that delivering enhanced postoperative surveillance was a challenge requiring considerable energy from site investigators and strong teamwork. Communication was identified as a barrier to the escalation of care in patients with physiological deterioration.^9^ Completing this trial in a low-resource environment on a fixed budget presented considerable barriers because it required clinicians to divert time away from clinical care to trial-related tasks, which could have negatively affected the implementation of interventions. The risk calculator was easy to use, and the performance of the risk calculator in the trial was consistent with the original derivation study, which reported an incidence of severe complications of 16·6% (95% CI 14·9–18·4).^12^ However, clinicians did not always accept the risk stratification. Furthermore, the bedside guide was not considered impactful by some clinicians. High-volume hospitals^21^ and high levels of nursing staff^22^ also mitigate against failure to rescue. These systems and resource factors could have contributed to the inability to implement the intervention successfully.^4^ Inadequate postoperative facilities could have impeded implementation.^5^ Although the ASOS-2 trial did not prevent death following surgery in Africa, it is important to continue to investigate interventions for surgical patients with physiological deterioration in Africa, given that surgical volume estimates in sub-Saharan Africa^23^ suggest that over 6·2 million surgeries are done per year.

This trial has some strengths. First, the study provides external validation of the utility of the ASOS Surgical Risk Calculator.^12^ Second, directing care to patients at high risk only did not increase mortality of surgical patients at low risk in a resource-limited environment. Third, it is likely that these results are generalisable to adult surgery across Africa because there were no exclusion criteria and the trial included 322 hospitals of all levels from 28 African countries across various human development index rankings. Fourth, the process evaluation provides insight into context-specific factors that need to be addressed to ensure implementation fidelity of an intervention. Finally, this trial represents a large network of clinician investigators who are willing to collaborate to investigate potential interventions in a resource-limited environment.^24^ The learning gained through participation in this large continental trial (eg, compliance and completion of regulatory, ethical, and good clinical practice requirements) provides a powerful collaborative platform to build on. However, research and trial capacity need to be strengthened, including local regulation in Africa, given that 33·1% of non-participation was due to an inability to provide adequate ethical or good clinical practice materials.

There are limitations to this trial. First, despite the pragmatic design, the sample size was not achieved for several reasons, including the COVID-19 pandemic, armed conflicts, and failure to obtain regulatory approvals. Failure to recruit a sufficient number of hospitals might have been in part due to the limited local resources to carry out the trial, which could be partially overcome by increased funding. Second, in providing an intervention package that could be randomised to clusters and applied over a relatively brief time period, we might have underestimated the effort needed to change and measure the performance of a complex intervention within a health system. Third, we could not directly measure the difference in care between individual patients in the intervention and those in the control groups; therefore, we are unable to establish whether the increased postoperative surveillance resulted in increased management interventions for patients with postoperative complications. Furthermore, structural barriers might have limited the ability to provide postoperative management. These limitations could have affected the trial endpoints independently of the trial intervention. As the health-care providers were not masked, it is possible that intervention compliance was also over-reported. Fourth, an intervention package for caesarean section specifically might have resulted in improved outcomes in women following this procedure. Although caesarean sections contributed to 11 185 (38·7%) of the 28 892 total surgical procedures, the objective of the ASOS-2 trial was to identify a generalisable intervention for adult surgical patients in Africa, and the sensitivity analysis for caesarean section was consistent with the overall trial findings for the primary effectiveness outcome. Fifth, there might have been performance bias associated with the co-intervention in the control arm of the trial, selection bias in the patients whose outcomes were not reported, or both, because the point estimate of mortality reported in ASOS-2 (1·3% [362/28 212]) was lower than that reported in ASOS (2·1% [239/11 193]; p<0·0001).^4^ Finally, the pragmatic trial design limited our analysis to in-hospital outcomes. However, approximately 30% of all deaths within 30 days of surgery occur after hospital discharge globally,^7^ and we expect a higher proportion of patients to die following discharge in resource-limited environments.^4^, ^25^

The key learning points from this study are that future attempts to provide pragmatic interventions for surgical patients with physiological deterioration in a resource-limited environment will need to be co-designed by all local role players to ensure appropriate buy-in with the risk stratification strategy, teamwork necessary to implement the intervention, application of cognitive aids,^17^ communication of risk within the team,^9^ and feedback on outcomes and performance.^19^ Implementation strategies for resource-limited environments must include the use of educational meetings, tailoring and practising interventions, leverage of local leaders, and provision of feedback to change health-care provider behaviour.^26^ These strategies are important as the trial intervention had a low implementation fidelity. The difficulty with implementing the intervention in every hospital might also be due to the widely differing contexts between hospitals.

This trial showed that diverting human resources to patients at high risk does not increase morbidity or mortality for patients who are not risk stratified as at high risk, even though they might receive less care as a result. However, the pragmatic nature of the ASOS-2 trial does not allow us to understand whether the intervention of increased postoperative surveillance improves outcomes, or whether the inability to respond to postoperative complications (eg, infections or bleeding) limits efficacy. Based on the absence of harm to patients at low risk, a quality improvement programme in the form of an implementation effectiveness study for patients at high risk might be warranted and appropriate.

Any interventional perioperative research in Africa requires strong teamwork, an understanding of the working environment, and strategies to increase the fidelity of intervention implementation. It might be appropriate to focus further research on surgical patients at high risk only. Adequate funding is necessary to support this research.

## Data sharing

Data will be disclosed only upon request and approval of the proposed use of the data by the steering committee (B M Biccard, H-L Kluyts, M Lesosky, L Myer, L du Toit, P Forget, T Stephens, and R M Pearse). Data are available to the journal for evaluation of reported analyses. Data requests from other non-ASOS-2 investigators will not be considered until 2 years after the close out of the trial. Data will be de-identified for participant, hospital, and country, and will be available with a signed data access agreement.

## Declaration of interests

R M Pearse reports grants from Edwards Lifesciences and Intersurgical; and personal fees from Edwards Lifesciences and GlaxoSmithKline, outside of the submitted work. R M Pearse also reports being a member of the editorial boards of the *British Journal of Anaesthesia* and the *British Journal of Surgery*. A B A Prempah was the recipient of the World Federation of Societies of Anaesthesiologists–International Anesthesia Research Society Clinical Research Fellow in Global Surgery and Anaesthesia in Africa. All other authors declare no competing interests.

## Supplementary Materials

Arabic translation of the abstract

French translation of the abstract

Portuguese translation of the abstract
