# Supplementary material for: Enhanced postoperative surveillance versus standard of care to reduce mortality among adult surgical patients in Africa (ASOS-2): a cluster-randomised controlled trial
Source: Lancet Glob Health. 2021 Aug 19;9(10):e1391–401. doi: 10.1016/S2214-109X(21)00291-6 (PMC8440223; doi:10.1016/S2214-109X(21)00291-6)
Supplement: Arabic translation of the abstract [file mmc1.pdf]

THE LANCET
Global Health

Supplementary appendix 1

This translation in Arabic was submitted by the authors and we reproduce it as supplied. It has not been peer reviewed. *The Lancet*'s editorial processes have only been applied to the original in English, which should serve as reference for this manuscript.

تم تقديم هذه الترجمة باللغة العربية من قبل المؤلفين ونعيد إنتاجها كما هو مُقدم. إنها لم تخضع لاستعراض الأقران. تم تطبيق عمليات
تحرير لانسيت فقط على النص الأصلي باللغة الإنجليزية، والذي يجب أن يكون بمثابة مرجع لهذه المخطوطة.

Supplement to: The ASOS-2 Investigators. Enhanced postoperative surveillance versus standard of care to reduce mortality among adult surgical patients in Africa (ASOS-2): a cluster-randomised controlled trial. *Lancet Glob Health* 2021; published online Aug 18. http://dx.doi.org/10.1016/S2214-109X(21)00291-6.

Enhanced postoperative surveillance versus standard of care to reduce mortality among adult surgical patients in Africa (ASOS-2): a cluster-randomised controlled trial

The ASOS-2 Investigators
*****

المراقبة المحسنة بعد الجراحة مقابل معيار الرعاية الاساسية لتقليل الوفيات بين مرضى الجراحة البالغين في إفريقيا (ASOS-2): تجربة عنقودية منضبطة معشاة ذات شواهد

باحثون ASOS-2 *

ملخص الدراسة باللغة العربية

المعرفة السابقة: خطر الوفاة بعد الجراحة في المرضى في جميع أنحاء أفريقيا هو ضعف المعدل العالمي. تحدث معظم هذه الوفيات في أجنحة المستشفى بعد الجراحة نفسها.
لقد هدفنا إلى تقييم ما إذا كان تعزيز مراقبة ما بعد الجراحة للمرضى البالغين الخاضعين لعملية جراحية و المعرضين لخطر كبير للإصابة بالأمراض أو الوفيات بعد الجراحة في إفريقيا واذا كان بالامكان ان يتم تقليل معدل الوفيات في المستشفى لمدة 30 يومًا.
طريقة البحث: قمنا بتجربة عشوائية عنقودية ثنائية الذراع ، مفتوحة التسمية ، لمستشفيات (مجموعات) في جميع أنحاء إفريقيا. كانت المستشفيات مؤهلة إذا قدمت الجراحة مع البقاء في المستشفى بعد الجراحة. تم تعيين المستشفيات بشكل عشوائي من خلال التقليل في مجموعات التوظيف (1: 1) لتزويد المرضى إما بحزمة من تدخلات المراقبة المحسنة بعد الجراحة (قبول المريض في جناح رعاية أعلى ، وزيادة تواتر ملاحظات التمريض بعد الجراحة ، وتعيين المريض في سرير خاص تحت المراقبة قرب طاقم التمريض ، السماح لأفراد الأسرة بالبقاء في الجناح الطبي قرب المريض ، ووضع دليل مراقبة ما بعد الجراحة بجانب السرير) لأولئك المعرضين لخطر كبير (على سبيل المثال ، مع درجات عليا مقدرة ب10 فما فوق حسب حاسبة المخاطر الجراحية الأفريقية لدراسة النتائج الجراحية والرعاية المعتادة للمرضى في خطر منخفض (مجموعة التدخل) ، أو لجميع المرضى لتلقي الرعاية المعتادة بعد الجراحة (مجموعة المراقبة). لم يكن مقدمو الرعاية الصحية والمشاركين محجوبين بخصوص معرفة طريقة البحث ، لكن مقيمي البيانات كانوا كذلك. كانت النتيجة الأولية هي معدل الوفيات في المستشفى لمدة 30 يومًا للمرضى عند مستوى منخفض ومخاطر عالية ، تقاس على مستوى المشاركين. تم إجراء جميع التحليلات حسب التوزيع (حسب المجموعة) في جميع المرضى مع البيانات المتاحة. هذه التجربة مسجلة في ClinicalTrials.gov برقم NCT03853824 .
النتائج: بين 3 مايو 2019 و 27 يوليو 2020 ، أشارت 594 مستشفى مؤهلًا إلى رغبتها في المشاركة عبر 33 دولة أفريقية ؛ تمكن 332 (56٪) من تجنيد المشاركين وتم تضمينهم في التحليلات اللإحصائيات. خصصنا 160 مستشفى

(13275 مريضًا) لتوفير مراقبة محسنة بعد الجراحة و 172 مستشفى (15617 مريضًا) لتوفير الرعاية القياسية. كان متوسط عمر المشاركين 37.1 سنة بانحارف معياري 5 الى 15 و 20039 (69.4%) كانوا من النساء.
حدثت الوفيات في المستشفى خلال مدة 30 يومًا في 169 (1.3%) من اصل 12970 مريضًا مع بيانات الوفيات في مجموعة التدخل وفي 193 (1.3%) من اصل 15224 مريضًا مع بيانات الوفيات في المجموعة الضابطة و (الاختطار النسبي) كان 0.96 مع فاصل ثقة مقدر مابين 0.69 الى 1.33 وقيمة p مقدرة ب0.79. توفي 45 (0.2%) من اصل 22031 مريضًا في خطر منخفض و 309 (5.6%) من اصل 5500 مريض معرضون لمخاطر عالية. لم يتم الإبلاغ عن أي أضرار مرتبطة بأي من التدخلات.
التفسير: لم تنقص حزمة التدخل هذه معدل الوفيات في المستشفى خلال 30 يومًا بين مرضى الجراحة في إفريقيا المعرضين لخطر كبير للمراضة أو الوفيات بعد الجراحة. هناك حاجة إلى مزيد من البحث لتطوير التدخلات التي تمنع الموت من المضاعفات الجراحية في المستشفيات محدودة الموارد في جميع أنحاء أفريقيا.
