# Supplementary material for: Enhanced postoperative surveillance versus standard of care to reduce mortality among adult surgical patients in Africa (ASOS-2): a cluster-randomised controlled trial
Source: Lancet Glob Health. 2021 Aug 19;9(10):e1391–401. doi: 10.1016/S2214-109X(21)00291-6 (PMC8440223; doi:10.1016/S2214-109X(21)00291-6)
Supplement: French translation of the abstract [file mmc2.pdf]

THE LANCET
Global Health

Supplementary appendix 2

This translation in French was submitted by the authors and we reproduce it as supplied. It has not been peer reviewed. *The Lancet*'s editorial processes have only been applied to the original in English, which should serve as reference for this manuscript.

Cette traduction en français a été proposée par les auteurs et nous l'avons reproduite telle quelle. Elle n'a pas été examinée par des pairs. Les processus éditoriaux du *Lancet* n'ont été appliqués qu'à l'original en anglais et c'est cette version qui doit servir de référence pour ce manuscrit.

Supplement to: The ASOS-2 Investigators. Enhanced postoperative surveillance versus standard of care to reduce mortality among adult surgical patients in Africa (ASOS-2): a cluster-randomised controlled trial. *Lancet Glob Health* 2021; published online Aug 18. http://dx.doi.org/10.1016/S2214-109X(21)00291-6.

Surveillance postopératoire renforcée comparée à une prise en charge habituelle pour réduire la mortalité chez les patients chirurgicaux adultes en Afrique (ASOS-2): un essai contrôlé randomisé par grappes

Résumé

Contexte
Le risque de mortalité après chirurgie en Afrique équivaut au double de la moyenne mondiale. La plupart de ces décès surviennent dans les services hospitaliers après l'acte chirurgical. Notre objectif était d'évaluer si une surveillance postopératoire renforcée des patients chirurgicaux adultes à haut risque de morbidité ou de mortalité postopératoire en Afrique pouvait réduire la mortalité hospitalière à 30 jours.

Méthodes
Nous avons réalisé un essai a deux bras, en ouvert, randomisé par grappes (chacune étant un hôpital), à travers l'Afrique. Les hôpitaux étaient éligibles s'ils effectuaient des interventions chirurgicales avec hospitalisation postopératoire d'au moins une nuit. Les hôpitaux ont été randomisés par minimisation en blocs (1:1) pour, soit fournir aux patients un ensemble d'interventions de surveillance postopératoire renforcée (admission du patient dans une unité de soins dédiée à des soins renforcés, fréquence accrue des observations infirmières postopératoires, affectation du patient à un lit visible depuis le bureau infirmier, possibilité pour les membres de la famille de rester dans le service, et placement d'un guide de surveillance postopératoire au chevet du patient) pour les patients à haut risque (c'est-à-dire avec un score ≥10 à l'African Surgical Outcomes Study Surgical Risk Calculator) et des soins habituels pour les patients à faible risque (groupe d'intervention), soit, pour tous les patients, des soins postopératoires habituels (groupe témoin). Les professionnels de santé et les participants n'étaient pas en aveugle, mais les analystes l'étaient. Le critère de jugement principal était la mortalité hospitalière à 30 jours des patients à faible et à haut risque, mesurée au niveau des participants. Toutes les analyses ont été effectuées suivant le groupe alloué (par grappe) incluant tous les patients pour lesquels des données étaient disponibles. Cet essai est enregistré sur ClinicalTrials.gov, numéro NCT03853824.

Résultats
Entre le 3 mai 2019 et le 27 juillet 2020, dans 33 pays africains, 594 hôpitaux éligibles ont manifesté un intérêt à participer. Parmi eux, 332 (56%) ont pu recruter des participants et ont été inclus dans les analyses. Nous avons affecté 160 hôpitaux (13 275 patients) à une surveillance postopératoire renforcée et 172 hôpitaux (15 617 patients) aux soins standards. L'âge moyen des participants était de 37,1 ans (DS 15,5) et 20 039 (69,4 %) des 28 892 patients étaient des femmes. La mortalité hospitalière à 30 jours était de 169 (1,3%) sur 12 970 patients avec des données de mortalité dans le groupe d'intervention et de 193 (1,3%) sur 15 242 patients avec des données de mortalité dans le groupe témoin (risque relatif 0,96, IC à 95 % 0,69-1,33 ; p = 0,79). 45 (0,2 %) des 22 031 patients à faible risque et 309 (5,6 %) des 5 500 patients à haut risque sont décédés. Aucun effet secondaire associé à l'une ou l'autre des interventions n'a été signalé.

Interprétation
Cet ensemble d'interventions n'a pas réduit la mortalité hospitalière à 30 jours chez les patients chirurgicaux en Afrique à haut risque de morbidité ou de mortalité postopératoire. Des recherches supplémentaires sont nécessaires pour développer des interventions

capables de réduire les décès dus à des complications chirurgicales dans les hôpitaux à ressources limitées à travers l'Afrique
