# Supplementary material for: Enhanced postoperative surveillance versus standard of care to reduce mortality among adult surgical patients in Africa (ASOS-2): a cluster-randomised controlled trial
Source: Lancet Glob Health. 2021 Aug 19;9(10):e1391–401. doi: 10.1016/S2214-109X(21)00291-6 (PMC8440223; doi:10.1016/S2214-109X(21)00291-6)
Supplement: Portuguese translation of the abstract [file mmc3.pdf]

THE LANCET
Global Health

Supplementary appendix 3

This translation in Portuguese was submitted by the authors and we reproduce it as supplied. It has not been peer reviewed. The Lancet's editorial processes have only been applied to the original in English, which should serve as reference for this manuscript.

Esta tradução em português foi submetida pelos autores e nós não fizemos quaisquer alterações. Esta versão não foi revista por pares. O processo editorial do The Lancet só foi aplicado à versão original em inglês, que deve servir como referência para este artigo.

Supplement to: The ASOS-2 Investigators. Enhanced postoperative surveillance versus standard of care to reduce mortality among adult surgical patients in Africa (ASOS-2): a cluster-randomised controlled trial. *Lancet Glob Health* 2021; published online Aug 18. http://dx.doi.org/10.1016/S2214-109X(21)00291-6.

Resumo/Sumário

Melhoria da vigilância pós-operatória versus cuidados estandardes, na reducão da mortalidade em pacientes cirúrgicos adultos em África: ensaio clínico randomizado por grupos.

Os investigadores ASOS-2.

Antecedentes

O risco de mortalidade pós-cirúrgica em África é duas vezes maior que a do resto do mundo. Maior parte das mortes ocorrem nos serviços de internamento.

Com este estudo gostaríamos de saber se o aumento da vigilância pós-operatória de pacientes adultos com alto risco cirúrgico de morbilidade e mortalidade em África, poderia reduzir a mortalidade hospitalar nos 30 dias de pós-operatório imediato.

Metodologia

Estudo randomizado, em grupos de hospitais em africanos, divididos em, grupo dos que tinham capacidade de oferecer cuidados intensivos e aqueles que poderiam sómente prestar cuidados estandardes básicos.

Os hospitais eram elegíveis segundo a capacidade de cirúrgias e de internamento.

Estes hospitais, eram avaliados segundo a capacidade de implementar o pacote de intervenção (grupo de intervenção) de aumento da vigilância pós-operatória (admissão em cuidados intensivos, aumento da frequência de observação pós-operatória de enfermagem, colocação de pacientes próximos a sala de enfermagem, permissão de companhia familiar no internamento e afixar guião de vigilância no cabeçário do leito do paciente) para os pacientes de risco (isto é segundo o score da African Surgical Outcomes Study Surgical Risk Calculator ≥10), ou em aqueles em que os pacientes recebiam os cuidados pós-operatórios usuais (grupo control).

Os provedores de cuidados e participantes não eram anonimizados como era o acesso a toda informação colhida.

O resultado era a mortalidade hospitalar pós-operatória nos 30 dias imediatos, dos pacientes com baixo e alto risco.

Este estudo ficou registrado como ClinicalTrials.gov, número NCT03853824.

Resultados

Entre 3 de Maio 2019 a 27 de Julho de 2020, houve a participação de 594 hospitais de 33 países africanos, destes, 332 (56%) foram considerados elegíveis e incluídos no estudo.

Destes, 160 hospitais (13 275 pacientes) forneciam informações sobre a capacidade de prover vigilância pós-operatória incrementada e outros 172 (15617 pacientes) sobre a capacidade de prover cuidados estandardes.

A média de idade dos participantes foi de 37·1 anos (SD 15·5) e 20039 (69·4%) de 28892 pacientes eram do sexo feminino. A mortalidade hospitalar em 30 dias do pós-operatório

imediato ocorreu em 169 (1·3%) dos 12 970 pacientes com informação sobre mortalidade no *grupo de intervenção* e em 193 (1·3%) dos 15242 pacientes com informação sobre mortalidade no *grupo control* (risco relativo 0·96, 95% CI 0·69–1·33; p=0·79).

Faleceram 45 (0·2%) dos 22 031 pacientes de baixo risco e 309 (5·6%) dos 5500 pacientes de alto risco. Não foi registado nenhum prejuízo associado com esta intervenção.

Interpretação

Em África, este pacote de intervenção não reduz a mortalidade hospitalar nos 30 dias do pós-operatório imediato dos pacientes adultos cirúrgicos com alto risco de morbilidade e mortalidade. Mais investigação/pesquisa é necessária para o desenvolvimento de intervenções que interfiram na prevenção da mortalidade por complicações cirúrgicas, em países africanos de baixos recursos.

Financiamento:

Fundação Bill & Melinda Gates e Federação Mundial das Sociedades de Anestesistas.

Atílio Morais
