# Supplementary material for: Enhanced postoperative surveillance versus standard of care to reduce mortality among adult surgical patients in Africa (ASOS-2): a cluster-randomised controlled trial
Source: Lancet Glob Health. 2021 Aug 19;9(10):e1391–401. doi: 10.1016/S2214-109X(21)00291-6 (PMC8440223; doi:10.1016/S2214-109X(21)00291-6)

THE LANCET
Global Health

Supplementary appendix 4

This appendix formed part of the original submission and has been peer reviewed. We post it as supplied by the authors.

Supplement to: The ASOS-2 Investigators. Enhanced postoperative surveillance versus standard of care to reduce mortality among adult surgical patients in Africa (ASOS-2): a cluster-randomised controlled trial. *Lancet Glob Health* 2021; published online Aug 18. http://dx.doi.org/10.1016/S2214-109X(21)00291-6.

b

Effect of enhanced postoperative surveillance on mortality amongst adult surgical patients in Africa: A cluster randomised trial.

The ASOS-2 Investigators

Supplementary material

Table of Contents

Appendix. Members of the African Surgical OutcomeS-2 Trial (ASOS-2) group 3

Supplementary Material S1. ASOS-3 Protocol amendments 37

Supplementary Material S2. Broadcasting document 43

Supplementary Material S3. Randomisation algorithm 44

Supplementary Material S4. Postoperative surveillance bedside guide 45

Supplementary Material S5. Case record form: Control arm 46

Supplementary Material S6. Case record form: Intervention arm 47

Supplementary Material S7. Site initiation checklists 48

Supplementary Material S8. Definitions of the components of the secondary outcome 'severe complications' 49

Supplementary Material S9. The ASOS-2 trial design 52

Supplementary Material S11. Prespecified sensitivity and subgroup analyses 54

Supplementary Table S1. Hospitals and participants recruited per randomisation wave in the trial 55

Supplementary Table S2. Hospitals recruited in the trial 56

Supplementary Table S3. Physician surgeons, anaesthesiologists and obstetricians, and the number of operating theatres in hospitals recruited in the trial 57

Supplementary Table S4. ASOS Surgical Risk Calculator Scores in participating patients 58

Supplementary Table S5. Number of postoperative surveillance interventions provided to high-risk patients 59

Supplementary Table S6. Sensitivity analyses of the potential impact of unobserved outcomes on the primary and secondary effectiveness estimates, where individuals with unobserved outcomes were alternately assumed to be alive at discharge (censored at thirty days) or to have died in-hospital within 30 days 60

Supplementary Table S7. Per protocol analyses of enhanced postoperative surveillance implementation fidelity to decrease in-hospital mortality. 61

Supplementary Table S8. Per protocol analyses of enhanced postoperative surveillance implementation fidelity to decrease in-hospital severe complications and death 62

Supplementary Table S9. Stratified models for primary effectiveness outcome associated with enhanced postoperative surveillance 63

Supplementary Table S10. Sensitivity analyses for primary effectiveness outcome associated with enhanced postoperative surveillance 64

Supplementary Table S11. Sensitivity analyses for primary effectiveness outcome associated with enhanced postoperative surveillance for high-risk patients 68

Supplementary Figure 1. The African Surgical OutcomeS-2 (ASOS-2) trial results 69

Appendix. Members of the African Surgical OutcomeS-2 Trial (ASOS-2) group

Writing Committee: Bruce M Biccard, Leon du Toit, Maia Lesosky, Tim Stephens, Landon Myer, Agya Prempeh, Nicola Vickery, Hyla-Louise Kluyts, Alexandra Torborg, Akinyinka Omigbodun, Adesoji Ademuyiwa, Muhammed Elhadi, Mohamed Elfagieh, Bernard Mbwele, Mpoki Ulisubisya, Lazaro Mboma, Daniel Z Ashebir, Mahlet Tesfaye Bahta, Hassen Mohammed, Mikiyas Teferi, Yakob Seman, Eugene Zoumenou, Adam Hewitt-Smith, Janat Tumukunde, Dolly Munlemvo, Atilio Morais, Apollo Basenero, Pisirai Ndarukwa, Nazinigouba Ouerdraogo, Maman Sani Chaibou, Mohyeddine Zarouf, Ahmed Rhassane El Adib, Veekash Gobin, Zimogo Sanogo, Youssouf Coulibaly, Zipporah Ngumi, Tarig Fadalla, Cynthia Lule, Venerand Barendegere, Isaac Smalle, Mustapha Bittaye, Ahmadou Lamin Samateh, Mahmoud Elfiky, Maher Fawzy, Wakisa Mulwafu, Vanessa Msosa, Lygia Lopes, Akwasi Antwi-Kusi, Sama Hamza, Patrice Forget, Dawid van Straaten, Rupert M Pearse

1. Prof Bruce M Biccard PhD, Professor and 2nd Chair, Department of Anaesthesia and Perioperative Medicine, Groote Schuur Hospital, Faculty of Health Sciences, University of Cape Town, South Africa
2. Leon du Toit MMed (Anaes), Department of Anaesthesia and Perioperative Medicine, Groote Schuur Hospital, Faculty of Health Sciences, University of Cape Town, South Africa
3. Maia Lesosky PhD, Division of Epidemiology & Biostatistics, School of Public Health & Family Medicine, University of Cape Town, South Africa.
4. Tim Stephens, Improvement Scientist // Nurse Researcher, Honorary Clinical Senior Lecturer // Florence Nightingale Scholar, Critical Care and Peri-operative Medicine Research Group, WHRI, Queen Mary University of London, United Kingdom.
5. Professor Landon Myer PhD, Division of Epidemiology & Biostatistics, School of Public Health & Family Medicine, University of Cape Town, South Africa.
6. Agya BA Prempeh FCA (SA), Department of Anaesthesia and Perioperative Medicine, Groote Schuur Hospital, Faculty of Health Sciences, University of Cape Town.
7. Nicola Vickery FCA(SA), Department of Anaesthesia and Perioperative Medicine, Groote Schuur Hospital, Faculty of Health Sciences, University of Cape Town, South Africa
8. Prof Hyla Kluyts DMed (Anaest), Department of Anaesthesiology, Sefako Makgatho Health Sciences University, South Africa
9. Alexandra Torborg FCA(SA), Discipline of Anaesthesiology and Critical Care, Nelson R. Mandela School of Medicine, University of KwaZulu-Natal, Congella, KwaZulu-Natal, South Africa
10. Prof Akinyinka Omigbodun FWACS, Professor of Obstetrics and Gynaecology, College of Medicine, University of Ibadan, Nigeria
11. Prof Adesoji O Ademuyiwa, FACS, Professor of Surgery (Paediatric and Surgical Epidemiology), Department of Surgery, College of Medicine, University of Lagos and Lagos University Teaching Hospital, Lagos, Nigeria
12. Muhammed Elhadi MBBCh, Faculty of Medicine, University of Tripoli, University Road, Furnaj, 13275 Tripoli, Libya
13. Prof Mohamed Ahmed Elfagieh FRCS MD, NCI, Misrata, Libya
14. Bernard Mbwele. MD, MSc, PhD Candidate, Birmingham University, Department of Epidemiology, University of Dar es Salaam - Mbeya College of Health and Allied Sciences (UDSM-MCHAS), P.O Box 608 Mbeya, Tanzania
15. Mpoki Ulisubisya MMed (Anaesthesia and Critical Care), Muhimbili University of Health and Allied Sciences, Dar Es Salaam Tanzania.
16. Lazaro Mboma MMed, Department of Surgery, Mbeya Zonal Referral Hospital, P.O Box 419 Mbeya, Tanzania
17. Prof Daniel Z Ashebir, Depart of Surgery, School of Medicine, CHS, Addis Ababa University, Ethiopia
18. Mahlet Tesfaye Bahta Cert(Cardiothoracic Surgery), Assistant Professor, Addis Ababa University, College of Health Science, School of Medicine, Department of Surgery, Ethiopia
19. Hassen Mohammed MPH, Director of Health Services Quality, Federal Ministry of Health, Addis Ababa, Ethiopia
20. Mikiyas Teferi DM, National Coordinator for Surgical Improvement Case Team, Ministry of Health, Ethiopia
21. Yakob Seman A MPH, Director General, Medical Services, Ministry of Health, Ethiopia
22. Prof Eugene Zoumenou, Faculté des Sciences de la Santé de Cotonou, Hôpital de la mère et de l'enfant, Lagune de Cotonou, Benin

23. Adam Hewitt-Smith MBBS, Lecturer, Department of Anaesthesia & Critical Care, Busitema University Faculty of Health Sciences, Uganda
24. Janat Tumukunde MMed (Anaesthesia), Anaesthesiologist, Makerere University, Kampala, Uganda
25. Dolly Munlemvo MD, Specialist in Anaesthesiology and Resuscitation, University Hospital of Kinshasha, Democratic Republic of Congo; Research Associate, Department of Anesthesiology and Pain Medicine, Nationwide Children's Hospital Columbus/Ohio, USA
26. Atilio Morais PhD, Cardiothoracic and Vascular Surgeon, Departamento de Cirurgias (Hospital Central de Maputo and Faculdade de Medicina, Universidade Eduardo Mondlane), Maputo, Moçambique
27. Apollo Basenero MBChB, Ministry of Health and Social Services Namibia, Private Bag 13198, Windhoek, Namibia, Namibia
28. Pisirai Ndarukwa PhD, Department of Public Health, University of KwaZulu Natal SA; Department of Health Sciences, Bindura University of Science Education, Zimbabwe
29. Professor Nazinigouba OUERDRAOGO, Chef du Departement d'anesthésie réanimation, CHU Yalgado Ouagadougou, Burkina Faso.
30. Maman Sani CHAIBOU, Anaesthesiologist and Chief of Department of Anesthesiology, Intensive Care and Emergency, National Hospital of Niamey, Republic of Niger
31. Mohyeddine Zarouf MD, Hospital Mohammed V, Tangier, Morocco
32. Prof Ahmed Rhassane El Adib MD, Educational Manager of Marrakesh Simulation and Innovation in Health Sciences Center, Head of Anesthesia and Critical Care in Gynecology -Obstetrics Department Mother and Child Hospital, Mohammed VI University Hospital School of Medicine, Cadi Ayyad University, Marrakesh Morocco
33. Veeksah Gobin MD Anaesthesia, Ministry of Health and Wellness, Jawaharlal Nehru Hospital (JNH), Rose Belle, Mauritius
34. Prof Zimogo Sanogo, Chief of Department of Surgery, CHU du Point G, Faculté of Medecine, Bamako, Mali
35. Prof Youssouf Coulibaly, Professor and Chief of Department, Faculté de médicine de Bamako, Mali
36. Zipporah Ngumi, FFARCS, Chair Department of Anaesthesia, University of Nairobi School of Medicine, Kenya
37. Tarig Fadalla MBBS, Ribat Neurospine Center, Ribat University Hospital, The National Ribat University. Khartoum, Sudan.
38. Cynthia Iradukunda MBChB, Mbabane Government Hospital, eSwatini.
39. Vénérand Barendegere MMed (General Surgery), FCS-ECSA, Department of Surgery, Kamenge Military Hospital, Bujumbura, Burundi.
40. Isaac O Smalle MD MWACS, Division of Breast and Oncology, Connaught Hospital, Ag. Head Department of Anatomy, College of Medicine and Allied Health Sciences, University of Sierra Leone, Sierra Leone.
41. Mustapha Bittaye, Senior Lecturer Obstetrics & Gynaecology, University of The Gambia, The Gambia.,
42. Ahmadou L Samateh FWACS, Senior Lecturer in Surgery, University of The Gambia, The Gambia
43. Mahmoud Elfiky MD, Kasr Al Ainy Faculty of Medicine, Cairo University, Ali Ibrahim St, Manial, Cairo 11432.
44. Prof Maher Fawzy MD, Emeritus Professor of Anesthesia , ICU and Pain Management, Faculty of Medicine, Cairo University, Egypt
45. Wakisa Mulwafu PhD, Associate Professor, College of Medicine, Private Bag 360, Blantyre, Malawi.
46. Vanessa Msosa FCS(ECSA), General Surgeon, Kamuzu Central Hospital, P.O. Box 149, Lilongwe, Malawi
47. Professor Lygia Lopes, General surgeon, Clínica Sagrada Esperança, Luanda, Angola.
48. Akwasi Antwi-Kusi, FGCS, Senior Lecturer, Department of Anaesthesiology and Intensive Care, School of Medical Sciences, College of Health Sciences, Kwame Nkrumah University of Science and Technology, Kumasi, Ghana
49. Hamza D Sama, Maître assistant, Chef service anesthésie reanimation, CHU Sylvanus Olympio, 08 BP 8146 Lomé, Togo
50. Prof Patrice Forget PhD, Institute of Applied Health Sciences, Epidemiology group, School of Medicine, Medical Sciences and Nutrition, University of Aberdeen; Department of Anaesthesia, NHS Grampian, Aberdeen, United Kingdom.
51. Dawid EA van Straaten BA, Safe Surgery South Africa NPC, 5 14th Street, Parkhurst, Gauteng, 2193, South Africa.

52. Prof Rupert M Pearse MD(Res), Professor of Intensive Care Medicine, Queen Mary University of London, United Kingdom, EC1M 6BQ

Steering Committee: BM Biccard, H Kluyts, M Lesosky, L Myer, L du Toit, P Forget, T Stephens, RM Pearse on behalf of African Surgical OutcomeS-2 (ASOS-2) Investigators

Process Evaluation Team: T Stephens, L du Toit, NJ Vickery, A Torborg, M Puchert, L Rolt, H Kluyts, D van Straaten, RM Pearse, BM Biccard

Other Central Trial Team Members: D van Straaten, A Prempeh, K Schwebler, F Kabambi, T Mabotja

ASOS-2 Investigators:

*Hospital Lead Investigator

Angola. National Leader: Lygia Lopes
Clínica Sagrada Esperança
Lygia Lopes*
Leandys Cobas
Albino Freitas

Hospital Central Dr. António Agostinho Neto
Maria Antunes*
Bartolomeu Cabo
Domingos Paulo
Carlos Camongua

Benin. National Leader: Eugene Zoumenou
Hôpital de Zone de Kandi
Bodourin Dossou-Yovo*
Chantal Segla
Mohamed Toko

Centre Hospitalier Universitaire De Zone Abomey Calavi
Yvette Avognon*
Osseni Marcos
Raymond Kintomonho
Onesime Demahou
Gisèle Hounsa

Hôpital d'Instruction des Armées/ Centre Hospitalier et Universitaire de Parakou
Julien Attinon*
Roger Klikpezo
Aumar Dadjo
Dénis Fanou

Hôpital Evangélique de Bembèrèkè
Ethienne Yado*
Timothé Gouroubéra

Hôpital de zone Sakété
Michel Noukounwoui*

Centre Hospitalier Universitaire Departemental Borgou Alibori
Blaise Tchaou*
Charles Tchegnonsi
Fifame Amadji
Francine Bossa

Hôpital D'instruction Des Armées De Cotonou
Fanou Lionelle*

Nounagnon Gilbert

Hôpital de Zone de Banikoara
Gilberte Hounkpe*
Bachabi Fafana
Néné Nguilu

Hôpital De Zone De Mènontin
Evelyne Gnele-Dedewanou*

CHD-Atacora
Oswald Gbehade*
Romaric Tobome
Adam Boukari
Patrick Bakantieba
Arouna Sambo

Centre Hospitalier Universitaire de Zone Suru-Léré
Hugues Chobli*
Elie Fassinou
Aurore Zoglobossou

HZ Malanville
Valéry Adjignon*

Centre Hospitalier Universitaire Départemental de l'Ouémé et du Plateau
Ernest Ahounou*
Djima Alao
Roushdane Odérémi
Afissatou Montairou

Centre National Hospitalier et Universitaire Hubert Koutoukou MAGA
Serge Mewanou*
Aïcha Tchomgang
Urielle Agossou
Fernand Soton
Charbel Azanlin

CHU-MEL Cotonou / CHU de la Mere et de L'enfant Lagune
Eugene Zoumenou*
Lidwine Zomahoun

Burkina Faso. National Leader: Nazinigouba OUEDRAOGO
Centre hospitalier universitaire de Tengandogo
Rawéléguinbasba Armel Flavien Kabore*
Salam Savadogo
Fatou Fleur Rosine Sanou
Farid Belém
Victoria Hien
Cheik Tidiane Hafid W Bougouma
Sie Ahmed Ouattara

Hôpital de District de Nomgr-Massom
Mariam Bambara Kabore*

Centre Hospitalier Universitaire Yalgado Ouedraogo
Ouedraogo Nazinigouba*
Papougnezambo Bonkoungou*
Martin Lankoandé*
Mireille Traoré*
Patrick Sawadogo*
Inès Wenmenga*
Boureima Kinda
André Simporé

*Hôpital de district de Boulmiougo*u
Christian Sapo*

Centre Hospitalier Universitaire de Bogodogo

Salah Idriss Traore*
Haoua Dipama
Lydie WR Kaboré
Salifou Napon
Télesphore G Kaboré
Arouna Louré
Pélagie PP Tondé
Christian Zoundi

Centre Hospitalier Régional de Koudougou
Martin Lankoande*
Harouna Sanou

Burundi. National Leader: Venerand Barendegere
Kibuye Hope Hospital
Gregory Sund*
Alliance Niyukuri

Kamenge Military Hospital
Remy Ndikumana*
Venerand Barendegere
Carlos Nsengiyumva

Centre Hospitalo-Universitaire de Kamenge
Axel Kwizera*
Jean-Claude Niyondiko*

Democratic Republic of the Congo. National Leader: Dolly Munlemvo
Cliniques Universitaires de Kinshasa
Adolphe Manzanza Kilembe*
Jean Pierre Mwema Ilunga
Nehema Hailemariam Sarah
Gabriel Mubobo Makeya

El Rapha Clinic
Idesbald Mwebe Mwepu*
Ted Botawaosenge Likongo

Cliniques Ngaliema
Richard Kapela Mvwala*
Raphael Nzau Kapend Mubunda
Noellie Kanka Mukuna

Hopital General de Reference de Kinshasha
Julie Djondo Pembe*
Nicolas Lumuanga Ndaye

Centre Nganda
Eric Bibonge Amisi*
Mike Ilunga Madika

Médecins de Nuit
Joe Kembo Lungela*
Didier Ndonda Mayemba

Hopital Sino Congolais
Philomene Mamba Diyoyo
Alex Mbo Ngalala*

Hopital Diamant
Martin Mamba Mukenga*

Hopital General de Kintambo
Patricia Tito Kabuni
Dany Bolimo Mpoto*
Herve Inesnku Mole
Louise Keby
Oria Andavo Buti

Centre Hospitalier du Mont Amba
Anselme Phaka*
Belinda Mayenge

Hopital Biamba Marie Mutombo
Jean Jeacques Kabuley Kalongo*

Hopital General de Ngiri-Ngiri
Timothe Kemfuni Mawisa*
Rodrigue Tondo Ngwizani

Centre Hospitalier Akram / Pont Matete
Kuyala Leya*

Matter Dei
Dieudonne Kisile Sanduku*
Timothe Nkemfuni Mawisa

Hopital Roi Bodoin Ler
Coco Nseke Mfumu*
Mbuta Bolenge

La Borne
Desire Kinzenzengu Kabuce*
Patrick Kintieti

Centre Hospitalier d'Etat de Matete
Amelia Mbuluku*

Hôpital d'Etat de Makala
Vicky Mahuwa*
Tharcice Khonde Mabiala

Hôpital 12e Rue Limete Petit Blvd Indus / Hôpital Voici l'Homme
Guilain Ngoy*

Mere et Enfants Barumbu
Patrick Boloko
Nono Mazangama Mvwama*
Jose Kengbanda

HGR Masimanimba
Pitchou Mushimbonga*
Blaise Kuhapala

HGR De Kikwit
Nzosani Marcel*
Kienze Guylain*

HGR Mosango
Gerard Mboma*

Cliniques Universitaires de Lubumbashi
Sandra Zalambo Sagboze
Michel Muteya Manika*

Heal Africa Goma
Jean Pierre Mumbere Kigayi*

CH Biopharma BKV
Roger Mukanire Cishugi*

HGR Nyantende
Placide Buhendwa Mugisho*

HGR Panzi
Roger Baguma*

Hôpital Dipumba

Moïse Kongolo
Michel Mandungu Mbayabu*

Hôpital Bonzola
Crispin Mukendi Muamba*
Edmond Banema Kapinga
Vasco Ngolela Kapinga

Hôpital Presbytérien de Mbujimayi
Guylain Tshimanga Nsumpi*

Polyclinique de Mbujimayi
Patrick Kanda Odia*

Hopital Ime/Kimpese
Salomon Bingidimi*
Gilbert Kpengbemale
Desire Hubert Bofunga Bosonga Imposo

HGR Kinkanda
Patricia Matondo*
Servet Lelo
Jeremie Kalambayi

Grace a Dieu
Patrick Kanda Odia*

Egypt. National Leaders: Mahmoud Elfiky and Maher Fawzy
Cairo University Hospitals
Maher Fawzy*
Mahmoud Elfiky

Suez Canal University Hospitals
Mohamed Abdel-Ghaffar*
Abdelrahman Soliman
Mostafa Abdelrahman

Alexandria University / El Shatby University Hospital
Sameh Shehata*
Alia Rabee
Mohamed Abou Heba
Mohamed Rabei Abdelfattah
Tamer Ahmed Maher Ghoniem

Tanta University
Sherif M.K. Shehata*
Mohamed Lotfy
Ahmed ElHaddad

Ethiopia. National Leaders: DZ Ashebir, Mahlet Tesfaye Bahta, Hassen Mohammed, Mikiyas Teferi, Yakob Seman
Dil Chora Referral Hospital
Rahel Assefa*
Abdurahman Gelmo
Seifu Alemu
Brook Damtachew
Merid Mersha
Yaekob Chemere
Samuel Fekadu
Sintayehu Regasa
Bonsa Sileshi
Desalegn Wosen
Rebira Adamu

Maddawalabu University Goba Referral Hospital
Zewude Gudisa*
Kebebe Bekele*
Alelign Tasew
Habtamu Gezahegn

Daniel Atlaw
Damtew Solomon
Habtemariam Gebresillasie
Girmaye Tesfaye
Negussie Sarbecha
Biniyam Sahiledengle
Kenbon Seyoum
Gemechu Ganfure
Yohannes Tekalegn

Jimma University Institute of Health
Gersam Mulugeta*
Teshome Bacha

Yirgalem General Hospital
Musefa Redwan*
Eyasu Muse
Girma Nina
Bizuneh Sime
Addisalem Tadege
Anbesse Jima

Dessie Referral Hospital
Fassil Mihretu*
Yesuf Ahmed
Bekele Debebe
Abdureuf Misgea
Amare Agmas

Debre Birhan Comprehensive Specialized Hospital
Fitsum Kifle*
Kokeb Desita
Abebe Addise
Dagim Shimelash
Shitalem Tadesse
Bezaye Zemedkun
Peniel Kenna

Wolaitta Sodo University Teaching and Referral Hospital
Temesgen Ayichew*
Shibiru Sendaba

Tibebe Ghion Specialized Hospital
Gosa Tesfaye*

Ambo University Referral Hospital
Desalegn Abdisa*
Abebe Megersa
Wendwosen Abayne

Ayder Comprehensive Specialized Hospital
Haftom Berhane*
Reiye Esayas

Alert Hospital
Abdurezak Ali*
Kindie Moges
Jemal Ahmed

Debre Markos Referral Hospital
Ayenew Yirdie*
Abinet Sisay
Tebikew Gashu

Aksum University Comprehensive Specialized Hospital
Bereket Gebremeskel*
Girmay Fisseha
Mebrahtu Abay
Degena Bahrey

Assefa Hika

Menelik II
Nugusu Ayalew*
Dagmawi Workneh
Daniel Teferi

Gambia. National Leader: Mustapha Bittaye, Ahmadou Lamin Samateh
Kanifing General Hospital
Alagie Manneh*

Bwiam General Hospital
Kitabu Jammeh*
Abdoulie Keita*

Farafenni General Hospital
Ebrima Kanteh*
Sarjo Ceesay

Bansang Hospital
Momodou Nyassi*

Bundung Maternal Child Health Hospital
Landing N Sanyang*
Omar Jallow

Edward Francis Small Teaching Hospital
Mustapha Bittaye
Ahmadou Lamin Samateh
Charles Roberts*
Patrick Idoko
Kebba Marenah
Masirending Njie
Musa Marena
Karamba Suwareh
Simon Boissey
Fatoumatta Jarjusey
Awa Jah
Awa Sanyang
Dado Jabbi
Kajali Camara
Armando Correa
John Jabang
Lamin Jaiteh
Lamin Dampha
Aminata Manneh
Abdoulie Keita
Baboucarr Sowe
Abdoulie Bah
Edrisa Jawo
Victoria Okoje
Momodou Baro
Yaya Bah
Mustapha Njie
Sainabou Mbowe

Ghana. National Leader: Akwasi Antwi-Kusi
Greater Accra Regional Hospital
Evans Atito-Narh*
Adwoa Wilson

Holy Family Hospital, Berekum
Romeo Hussey*
Emmanuel Okine
Jemima Kwarteng
Ridge Ntiamoah
Samuel Dadzie
Mark Aseti

Naa Sowah
Akosua Appiah
Charles Bankah

Kenya. National Leader: Zipporah Ngumi
Kenyatta National Hospital
Zipporah Ngumi
Patrick Mburugu*
Thomas Chokwe
Patrick Olang
Vernon Gacii
Susane Nabulindo
Antony Gatheru
Timothy Mwiti
Caroline Mwangi
Julius Muriithi
Daniel Ojuka
Omondi Ogutu
Evans Masitara
Mohamed Chaudhry
Reuben Kamundi
Annmarie Kangangi
Thomas Massaquoi
Stephen Takow
Felister Moraa
Aziz Munubi

Kiambu Level V Hospital
Moses Kimani*
Adili Wobenjo
Linda Nguu

Kisii Teaching and Referral Hospital
Vincent Omeddo*
Rose Malaba
Ambrose Nabwana
Anita Mwancha
Alexandria Mugaa

Mater Misericordiae Hospital
John Wamwaki
Joyce Chege
Seymour Sinari
Andrew Ndonga*

Moi Teaching and Referral Hospital
Rose Shitsinzi*
Walter Akello
Winfred Kimani
Elisha Kirwa
Seno Saruni
Andrew Wainaina
Ernest Nshom
Aidah Kenseko

Naivasha District Hospital
Kizito Shisanya*
Purity Wanjiru
Julliah Cherotich
Judy Kimutai
Benson Wahome
Grace Wangui
Dennis Wamalwa
Stephen Mwangi
John Chege
Tabitha Wanjiku

Nakuru Level 5 Hospital
Carolyne Njoki*

Wachira Waititu
Conrad Ambani
Samuel Murimi
Sharon Waithira
Nilson Mouti
Collins Kibet
John Kibet
Virginia Sokobe
Beatrice Jeymah
Antony Kamadi
Faith Gichuri
Steve Moses
David Wasike
Favours Adeya

JOOTRH Level 6 Hospital
Caesar Bitta*
Stephen Ogendo*

Muranga County Hospital
Killian Kariuki*

Libya. National Leaders: Muhammed Elhadi, Mohamed Elfagieh
Benghazi Medical Center
Taha Abubaker*
Fatima Elkhfeefi
Soha Younis Hasan
Amal Alttaira
Imbarkah Elmraied
Rim Wishah

Tobruk Medical Center
Ahmad Bouhuwaish*
Munyah Alriqeeq
Mabruka Omar
Ashraf Samer
Aml Aemeesh

National Heart Institute
Bushray Almiqlash*
Abdulsalam Alshuhoumi
Rayet Al-islam Ben Jouira
Amaal Dier
Essra Gebril

Mietiga Hospital
Mohammed Albashri*
Marwa Morgom
Mohammed Alawami
Eman Shawesh
Abdullah Almabrouk
Moaz Alwarfalli
Nagia Abeid
Anis Buzreg
Ans Malek
Ameerah Abayu
Butaina Abdulhafith

Medical Care Clinic
Malek Abudsnnuga*
Salsabil Albuaishi

Sabha Medical Center
Mohammed Abdelkabir*
Sana Moussa
Marwa Alfitori
Mabroukah Azbeda
Hajar Alamin
Ejmeya Barka

Omlsaad Mosbah
Rema Abdasalam
Miftah Hiyoum
Maryam Abd allateef
Ibrahim Altomi
Mahdi Alsakloul
Ekhlas Karami

Aljalla University Hospital Benghazi
Khaled Elgazwi*
Adel Elgazwi*
Ibrahim El-busife
Safa Owhida
Almahdi Eltwati
Samah Elakeili

AboSleem Trauma Hospital
Hdaya BenAbdalla*
Taha Suliaman
Fatima Ali Abokhzam
Mohaned Isa
Mohammed Huwaysh
Asma Bourawi

Tripoli Central Hospital
Ahmed Msherghi*
Ahmed Elusta
Sultan Ahmeed
Wesal AlFighi Hassan
Mouadah Ali Altayr
Mohamed Addalla
Abrar Geddeda
Kheria Khoja
Muaad Etturki

Tripoli University Hospital
Ahmed Elhadi*
Ibrahim Ellojli
Anshirah Shuwayyah
Ahmed Elfaghih
Malak Alduwayb

Aljalla Maternity Hospital
Ahmed Msherghi*
Amira Mohamed
Ala Khaled
Nouran Aljadi
Elham Bareig

Zliten Hospital
Mohamed Aleiyan*
Wedad Aboubreeq
Soliman Alkassem
Sami Ashour
Hayat Ben Hasan
Najat Ben Hasan
Ali Yahya

Gheryan Hospital
Akram Alkaseek*
Hoda Elhaddad
Ayyah Alqaarh
Ahlam Brish

Elkhadra Hospital
Omar Abugassa*
Hazem Ahmed
Amera Ellafi
Shoukrie Shoukrie
Nawal Aldokali

Aws ElGammudi

Al Hawari General Hospital
Kais Alzubaidy*
Mohammed Albaraesi

Mislata Hospital
Wedad Abouruwes
Marim Albakuri*
Sabria Almuammari

Misrata Medical center
Esam Alsaghair*
Fatheia Alreshi
Hassan Badi
Rabiee Alfetoey

National Cancer Institute
Mohamed Elfagieh
Naji Zubia*

Al-Zawia Teaching Hospital
Sumayyah Bahroun*
Abdulmueti Alhadi

Malawi. National Leader: Wakisa Mulwafu, Vanessa Msosa
Queen Elizabeth Central Hospital
Wakisa Mulwafu*
Palesa Chisala*
Edward Kommwa
Lusayo Simwinga
Agness Chalira

Kamuzu Central Hospital
Vanessa Msosa
Precious Kachitsa
Onias Mtalimanja

Mali. National Leaders: Zimogo Sanogo, Youssouf Coulibaly
Chu Gabriel Toure
Adégné Togo*
Bakary Dembele
Djibo Diango
Moussa Samake
Youssouf Traore
Louis Traore

Centre Hospitalier Universitaire Du Point G
Zimogo Sanogo
Youssouf Coulibaly
Drissa Traoré*
Moussa Sissoko*
Moussa Camara*
Adama Koita
Sekou Koumaré
Omar Sacko
Mahamadou Coulibaly
Lamine Soumaré
Soumaïla Keita
Sidiki Keita
Hamadoun Dicko
Boubacar Diallo
Boureima Bengaly
Mohamed Keita
Siaka Diallo
Drissa Ouattara
Nouhoun Ongoiba
Seydina Beye

Honoré Berthe
Mamadou Diakite
Mamadou Sima

Hôpital de Tombouctou
Ongoïba Oumar*

Hôpital Fousseyni Daou de Kayes
Sogoba Gaoussou*
Issaga Traoré
Sidy Sangaré
Doua Kanté
Lassana Cissé

Hôpital Hangadoumbo Moulaye Touré De Gao
Thiam Souleymane*

Hôpital Hangadoumbo Moulaye Touré De Ga
Keita Koniba*

Mauritius. National Leader: Veekash Gobin
Flacq hospital
Divyanand Jankee
Ashveen Puryag
Meetheelesh Abeeluck*
Senthil Beemadoo
Yashraj Deenoo
Abhisek Goureah
Munawwara Makoon
Hemanshu Rambojan
Varun Seewoo*
Beeharry Shanjugsingh
Kevin Viraswami

Jawaharlal Nehru Hospital
Ashwant Bhugwandass
Shehzaad Joomye*
Bibi Deelawar

Sir Seewoosagar Ramgoolam National Hospital
Sajid Aungraheeta
Vakil Leelodharry*
Sirsingh Bhajoo
Luckshmanraj Mungur*

Wellkin Hospital
Gini Batra
Subha Gaya*
Yoshvin Sunnassee
Shailendra Petkar

Clinic Muller
Sundaresan Maiyalagan*
Julien Chong
Adil Mohit
Khushyant Mungar
Kevin Ramlochun
Jamie Sim
Desai Sneha
Shankaran Vinayagam

Victoria Hospital
Manpreet Rajcoomar*
Rishi Seetaram

Dr AG Jeetoo Hospital
Vishaal Kissoon
Yan Tseung
Mohamed Aboobakar

Nitish Fokeerah*
Ravi Ramsewak
Jayprakash Gopall

Morocco. National Leader: Mohyeddine Zarouf and Ahmed Rhassane EL ADIB
Hôpital Mohammed VI / CHU Tanger
Hicham Sbai*
Labib Smail
Ait Laalim Said
Motaai Youssef

Hôpital des spécialités CHU Rabat
Mouhssine Doumiri*

Hôpital Ibn Sina CHU Rabat
Mustapha Alilou*
Nora Farnaoui

Hôpital militaire d'instruction Rabat
Mustapha Bensghir*
Abdelghafour Elkoundi
Abdelhamid Jaafari
Abderhmann Elwali
Mohammed Meziane
Walid Atmani

CHU Marrakech - Hôpital Mére-Enfant, Centre Hospitalier Universitaire Mohammed VI Marrakech
Ahmed Rhassane El Adib
Houssam Rebahi
Hajar Chichou
Safae Zarouf
Abderraouf Soummani

CHU Marrakech - Hopital Arrazi, CHU Mohammed VI
Aboulhassan Taoufik*
Meryem Essafti
Aminata Oumou Traore
Hamzaoui Hamza

CHU Fes
Adnane Berdai*

Mozambique. National Leader: Atilio Morais
Hospital Central Da Beira
Nelson Mucopo*

Hospital Central de Maputo, Faculdade de Medicina, Universidade Eduardo Mondlane
Atilio Morais*
Machado Banze
Mouzinho Saide
Tomas Sitoi
Artur Machava

Pemba Provincial Hospital
Antonio Carlos*
Amilton Guidione
Antonio Saide de Carvalho

Hospital Geral de Mavalane
Natacha Gemo*

Hospital Provincial de Xai-xai
Samiro Sema Camal*

Hospital Provincial de Chimoio
Arsénio Cuna*

Hospital Central de Quelimane

Ornelos Madeira*
Ladino Assuade

Hospital Central de Nampula
Dercio Amde Fernandes*
Dulce Alexandre Machavae Fernandes

Hospital Provincial de Lichinga
Mandua Sebastião*

Namibia. National Leader: Apollo Basenero
Otjiwarongo State Hospital
Paidamoyo Mandudzo*
Sandra Kapepiso

Walvis Bay District Hospital
Martha Ntinda*

Outapi Hospital
Haziel Mavesere*

Intermediate Hospital Oshakati
Ruben Nailonga*
Immanuel Uukonga
Uutoni Nakanyala
Lavinia Johannes
Adrian Haruzuvi
Chris Terblanche
Natangwe Shimenda
DelwinaKatjipu
Hilma Shalimba

Opuwo District Hospital
Hilma Katangolo*

Intermediate Hospital Katutura
Ike Ndjoze*

Khorixas District Hospital
Juliah Kaweendwa*

Onandjokwe State Hospital
Mutombo Ndaie
Peter Njuki*
Akutu Munyika

Windhoek Central Hospital
Pueya Nashidengo*
Johanna Kandjumbwa
Lahia Lipumbu

Engela District Hospital
Matti Kandjimi
Bernard Sikombe*

Mariental State Hospital
John Oyedele*
Mapumba Mulolo*

Luderitz District Hospital
Ali Mbuyi*

Outjo District Hospital
Cedia Tjihoto*

Tsumeb District Hospital
Beata Siteketa*
Beata Kaholongo

Oshikuku Hospital
David Tjiyokola*
Tawanda Mhene
Archbald Masiambiri

Gobabis State Hospital
Ayoub Shekimweri*
Diana Shilomboleni
Mbaundju Kandjii
Leonard Kabongo
Cholastic Hangero

St Mary's Hospital Rehoboth
Daylight Manyere*
Charlotte Kauraisa
Learnmore Garanowako
Michael Tune
WilliamDeKlerk
Benvenue Ndolo
Maria Angula
Ndapewoshali Hishekwa
Elizabeth Nandjendja
Elsabe Tsauses
EuniceMouton
Kudzai Katandawa
Sophia Bruwer
Jaydee Van Staden

Niger. National Leader: Maman Sani CHAIBOU
Centre De Sante De La Mere Et De L'enfant Diffa
Moussa Sirfi*

Centre Hospitalier Regional, Agadez
Fouma Djibo*

Centre Hospitalier Régional de Diffa
Moutari Mahaman*

Centre Hospitalier Régional de Maradi
Adakal Ousseini*
Maikassoua Mamane
Abdoulaye Mahaman Bachir

Centre Hospitalier régional de Tahoua
Maikassoua Mamane
Maman Noury Hamissou Souley

CHR Poudriere / Centre hospitalier regional de Niamey
Rekia Idrissa*
Moussa Ichaou
Amina Saley
Abdoulay Seyni
Sahabi Amadou

District sanitaire de Konni / Hopital de District de konni
Mahamane Sani Mahamane Laminou*
Issoufou Moustapha Camara*

Hôpital National de Niamey
Moussa Gagara
Hadjara Rabiou Daddy

Hopital National de Zinder
Harissou Adamou*
Ibrahim Amadou Magagi
Oumarou Habou

Hopital National Lamorde de Niamey
Sabo Ramatou

Nigeria. National Leader: Akinyinka Omigbodun, Simbo Amanor-Boadu †,

Lagos University Teaching Hospital
Olufemi Bankole*
Bosede Afolabi
Dapo Osinowo
Ayodeji Oluwole
Muyiwa Rotimi
Ibironke Desalu
Rufus Ojewola
Bolaji Mofikoya

National Hospital Abuja
Jamiu Adebiyi
Temitope Babalola
Mansur Muhammad
Afeez Aruna
Maryrose Osazuwa
Muhammad Mahmud*
Ayodeji Yusuf
Isiaka Lawal

University College Hospital Ibadan
Tinuola Adigun
Babatunde Osinaike
Olayinka Eyelade*
Akinyinka Omigbodun
Olayinka Ogunbode
Olusola Idowu
Taiwo Lawal
Temidayo Ogundiran
Olayiwola Shittu
Omobolaji Ayandipo

University of Abuja Teaching Hospital
Olumide Akitoye
Bissallah Ekele
Godwin Akaba
Stephen Edino*
Terkaa Atim
Zumnan Songden*
Akitoye Adeleke
Owoicho Okochi
Sunday Akeju
Ernest Ukpoju

Abubakar Tafawa Balewa University Teaching Hospital
Saidu Kadas*
Rabiu Mohammed*
Abubakar Ballah
Tella Olalekan
Kefas Bwala
Mohammed Adamu
Adamu Isa

Ahmadu Bello University Teaching Hospital
Euphemia Ugwu
George Mukoro
Muhammed Ahmed
Gideon Akafa
Ahmad Lawal
Daniel Nwoye
Michael Odigbo
Zulaihatu Sarkin-Pawa
Tunde Sholadoye
Benjamin Fomete
Hamisu Yakubu
Abdulkadir Kabiru
Samaila Timothy

Ali Yusuf
Rabiu Mohammed
Tasiu Saadu
Babangida Mohammed
Abdulghaffar Yunus
Ganiyat Olagunju
Muhammad Aminu
Mohammad Idris
Elizabeth Nwasor*
Musliu Tolani
Saidu Yakubu*
Nasiru Dalhat
Samuel Gana
Talent Adike

Babcock University Teaching Hospital
Benjamin Nkemjika
Omotayo Salami*
Adekunle Akadri
Bukola Olayinka
Clement Onuoha

Federal Medical Centre, Azare
Umar Usman Jamaare*
Auwalu Saminu Jibrin
Sani Giade Abdullahi
Ibrahim Ishaku

Federal Medical Centre, Jabi, Abuja
Amechi Ezike*
Olatunde Olawoye
Emmanuel Ugwu
Christopher Ukah
Abolade Olugbenga
Nwachukwu Chidiebere
Nasiru Abdulraman
Adebiyi Olusegun
Alisa Halisa
Semiat Yusuf
Jamila Salisu
Chidiebube Okoro
Abdul Suleiman
Fabian Onowighose

Federal Medical Centre, Lokoja
Olatunde Alabi*
William Adeyemi
Jelili Salau
Jones Taiwo
Nnaemeka Nwafulume
Taiye Ibiyeye
Edith Agu
Ayodeji Danboy
James Abdulazeez

University of Benin Teaching Hospital, Edo State
Osayomwanbo Osaheni*
Ifunanya Obaseki
Lateef Kehinde
Osawemwenze Monday
Stanley Nte
Adesuwa Agboifo
Omajuwa Dawodu
Precious Orhiere
David Atiti

University of Ilorin Teaching Hospital
Israel Kolawole*
Olawale Ojo
Afusat Olabinjo

Olufemi Ige
Beatrice Ogunyemi
Olusola Oladosu
Kikelomo Adesina
Sulaiman Agodirin
Asimiyu Shittu

Federal Medical Centre, Yola
Abdulrahman Mohammed*

Afe Babalola University Multi-speciality Hospital, Ado Ekiti
Samuel Fayose
Ademola Adeyeye*
Akinola Akinmade
Taiwo Ajayi

Ladoke Akintola University Teaching Hospital, Ogbomoso
Adeolu Adeoye
Temidayo Bobo
Kola Owonikoko*
Taiwo Akinloye

University of Maiduguri Teaching Hospital
Audu Idrisa*
Sadiq Adamu
Ahmed Nuhu
Nuhu Ali
Olayinka Adewunmi
Stephen Nwankwor

University of Port Harcourt Teaching Hospital
Olubusola Alagbe-Briggs*
Amabra Dodiyi-Manuel
Bisola Onajin-Obembe
Bright Obasuyi
Richard Echem
Ihuoma Mike-Elechi
Job Otokwala

Nigerian Navy Reference Hospital, Calabar
Abdullateef Abdulazeez*
Chabiya Bala
Eziamaka Eze
Elizabeth Ani
Uchenna Okeke
Dominica Adebayo

Jos University Teaching Hospital
Benjamin Ugwu*
Samuel Nuhu*
Henry Embu*
Erdoo Isamade
Chinedu Obikili
Amaka Ocheke
Solomon Peter
Donald Orshio
Peter Onuminya
Jack Okopi

Aminu Kano Teaching Hospital
Lofty-John Anyanwu*
Abdurrahman Sheshe
Sani Aji
Mamuda Atiku
Raphael Attah
Abubakar Muhammed
Rasaki Oseni
Halima Salisu-Kabara

University of Uyo Teaching Hospital

Mark Edubio*
Catherine Eyo
Isaac Udo*

Federal Teaching Hospital, Ido Ekiti
Adebayo Adeniyi*
Toluwalope Ariyo
Olajide Gabriel
Tesleem Orewole*
Salawu Idris
Idowu Adebara
Abiodun Okunlola
Akinwale Akinbade
Oluwasesan Afolabi
Adewumi Bakare
Olabisi Adeyemo

Federal Medical Centre, Umuahia
Nnabuike Ojiegbe*
Charles Mbamba
Paul Ngwu
Frank Imahigbe
Emmanuel Okoroji
Iloh Ikenna

Yariman Bakura Specialist Hospital, Gusau
Abidemi Oyaromade*
Mohammed Bello
Usman Muhammad
Emeri Mbah
Hyacinth Okereke
Almustapha Aminu
Anthony Ahmadu

State Specialist Hospital Maiduguri Borno state
Nankat Joseph*
Salihu Bura
Chukwuka Nwezoku
Manu Bwala
Meshach Philips
Abubakar Usman
Emmanuel Filibus
Zara Umate
Nwabuoke Chukwuka
Ahmed Nuhu
Watakiri Ibrahim
James Nggada
Ali Izge
Musa Ismail

Obafemi Awolowo University Teaching Hospitals Complex, Ile-Ife
Simeon Olateju*
Fred Ige-Orhionkpaibima
Adedapo Adetoye
Olurotimi Aaron
Jeremiah Abimbola
Folayemi Faponle
Olumuyiwa Ajayeoba

University of Medical Sciences Teaching Hospital Complex, Ondo
Akinwumi Olakanmi*
Oluseye Ajayi
Ayotolu Ajayi
Victor Ogunmola
Oluwafunke Olakanmi
Adam Kuranga

Federal Medical Centre, Gusau
Yetunde Aremu-Kasumu*
Kamil Shoretire*

Peter Enesi
Jimoh Buraimoh
Kabiru Muhammad
Sophia Baidoo
Patrick Okoli
Azeez Adigun
Ekene Ezeonye
Kabir Isa

Federal Medical Centre, Katsina
Aliyu Farinyaro*
Suleiman Baba
Umar Abdulmajid
Aisha Abdurrahman
Ogochukwu Obi

Mater Misericordiae Hospital, Afikpo, Ebonyi State
David Ama*
Ebere Okoronkwo

New Boshang Hospital Ltd
Bitrus Fidelis*
Rephath Pius
Cyril Jomosu

University of Nigeria Teaching Hospital, Enugu
Enoch Uche*
Chukwuemeka Osuagwu
Chukwudi Ilo
Mesi Matthew
Uko Uko
Ngozi Mba

Olabisi Onabanjo University Teaching Hospital
Olusoji Jagun*
Oluwabunmi Fatungase*
Adeniyi Akiseku
Chigbundu Nwokoro
Ramotalai Shoyemi
Ibukunolu Ogundele

University of Calabar Cross River State
Queeneth Kalu*
Felix Effiom

Nigerian Navy Reference Hospital Ojo, Lagos
Adebayo Adedayo*
Abubakar Aliyu
Adaora Agholor

Nnamdi Azikiwe University Teaching Hospital
Okechukwu Ekwunife*
Victor Modekwe
Chuka Ugwunne
Chukwuemeka Okoro
Chisom Uche

Federal Medical Centre, Abeokuta
Adenike Odewabi*
John Bamigboye
Oladapo Kuforiji
Chidiebere Ogo
Stella Ogunmuyiwa
Abdussemee Abdurrazzaaq

Federal Medical Centre, Owerri
Christopher Ekwunife*
Chimaobi Nnaji
Chigozirim Onyekpere
Amara Arunsi

Jude Egwim
Obianuju Nwana

Federal Medical Centre, Ebute Metta, Lagos
Adebayo Tanimola*
Michael Adeyanju
Oluwatimilehin Andero
Temitope Ojo
Olusi Adedotun
Gbadamosi Kehinde

Usmanu Danfodiyo University Teaching Hospital
Aliyu Abdulrahman*
A.A. Abdullahi
Ibrahim Galadima Bello
Usman Adinoyin Mohammed

Sierra Leone. National Leader: Isaac Smalle
Bo Government Hospital
Abu Rogers*
Peter Samai
Sao Amara
Margaret Yankuba
Mary Josayah
Jayah Swarray Jnr

Connaught Hospital
Alusine Dawo*
Peter George*†
Isaac Smalle
Mustapha Kabba
Mohamed Bah
Charles Mondeh

Kenema Government Hospital
Ibrahim Kapuwa*

Makeni Government Hospital
Philip Mattia
Brima Sesay
Mohamed Sheku*

South Africa. National Leader: Hyla Kluyts
Provincial leaders:
Gauteng South: Jones O.A. Omoshoro-Jones
Gauteng North: Motselisi Mbeki
Western Cape: Estie Cloete
Northern Cape: Philip Anderson.
KwaZulu Natal: Alexandra Torborg
Eastern Cape: Busi Mrara
Northwest: Annemarie Steyn.
Limpopo: Tsakani Mhlari.
Mpumalanga:. Nic Proctor, Caroline Robertson
Free State: Gillian Lamacraft.

Addington Hospital
Sebenzile Sikhakhane
Usha Singh*

Bethesda Hospital
Kelly Gate*

Charles Johnson Memorial Hospital
Shepherd Nzenza*

Estcourt Provincial Hospital
Patrick Lufuta Kande*

Eshowe District Hospital
John Tshimbalanga Kasonga*
Sibongile Ndebele

George Regional Hospital
Tino-vito Orlandi
Marischka de Jong
Hugo Stark
Francois Roodt
Jody Davids*
Jonathan Hall
Ian Nortje
Akanimo Akpakan

Khayelitsha District Hospital
Muneerah Cassiem
Sarwat Ikram-Hameed*
Adriaan Smit

King Dinuzulu Hospital
Yvonne Freeman*
Saaliha Goga*

King Edward VIII Hospital
Larissa Cronje*
Suman Mewa Kinoo
Vivesh Rughubar
Rishan Bipath
Constantin Buzdugan
Subash Chirkut
Priyadeshni Singh
John Arnold
Ncumisa Msolo
Sandhaya Jithoo

Mahatma Gandhi Memorial Hospital
Tshegofatso Mmasello Emma Boka
Martyn Biccard Greenwood
Shakthi Anand Jayrajh
Devarani Naidoo
Syndrini Reddy*
Devandiran Harriraman Rungan*
Kylene Subrayen

Manguzi Hospital
Fleur Ackermans-Deijnen*

Mitchell's Plain District Hospital
John Roos*
Nina Tredoux

Mosvold Hospital
Pascal Simon Plumacher*

New Somerset Hospital
Estie Cloete
Paul Ryan Herselman
Mariesa Nock
Anthony Reed*
Harald Steinhaus
Nicola Vickery

Paarl Hospital
Gareth Davies*
Talitha Harvey
Franklin Muller
Willem Naude
Tania Pretorius
Johan Jochemus Swart
Merryn Walls

Prince Mshiyeni Memorial Hospital
Prashant Gokal*

RK Khan Hospital
Farzana Dhoodhat
Precious Dzanibe
Mohammed Hussain
Asmitha Junpath
Ameela Maharaj
Hylda Makanisi
Khalid Moosa
Nicolette Rorke*
Ting Ting Wong
Sean Mould

St Aidan's Hospital
Trisha Ramsamy*

Stanger Hospital
Vasheel Bahadur
Roel Matos-Puig*
Hayley Morgan
Nadeem Nabeebuccas
Ria Devi Naidoo
Viantha Pather

Victoria Hospital
Nicole Bell
Zahnne Fullerton*
Bongisa Grey
Vincent Lorenzo Visentin

Worcester Regional Hospital
Terri Anne Killingbeck
Emile Maneveldt
Oostewalt Swart
Gerhard Thiart
Hendrick Adriaan van Zyl*
Magdelena May Venter

Grey's Hospital
Ronisha Sathiram
Mohammad Kathrada
Vishendran Govindasamy*
Zane Farina
Lucio Frittella
Charles Kohler
Sibuyiselwe Lubelwana

Tygerberg Hospital
Renilda Pillay*

Edendale Hospital
Mariette Grobelaar*
Carel Cairns*
David Bishop
Christien Steenkamp
Thandekile Khumalo

Port Shepstone Hospital
Noel Naidoo*

Citrusdal Hospital
Ross Murray*

Madadeni Hospital
Martin Kopieniak
Mxolisi Brian Ndimande

Melusi Sishange

Karl Bremer Hospital
Megan Jaworksa*

Helderberg Hospital
Megan Jaworksa*

Eerste River Hospital
Sarwat Ikram*
Bence Rainier

Beaufort West Hospital
Renier J Liebenberg*
Helena D Zwiegers
Philip M Nortje

Groote Schuur Hospital
Kamal Bhagwan*
Estie Cloete
Margot Flint
Robert Dyer
Simone Adams

Queen Nandi Regional Hospital
Yoshua Bwambale*
Danny Ngomo
Patrice Kanku

Inkosi Albert Luthuli Central Hospital
Nivashen Pillay*
Alexa de Castro
Atisha Maharaj
Janine Carim
Jenna Leigh Taylor
Karl M Köhne
Leanne W Drummond
Leanne Temlett
Lieze Geldenhuys
Lucy Rolt
Yvonne Seilbea
Kathryn Naidoo
Nicola A Kalafatis
Stefné Verwey
Thulile Biyase
Theroshnie Kisten
Belinda S Kusel
Timothy Craig Hardcastle
Richard Magagula
Christian Kampik
Kuzolunga Xulu
Sivuyisiwe Solala
Mia Sayed
Basil Enicker
Anil Madaree
Innocent Mukama
Alexandra Torborg

Ladysmith Regional Hospital
Gladmore Madombwe*
Nonhlanhla Zulu*
Nompumelelo Gasa
Nokuzula Kanjana
Sebenzile Buthelezi
Thembelihle Buthelezi

Knysna Provincial Hospital
Andries Brink*
Francois Potgieter

Nelson Mandela Academic Hospital
Busisiwe Mrara*
Freddy Kabambi
Zaynab Alexander
Charles Choto
Paula Ima
Zintle Gxagxisa
Baphethuxolo Ningiza

Universitas Academic Hospital
Gillian Lamacraft*
Jerome Mogorosi
Nadia du Plessis
Leonie de Man
Suné Thompson
Gerrit van Heerden

Pelonomi Hospital
Edwin W Turton*
Pieter M van der Linde*
Josephine K Teme-Pitse
Reitumetse Tladi
Gillian D Saffy
Ene-Mari Roscher
Kristel Fortune
George Barnard
Tiisetso Makhasane
Evan Bowen
Akangcha Pal

Chris Hani Baragwanath Academic Hospital
Rachel Moore
Maria Fourtounas
Mary Augusta Adam
Renessa Arumugan
Gabriella Hyman
Jaclyn Jonosky
Manigingingi Makondo
Heveshan Moodley
Phillip Munda
Mzwandile Nyalungu
Victor Olusola
Sohan Zane Pinto
Tristan Pillay
Lucinda Singh
Paul Mwindekuma Wondoh
John Devar

Dr George Mukhari Academic Hospital
Boitumelo Baloyi-Mnisi*
Zach Koto*
Tebogo Mabotja
Matlou Ernest Mabitsela
Sibongile Ruth Ndlovu
Branny Mthelebofu

Cecilia Maxiwane Hospital
Colin Beck*
Matthew Dold
Alice Fan
Shannon MacQueen
Thembani Matabata
Catherine Mpehle
Charné Kulenkampf

Pieterburg / Polokwane Hospital
Tsakani McCreath Mhlari*
Simangele Cecilia Nyoka- Mokgalong
Felix Thumba Masinge
Randhir Ramnath Gunpath

Maropeng Petrus Pat Mothwa
Jo-Anne Asenath Mothwa

Baberton District Hospital
Danai Mhlanga*
Jamie-Lyn Colly

Klerksdorp Hospital
Aunel Mallier Peter*
Khalid Ben Hameda

Job Shimankane Tabane Provincial Hospital
Pulane Mokae
Stella Josephine Moumakoe
Kelechi Ekeh

Kuruman Hospital
Nezingu Lengo*

Kimberley Hospital Complex
Marnus Booyens*
Inge Louise Seale
Pieter Daniel Theron
Nicolaas Abraham Schuman
Amber Carlyn Sonn
Jacobus Lukas Stander
Nadia Cloete
Marius Cloete
Catherine Ann Makepeace
Ronel van der Westhuizen
Leanne Robyn Messiahs
Amy Ruth Visagie
Fatima Vawda
Frans Christiaan Voster
Deepika Dhilraj

Charlotte Maxeke Johannesburg Academic Hospital
Oliver Smith*

Kalafong Academic Hospital
Daniel Montwedi*
Motsilisi Mbeki

Steve Biko Academic Hospital
Jayde Wyngaard*
Mthunzi Ngcelwane

Rahima Moosa Mother & Child Hospital
Thomas Kleyenstuber*
Phyllis Phukubye
Liesel Schärf

Tambo Memorial Hospital
Grace Laker*

Helen Joseph Hospital
Elizabeth Semenya*
Reinhard Dembskey
Thomas Tarlton
Tapiwa Jiri

Frontier Hospital
Ngoie Hubert Mushid*

Thelle-Mogoerane Hospital
Nhlanhla Samuel Ngwenya*
Hazel Morongoa Mogodi
Carmen Sinevici

Mthatha Regional Hospital

Anthony Osarogie Usenbo*
Naledi Fodo
Anesu Chimini
Ntetelelo Sikobi
Sinovuyo Nokwange
Mluleki Noqhamza
Qumba Thembisa

Frere Hospital
Kajake Anantha Padmanabha Bhat*
Robin Mathew
Katrin Middleton

Sebokeng Hospital
Abdus-sami Adewunmi*
Craig Dickson
Humairah Bulbulia

Potchefstroom Hospital
Bianka Bester
Michelle de Klerk

Rob Ferreira Hospital
Christia Benade
Francois Viljoen
Caroline Robertson*
Monique Fischer

Sudan. National Leader: Tarig Fadalla
Al Akadimiy Charity Hospital
Khalid Alfaki*

Saad Abul-Ella Teaching Hospital
Mohammed Osman*
Mazin Mohamed
Mohamed Elhasan
Abrar Widatalla

Ribat Teaching Hospital
Tarig Fadalla
Mazin Suliman*
Hanaa Mohammed
Lina Aljeally
Mohammed Dirar

Wad Medani Teaching Hospital
Abubakr Abubakr*
Eman Mohamed
Alshareef Nour

Port Sudan Maternity Hospital
Asia Elgailany*

El Fashir Teaching Hospital
Abdalmalik Awad*
Abdelsalam Algray
Mohammed Elsiddig
Suha Mohamed
Salih Mahmoud
Muhammed Osman

eSwatini. National Leader: Cynthia Lule
Hlathikhulu Government Hospital
Ntonto Doris Gama*

Mbabane Government Hospital
Dolorosa Khetsiwe Shabangu*
Cynthia Iradikunda

Releigh Fitkin Memorial Hospital
Samuel Mkoko*

Tanzania. National Leader: Bernard Mbwele, Mpoki Ulisubisya, Lazaro Mboma
Sumbawanga Regional Referral Hospital
Elias Chrisant*
Gilbert Msoma
Titus Kihwili
Gadiel Temu

Bukoba Regional Referral Hospital
Museleta Nyakiroto*
Emmanuel Jitambi
Venant Geofrey
Johaphes Josiah

Uwata Hospital-Mbeya
Edwin Edward*
Paul Kilamile*
Stephen Mwakyolile

Mazwi Health Centre
Jenitha Cheru*
Margaret Henjewele
Greyson Kilimanjaro
Sarah Sikimata

Bugando Medical Centre
Abel Makubi*
Vihar Kotecha*
Felician Kachinde

Sengerema Council Designated Hospital
Frederika Jager*
Charles Majani*

Rabininsia Memorial Hospital
Elijah Ussiri*
Gloria Kinasa
Patrick Adel
Eric Malaba
Vensesla Sakwari
Sadot Lugereka
Mohamed Mungia
George Mocha
Herman Wella
Cecilia Protas
Patrick Karua
Ahmada Kashagama
Faraja Mwasambugu
Suzana Kajeri
Jacquiline Mchilla

Muhimbili National Hospital - Mloganzila
Julieth Magandi*
Deogratius Manyama
Redempta Matindi
Adam Moshi
Daudi Kitwana
Merida Makia
Philip Muhochi
Miriam Herman
Clauda Miombo

Mbeya Zonal Referral Hospital
Bernard Mbwele
Mpoki Ulisubisya
Lazaro Mboma*

Deocles Donatus
Hazina Maduhu
Tumaini Mariro
Given Massasi
Moshi Moshi Shabani
Braison Cholela
Marco Mgeleka
Yohatinus Mbilinyi

Dodoma Regional Referral Hospital
Ernest Ibenzi*
Peter Mbelle*
Kato Peleus
Enid Chiwanga
Nillah Richard
Shoo Leonard
Paulo Sanka
Subira Mushi

Nyakahanga Hospital
Furaha Kahindo*
Langtone Kishebuka

Muhimbili National Hospital
Faraja Chiwanga*
Bilton Exavery
Caspar Haule
Samson Ndile
Sirili Harya

Shree Hindu Mandal Hospital
Ludovick Rukeha*
Tareeq Mohamed
Nabila Fuad
Winifrida Halinga

Bukumbi Mission Hospital
Phinius Makubi*
Frank Manumbu*
Suzan Mlingwa

Selian Lutheran Hospital
Elibariki Lucumay*
Robert Maise
Amon Marti
Beatrice Mahundi

Arusha Lutheran Medical Centre
Paul Kisanga*
Emmanuel Lema*
Benson Lyimo

Temeke Regional Referral Hospital
Hussein Msuma*
Eva Shang'a
Stella Ibrahim

Jakaya Kikwete Cardiac Institute
Bashir Nyangasa*
Mohamed Janabi
Naizihijwa Majani
Pedro Palangyo
Evarist Nyawawa
William Ramadhan
Faraj Lydenge

Benjamin Mkapa Hospital
Mohamed Binde*
Alphonce Chandika*
Salim Salim

Sylvia Jumbe

Tanga Regional Hospital / Bombo Regional Referral Hospital
Naima Yusuf*
Rashid Saleh
Rashid Inoja

Kilimanjaro Christian Medical Centre
Ansbert Ndebea*
Gileard Gabriel Masenga
Sakina Rashid
Mubashir Jusabani

Togo. National Leader: Hamza D Sama
CHU Kara
Talkana Adja*
Edem Gueouguede

Centre Hospitalier Universitaire Sylvanus Olympio de Lomé
Hafoudhoi Oussene Seddoh
Saliou Adam
Pilakimwe Egbohou
Mawunyo Ahomagnon
Olivier Kadjossou
Abdul-Bassiti Boukari

Uganda. National Leader: Adam Hewitt-Smith, Janat Tumukunde
International Hospital Kampala
Peter Kaahwa Agaba*
Daphne Kabatoro
Joseph Kayongo
Margaret Naggujja
Nabasiige Rehema

Uganda Heart Institute
Rita Nkwine*

Mengo Hospital
Gorret Nampiina*

Jinja Regional Referral Hospital
Daniel Kavuma
Aggrey Lubikire
Phiona Nansubuga*
Hope Bisilikirwa
Godfrey Ssebaggala

Masaka Regional Referral Hospital
Emmanuel Muwema*
Humble Joan Agaba
John Kiconco
Nicholas Wataaka
Bonet Chan
Mary Juliet Nampawu

St. Mary's Hospital Lacor
Peter Kayima*
Jacob Eyul
Erick Odwar

Mbale Regional Referral Hospital
Fred Bulamba*
Emmanuel Bua
Christine Mugala
Caroline Nyakato
John Paul Ochieng
Linda Kyomuhendo Jovia

Uganda Martyrs' Hospital Lubaga
Elizabeth Nabakka
Christine Namata*
Denis Kakaire

Mbarara Regional Referral Hospital
Rachel Alum
George Kateregga*
Lazia Najjuma

Nakasero Hospital Limited
Andrew Kintu*
Joshua Sempiira
Luzige Simon

China-Uganda Friendship Hospital
Ruth Muhindo
Mary T Nabukenya*
Peter Waswa

Zimbabwe. National Leader: Pisirai Ndarukwa
Gwanda Provincial Hospital
Elton Ndhlovu*
Zanele Mangwangwa
Nombulelo Dube

Parirenyatwa Hospital
Dennis Mazingi*
Chenesa Mbanje
Busisiwe Mlambo

Mpilo Central Hospital
Crispin Ntoto*
Derek Matsika

Marondera Provincial Hospital
Celestino Dhege*
Juliet Hungwa
Hemish Jasi

Mutare Provincial Hospital
Brightson Mutseyekwa*
Joseph Zimbovoora
Beaulah Gudyanga

Gweru Provincial Hospital
Velda Mushangwe-Mtisi*
Erisha Munhamo

Chitugwiza Central Hospital
Pisirai Ndarukwa*
Michael Chiwanga

Harare Central Hospital
Harunavamwe N Chifamba*

PSMI Westend Hospital
Sarudzai Zhou*
Esta Hove*
Shamiso Dende
Beauty Manjengwa
Penias Kapisa

The Avenues Clinic
Caritas Chiura
Locadia Katsukunya*
Godfrey Muguti
Doreen Mashava

Supplementary Material S1. ASOS-3 Protocol amendments

Protocol v2

Protocol v2 was the first approved ASOS-2 protocol by the UCT Human Research Ethics Committee (HREC)

v2 to v3 amendment

1. Page 2. Steering Committee: This has been changed to include essential local and international collaborators who have helped further refine the sample size and statistical analysis, and the trial processes.
2. Page 2. Funders of ASOS-2 have now been added to the protocol.
3. Page 6, 10, 14. Sample size amendment. Based on the coefficient of covariance of ASOS-1 we have increased the sample size to a more conservative number. This has required an increase in the recruitment period of potentially up to four weeks (in low recruiting sites). We have increased the recruitment period to four weeks at low surgical volume sites, as this decreases the coefficient of variance, which limits the sample size.
4. Page 18. Monitoring and auditing. Based on the increase in the sample size, and the potential increase in the duration of recruitment for some sites, we have added an independent international advisor (a world leading perioperative clinical trialist) who will advise on whether; i) hospital recruitment can continue after the planned recruitment window, and ii) whether an interim analysis would be required prior to continuing recruitment, if recruitment is slower than expected.
5. Page 11. Intervention arm. Based on the findings of the ASOS-2 Pilot Trial, we have made the following amendments; i) changed the definition of proximity to the nurses station, as a bed assigned to within view of the nurses' station, and ii) have added the 'Postoperative surveillance bedside guide' for all high-risk patients.
5. Page 18. Training of investigators. We have added 'remote electronic site initiation', as we believe it will be impossible for the national leaders to visit every site prior to the trial.
6. Page 6 and 20. We have increased the potential recruitment window to 4 months, as we believe that the sites will not all be ready to recruit within the same starting month.
7. The amended CRF's are attached. They also include capturing 'source data' of in-hospital mortality, which is the primary outcome.
8. There are minor grammatical changes in the protocol, to clarify (or correct the text) where questions have arisen from National Leaders, and other ASOS-2 investigators.

v3 to v4 amendment

Revision 1. Page 11. Section 6.3

i. Old wording:

Participating sites will be randomised to normal postoperative care or increased postoperative surveillance. Randomisation will be stratified according to the level of the surgical facility and the expected weekly surgical case-load.

ii. New wording:

Participating sites will be randomised to normal postoperative care ("SOC"), or increased postoperative surveillance ("Intervention") in four recruitment blocks (calendar time blocks). Randomisation in each block will be stratified by country and by level of the surgical facility with a fixed block size of 2. The randomisation algorithm will seek to balance randomisation arms by simulating randomisations with an automated filter to screen out poorly balanced randomisations. In the second and subsequent recruitment blocks, the randomisation algorithm will use knowledge of the previous randomisations and a similar simulation and filtering approach to ensure reasonable balance across arms and across level of surgical facility. This approach was developed on the basis of a simulation model for the randomisation that was used to evaluate the probability of overall balance of randomisation arms and balance within level of care strata if randomising recruitment phases in waves. Results of 10,000 simulation runs indicated that a balanced simulation within the bounds described below would be obtained >50% of the time without the filtering step.

The algorithm is described briefly here:
For recruitment phase 1:
Simulate stratified block randomisation within country and level of facility
Check that simulated randomisation has percent SOC between [48% - 52%]
If NO, then repeat steps 1 and 2. If YES then use this randomisation.
For all other recruitment phases:
Simulate stratified block randomisation for this phase within country and level of facility
Combine this simulation with prior actual randomisations
Check that combined randomisation has percent SOC between [48% - 52%]

If NO, then repeat steps 1-3. If YES proceed to next check.
Check that combined randomisation has balance within level of care between [45% - 55%]
If NO, then repeat steps 1-5. If YES then use this randomisation.

iii. Detailed rationale/ justification/ explanation:
The randomisation procedure was incompletely described in the previous protocol. The updated description of the randomisation procedure was tested through simulation and describes the procedure more comprehensively.

Revision 2. Pages 15-17. Section 7.5
i. Old wording:
Not applicable

ii. New wording:
7.5. Process evaluation

During the conduct of the ASOS-2 Trial we will perform a concurrent 'process evaluation' to gain insight into the implementation process. The process evaluation aims to:
1. Measure protocol compliance (fidelity of implementation),
2. Identify factors that influence success of implementation,
3. Verify specified process steps that form part of the implementation of the protocol and logical framework of the trial, and
4. Generate contextual information about the trial setting.

The questions asked by the process evaluation are:
1. Which factors determined fidelity of implementation at the local hospital level?
2. How did implementation of the intervention affect patient care?
3. Why does the intervention produce (or fail to produce) a change in patient morbidity and mortality?

The measurement tools used for the process evaluation are:
1. The post-education, REDCap online test,
2. The individual participant case record form (CRF),
3. An online database, called the screening log, which is a REDCap application for participating hospitals to record the number of eligible patients daily,
4. Research fellows will perform selected site visits in order to verify specified process steps and perform structured interviews with a sample of stakeholders,
5. Telephonic structured interviews with a sample of stakeholders that cannot be reached by the two research fellows, and
6. A post-trial, semi-quantitative, REDCap based, online questionnaire built around the key components identified in the structured interviews and the pilot trial.

Each member of the local hospital investigator team will be asked to complete the post-education online test prior to patient recruitment. The online test comprises eight short multiple-choice questions which test the comprehension of the key points from the education material.
The individual participant CRF (Appendix 3) captures information that allows checking accuracy of risk stratification, fidelity of implementation, and differences in patient experience between non-high risk patients in the two arms.
The research fellows will visit specific sites during the trial on appointed days agreed upon by the local hospital investigators. They will visit sites during the initiation phase, the recruitment phase, and the follow-up phase of the trial. They will use standard site visit checklists to identify non-compliance, barriers and facilitators of implementation. During the follow-up phase they will interview a sample of stakeholders who indicate willingness to participate in a qualitative interview about their personal experience of the trial. Verbal consent will be obtained and recorded for each interviewee who agrees to participate. No personal identifying information will be recorded for interviewees. Interviews will be recorded. A semi-structured script will be used for the interviews. Recordings will be stored on a password protected cloud based drive. Interviews will be analysed deductively by independent investigators using the Consolidated Framework for Implementation Research (CFIR) as a guide. The recordings will be destroyed following analysis.

The post-trial questionnaire will be anonymous. At the start of the questionnaire respondents will be informed about its content and intent. Respondents will be given the opportunity to opt out, or to give consent prior to continuing to the questions. Questions will test the CFIR constructs that were highlighted in the structured stakeholder interviews.

ii. Detailed rationale/ justification/ explanation:

The previous protocol did not describe any process evaluation. Various experienced international trialists urged the steering committee to describe a formal process evaluation as part of the trial. The process evaluation is essential for translation of the findings of the trial to subsequent clinical practice. I will allow improved understanding of the trial context which influences implementation and effectiveness of the intervention.

Revision 3. Page 19. Section 10

i. Old wording:

Not applicable

ii. New wording:

He [referring to the independent international advisor] will also check the event rate in the control arm of the study when at least 80% of all recruited patients have been captured on REDCap. If the event rate of the control arm is lower than predicted, the independent international advisor will use the event rate in the control arm to decide whether recruitment of further hospitals is required beyond the original sample size of 664 hospitals, in order to maintain 80% power for the primary outcome. This specified interim analysis of data will not lead to adjustment of the prespecified alpha of 0.05.

iii. Detailed rationale/ justification/ explanation:

Event rate driven recruitment is an established method to ensure that a trial does not conclude with a sample that is underpowered for the outcome(s) of interest. Such an outcome would waste the considerable time, effort and resources that have been invested by thousands of researchers across Africa. Testing the event rate does not test any of the outcomes of the trial and does therefore not incur any penalty for repeated testing.

Revision 4. Pages 37-38. Appendix C (case record forms)

i. Old wording:

Status at hospital discharge or 30th postoperative in-hospital day:

☐ Alive & still in hospital ☐ Dead ☐ Alive & discharged

ii. New wording:

Status at hospital discharge or 30th postoperative in-hospital day: ☐ Alive & still in hospital ☐ Dead

☐ Alive & discharged → if alive and discharged, was patient transferred to another facility for higher care? ☐ Yes ☐ No

iii. Detailed rationale/ justification/ explanation:

The same amendment is made to both the control and intervention case report forms. This amendment to the case report forms allows us to distinguish between patients who have been discharged home or to a convalescent facility (i.e. experienced a favourable outcome) compared to patients who were transferred to another hospital for higher level of care (i.e. lost to follow-up).

v4 to v5 amendment

Revision 1. Page 2.

iv. Old wording:

None

v. New wording:

6. Patrice Forget MD, PhD, Professor, Clinical Chair in Anaesthesia, Institute of Applied Health Sciences, School of Medicine, Medical Sciences and Nutrition, University of Aberdeen, United Kingdom. Honorary Consultant, NHS Grampian, Aberdeen, United Kingdom
7. Tim Stephens BA (Hons), MSc Global Health and Development, Critical Care and Peri-operative Medicine Research Group WHRI, Queen Mary University of London, E1 1BB, United Kingdom

vi. Detailed rationale/ justification/ explanation:

Two steering committee members added. Patrice Forget is a crucial member of the trial team and will be the only Francophone steering committee member. Tim Stephens is a crucial member of the process evaluation team bringing expertise in this area to the steering committee.

Revision 2. Pages 15. Section 7.1

iii. Old wording:

The inter-cluster correlation coefficient (ICC) in ASOS was 0.01.

iv. New wording:

The intra-cluster correlation coefficient (ICC) in ASOS was 0.01.

v. Detailed rationale/ justification/ explanation:

The correct term is intra-cluster correlation coefficient. This was a spelling mistake.

Revision 3. Pages 15. Section 7.1

i. Old wording:

Based on a relative risk reduction of 25% in the intervention arm, the power for the secondary outcome, based on the sample sizes for the primary outcome are shown in Table 1.

ii. New wording:

Based on a relative risk reduction of 25% in the intervention arm, the sample sizes for the primary outcome are shown in Table 1.

iii. Detailed rationale/ justification/ explanation:

The sample size refers to the primary outcome, not the secondary outcome.

v5 to v6 amendment

Revision 1. Page 7. Section 2.

vii. Old wording:

To determine whether increased postoperative surveillance reduces in-hospital mortality in high-risk adult surgical patients aged 18 years and over in Africa.

viii. New wording:

To determine whether increased postoperative surveillance in high-risk adult surgical patients reduces overall in-hospital mortality in surgical patients aged 18 years and over in Africa.

ix. Detailed rationale/ justification/ explanation:

The sentence structure of the outcome definition was corrected as it became apparent that the former version suggests we are only interested in the outcome of high-risk patients, whereas, we are principally interested in the outcome of all patients in the trial.

Revision 2. Page 7. Section 2.

vi. Old wording:

Recruitment end date August 2019

vii. New wording:

Recruitment end date March 2020

viii. Detailed rationale/ justification/ explanation:

At the end of August we had not reached our recruitment target. The progress of the trial was evaluated by our external auditor, Professor Paul Myles, Monash University, who advised that the trial continue. We plan to provide a next progress report to Professor Paul Myles during January 2020.

Revision 3. Page 9. Section 4.

iv. Old wording:

To determine whether increased postoperative surveillance reduces in-hospital mortality in high-risk adult surgical patients aged 18 years and over in Africa.

v. New wording:

To determine whether increased postoperative surveillance in high-risk adult surgical patients reduces overall in-hospital mortality in adult surgical patients aged 18 years and over in Africa.

vi. Detailed rationale/ justification/ explanation:

Duplication of revision 1.

Revision 4. Page 9. Section 4.

i. Old wording:

To determine whether increased postoperative surveillance reduces the incidence of the composite of severe in-hospital complications and mortality in high-risk adult surgical patients aged 18 years and over in Africa.

ii. New wording:

To determine whether increased postoperative surveillance in high-risk adult surgical patients reduces the overall incidence of the composite of severe in-hospital complications and mortality in adult surgical patients aged 18 years and over in Africa.

iii. Detailed rationale/ justification/ explanation:

Duplication of revision 1.

Revision 5. Pages 10. Section 5.

i. Old wording:

Recruitment will run in 2019.

ii. New wording:

Recruitment will run until March 2020.

iii. Detailed rationale/ justification/ explanation:

Duplication of revision 2.

Revision 6. Page 10. Section 5.

i. Old wording:

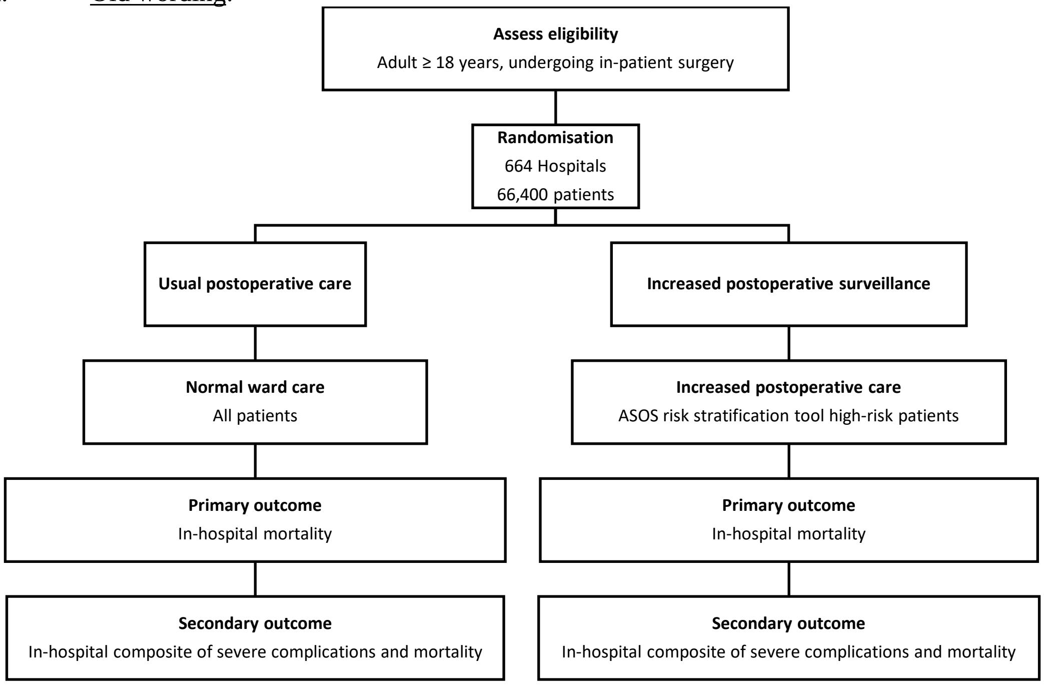

ii. New wording:

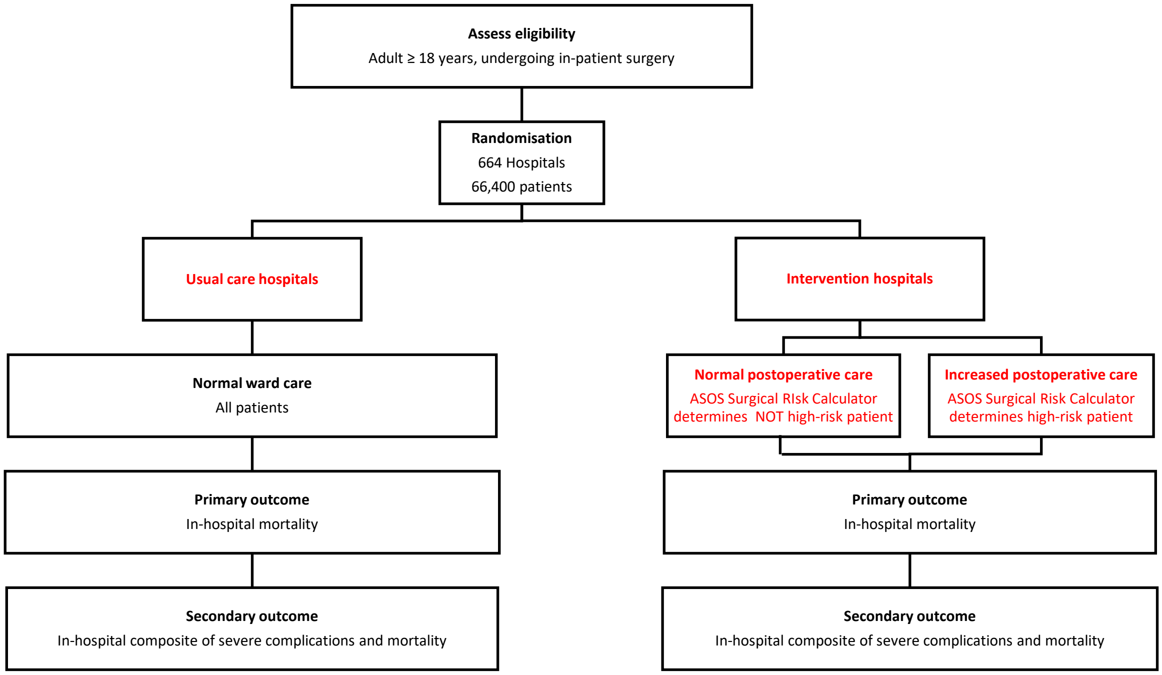

iii. Detailed rationale/ justification/ explanation:

The flow diagram was updated to make it clear that the hospital is the unit of randomisation in this trial and that within intervention hospitals it is only the subset of high-risk patients who should receive increased postoperative surveillance.

v6 to v7 amendment

The statistical analysis plan was added to version 6.

Supplementary Material S2. Broadcasting document

This hospital is participating in a surgical trial

This hospital is participating in a surgical trial, known as the African Surgical OutcomeS-2 (ASOS-2) Trial. The ASOS-2 Trial will determine whether increased postoperative surveillance reduces in-hospital death in high-risk adult surgical patients across Africa. Hospitals are randomised to provide usual care, or increased surveillance of high-risk patients. All adult patients aged 18 years and over who have surgery in this hospital are included in this trial. All patients in this hospital will receive similar postoperative surgical management, based on which arm of the trial that this hospital has been randomised to.
Potential harm to participants: We do not believe that this trial provides any potential harms to participants. You may not benefit directly from participation in this trial, however the results of this trial may help us to provide better care to future surgical patients in Africa.
Confidentiality: The study doctor and his/her study team will look at your personal health information and collect only the information they need for the study. This information includes:

- Your name, gender and date of birth
- Information about your surgery, and test results before your surgery and while you are in hospital after the surgery
- Information about your health status and health outcomes

The information that is collected for the study will be kept in a locked and secure area by the study doctor. Only the study team or the people or groups listed below will be allowed to look at your records. Your participation in this study also may be recorded in your medical record at this hospital. The following people may come to the hospital to look at the study records and at your personal health information to check that the information collected for the study is correct and to make sure the study followed proper laws and guidelines:

- The study lead investigator(s) or his/her representatives
- Representatives of the University of Cape Town Research Ethics Board

Some of your health information will be sent outside of the hospital to the trial lead investigators and stored at a secure research database hosted by Safe Surgery South Africa, of the South African Society of Anaesthesiologists. Any information about you that is sent out of the hospital will have a code and will not show your name or address, or any information that directly identifies you. All information collected during this study, including your personal health information, will be kept confidential and will not be shared with anyone outside the study unless required by law. You will not be named in any reports, publications, or presentations that may come from this study.
Treatment Options: If you choose not to be in this study you will still get the best available treatment and be managed according to standard international guidelines. If you decide to leave the study, the information about you that was collected before you left the study will still be used. No new information will be collected without your permission.
Publication of Results: The results of this study may be presented at conferences or seminars and may be published in a medical journal. All information during presentations will be anonymous. Study results will be available from the study investigators once the study is complete and the data is analysed.
Potential Costs of Participation and Reimbursement to the Participant: There is no cost to participate in this study. You will not be reimbursed for your participation.
Compensation for Injury: If you suffer a physical injury from participation in this study, medical care will be provided to you in the same manner as you would ordinarily obtain any other medical treatment. Participation in this trial does not waive your legal rights nor release the study doctors, the hospital from their legal and professional responsibilities. Reasonable medical expenses incurred through research-related injuries will be covered by the University of Cape Town's no-fault insurance cover.
Participation and Withdrawal: Participation in any research study is voluntary. If you choose not to participate, you and your family will continue to have access to surgical care at this hospital. If you decide to participate in this study, you can change your mind without giving a reason, and you may withdraw from the study at any time without any effect on the care you and your family will receive any Hospital.
New Information: If the trial investigators learn new information that affects your participation, you will be informed in a timely manner.
Research Ethics Board Contact: If you have any questions regarding your rights as a research participant, you may contact Administrator, Human Research Ethics Committee during business hours.
Contact: E 53 Room 46, Old Main Building, Groote Schuur Hospital, Observatory, Cape Town
Telephone: 27 21 406 6492
Email: nosi.tsama@uct.ac.za or shuretta.thomas@uct.ac.za
The study protocol and consent form have been reviewed by a committee called the Human Research Ethics Committee at University of Cape Town. The Human Research Ethics Committee is a group of scientists, medical staff, and individuals from other backgrounds (including law and ethics) as well as members from the community. The committee is established by the hospital to review studies for their scientific and ethical merit. The Board pays special attention to the potential harms and benefits involved in participation to the research participant, as well as the potential benefit to society.
This committee is also required to do periodic review of ongoing research studies. As part of this review, someone may contact you from the Human Research Ethics Committee to discuss your experience in the research study.

Study Contacts: If you require any further information or have any concerns, please feel free to contact the investigators or study coordinator: _______________________________

Supplementary Material S3. Randomisation algorithm

The randomisation algorithm is described briefly here:
For recruitment phase 1:

1. Simulate stratified block randomisation within country and level of facility.
2. Check that simulated randomisation has percent ‘standard of care’ between [48% - 52%].
3. If NO, then repeat steps 1 and 2. If YES then use this randomisation.

For all other recruitment phases:

1. Simulate stratified block randomisation for this phase within country and level of facility.
2. Combine this simulation with prior actual randomisations.
3. Check that combined randomisation has percent ‘standard of care’ between [48% - 52%].
4. If NO, then repeat steps 1-3. If YES proceed to next check.
5. Check that combined randomisation has balance within level of care between [45% - 55%].
6. If NO, then repeat steps 1-5. If YES then use this randomisation.

Supplementary Material S4. Postoperative surveillance bedside guide

ASOS-2
African Surgical OutcomeS-2 Trial

‘Increased postoperative surveillance’ bedside guide

This is a ‘high-risk’ surgical patient

This patient requires ‘increased postoperative surveillance’ and is most likely to experience one of these complications postoperatively

‘High-risk’ patients most commonly experience the following complications.

Most common complications	Consider the following management
Cardiac arrest	**Ensure staff know where the emergency drugs and defibrillator are stored**
Surgical site infections	**Swab wound for microbiology, and consider antibiotics**
Bloodstream infections	**Consider early blood cultures, and consider antibiotics**
Pneumonia	**Physiotherapy, mobilise early, sputum & blood cultures, & consider antibiotics**
Acute kidney injury	**Ensure adequate hydration, monitor the serum creatinine**
Postoperative bleed	**Look for signs of hypotension, and decreasing haemoglobin**

If for any reason you are concerned about the patient’s condition postoperatively,

then please look for these complications

Supplementary Material S5. Case record form: Control arm

ASOS-2
African Surgical OutcomeS-2 Trial

Age ☐☐☐ years Sex ☐ M ☐ F

ASA ☐ I ☐ II ☐ III ☐ IV ☐ V

Chronic co-morbid disease (tick all that apply): ☐ Hypertension ☐ HIV / AIDS ☐ Diabetes mellitus ☐ COPD / Asthma

Surgical procedure category (select *single* most appropriate):

☐ Gynaecology ☐ Obstetrics ☐ Orthopaedic ☐ Ear, nose and throat ☐ Plastics or breast

☐ Urology ☐ Neurosurgery ☐ Gastro-intestinal or Hepato-biliary ☐ Cardiothoracic/ vascular ☐ Other

Indication for surgery:

☐ Non-communicable disease ☐ Caesarean section ☐ Trauma ☐ Infection

Urgency of surgery: ☐ Elective ☐ Urgent ☐ Emergency

Severity of surgery: ☐ Minor ☐ Intermediate ☐ Major

Start of surgery time (24h) & date: h h : m m d d m m 2 0 1 9

Postoperative Follow Up

Indicate postoperative care given:

Higher care ward ☐ No ☐ Yes

Increased nursing observations ☐ No ☐ Yes

Assigned a bed in view of nurses' station ☐ No ☐ Yes

Family with patient in ward ☐ No ☐ Yes

Severe complications (tick *all* that apply): Superficial or deep surgical site, or body cavity infection ☐ N ☐ Y Postop day ☐☐

Bloodstream infection or ARDS ☐ N ☐ Y Postop day ☐☐ Pneumonia ☐ N ☐ Y Postop day ☐☐

Urinary tract or AKI ☐ N ☐ Y Postop day ☐☐ Postoperative bleed ☐ N ☐ Y Postop day ☐☐

Cardiac arrest ☐ N ☐ Y Postop day ☐☐ Other severe complication ☐ N ☐ Y Postop day ☐☐

Days in hospital after surgery ☐☐

Status at hospital discharge or 30th postoperative in-hospital day: ☐ Alive & still in hospital ☐ Dead

☐ Alive & discharged → if alive and discharged, was patient transferred to another facility for higher care? ☐ Yes ☐ No

If deceased, photo of clinical note of death uploaded: ☐ Yes CRF completed and verified by……………. on dd/mm/2019

ASOS-2 unique patient ID ☐☐☐☐☐☐☐☐☐☐ ASOS-2 Trial CRF v4 (Control arm)

✂ -

Patient name: ___________________________________ DOB d d m m y y y y

Patient hospital number : _________________ ASOS-2 unique patient ID ☐☐☐☐☐☐☐☐☐☐

Supplementary Material S6. Case record form: Intervention arm

ASOS-2
African Surgical OutcomeS-2 Trial

Age ☐☐☐ years (<30 points; 0 points/ 30-69 years; 1 point/ ≥70 years; 3 points)   Sex ☐ M ☐ F

ASA ☐ I (0 points) ☐ II (2 points) ☐ III (5 points) ☐ IV (8 points) ☐ V (8 points)

Chronic co-morbid disease (tick all that apply): ☐ Hypertension ☐ HIV / AIDS ☐ Diabetes mellitus ☐ COPD / Asthma

Surgical procedure category (select *single* most appropriate): ☐ Gynaecology (minus 1 point) ☐ Obstetrics (minus 1 point)

☐ Orthopaedic (0 points) ☐ Ear, nose and throat (3 points) ☐ Plastics or breast (1 point) ☐ Urology (2 points)

☐Neurosurgery(4 points) ☐Gastro-intestinal or Hepato-biliary(3 points) ☐Cardiothoracic/ vascular(3 points) ☐Other(0 points)

Indication for surgery:

☐ Non-communicable disease (0 points) ☐ Caesarean section (minus 2 points) ☐ Trauma (1 point) ☐ Infection (2 points)

Urgency of surgery: ☐ Elective (0 points) ☐ Urgent (3 points) ☐ Emergency (4 points)

Severity of surgery: ☐ Minor (0 points) ☐ Intermediate (2 points) ☐ Major (4 points)

Start of surgery time (24h) & date: h h : m m   d d m m 2 0 1 9

ASOS Surgical Risk Score points per risk factor:

☐Age + ☐ASA + ☐Surgical procedure category + ☐Indication for surgery + ☐Urgency surgery + ☐Severity surgery = ☐☐ points

Time that the ASOS Surgical Risk Score was calculated: ☐ Pre-op ☐ Intra-op ☐ Immediately post-op

Predicted ASOS Risk Score: ☐ Not high-risk patient (<10 points) ☐ High-risk patient (≥10 points)

Postoperative Follow Up

☐ Not high-risk patient: (*Indicate postoperative care given*): Higher care ward ☐No ☐Yes Increased nursing observations ☐No ☐Yes

Assigned a bed in view of nurses' station ☐ No ☐ Yes   Family with patient in ward ☐ No ☐ Yes

☐ High-risk patient: (*Indicate all postop surveillance*)	Day 0	Day 1	Day 2	Day 3	Day 4+
Higher care ward	☐No ☐Yes	☐No ☐Yes	☐No ☐Yes	☐No ☐Yes	☐No ☐Yes
Increased nursing observations	☐No ☐Yes	☐No ☐Yes	☐No ☐Yes	☐No ☐Yes	☐No ☐Yes
Assigned a bed in view of nurses' station	☐No ☐Yes	☐No ☐Yes	☐No ☐Yes	☐No ☐Yes	☐No ☐Yes
Family with patient in ward	☐No ☐Yes	☐No ☐Yes	☐No ☐Yes	☐No ☐Yes	☐No ☐Yes
'Postoperative surveillance bedside guide' at the patient's bedside?	☐No ☐Yes	☐No ☐Yes	☐No ☐Yes	☐No ☐Yes	☐No ☐Yes

Severe complications (tick *all* that apply): Superficial or deep surgical site, or body cavity infection ☐ N ☐ Y Postop day ☐☐

Bloodstream infection or ARDS ☐ N ☐ Y Postop day ☐☐   Pneumonia ☐ N ☐ Y Postop day ☐☐

Urinary tract or AKI ☐ N ☐ Y Postop day ☐☐   Postoperative bleed ☐ N ☐ Y Postop day ☐☐

Cardiac arrest ☐ N ☐ Y Postop day ☐☐   Other severe complication ☐ N ☐ Y Postop day ☐☐

Days in hospital after surgery ☐☐

Status at hospital discharge or 30th postoperative in-hospital day: ☐ Alive & still in hospital ☐ Dead

☐ Alive & discharged → if alive and discharged, was patient transferred to another facility for higher care? ☐ Yes ☐ No

If deceased, photo of clinical note of death uploaded ☐ Yes   CRF completed and verified by................ on dd/mm/2019

ASOS-2 unique patient ID ☐☐☐☐☐☐☐☐☐☐   **ASOS-2 Trial CRF v4 (Intervention arm)**

✂ -

Patient name: ______________________________   **DOB** d d m m y y y y

Patient hospital number : _______________   **ASOS-2 unique patient ID** ☐☐☐☐☐☐☐☐☐☐

Supplementary Material S7. Site initiation checklists

**ASOS-2 HOSPITAL LEADER CHECKLIST (CONTROL ARM)**	☑
Site initiation educational material attached	☑
Upload ethics approval	☐
Upload Hospital Leader GCP certificate	☐
**Weeks three and two before recruitment begins**<br>Identify local ASOS-2 team of at least two people, ideally three or more	☐
Hospital Leader to educate Local ASOS-2 team with site initiation educational material	☐
Local ASOS-2 team to complete site initiation online questionnaire after completing educational material	☐
Local ASOS-2 team decides who will perform each task (data collection, follow up)	☐
Local ASOS-2 team members to educate all involved departments e.g. surgery department etc., about the trial	☐
**One week before recruitment begins**<br>Placement of trial advisory ('broadcasting') posters in patient care areas	☐
**Monday of start of recruitment**<br>Trial begins	☐
Trial recruitment ends at i) the end of the week when the total recruited patients exceeds 100 patients, or ii) after 4 weeks recruitment	☐
Hospital Leader or designee uploads physical CRFs into electronic database	☐

**ASOS-2 HOSPITAL LEADER CHECKLIST (INTERVENTION ARM)**	☑
Site initiation educational material attached	☑
Upload ethics approval	☐
Upload Hospital Leader GCP certificate	☐
**Weeks three and two before recruitment begins**Identify local ASOS-2 team of at least two people, ideally three or more	☐
Hospital Leader to educate Local ASOS-2 team with site initiation educational material	☐
Local ASOS-2 team to complete site initiation online questionnaire after completing educational material	☐
Local ASOS-2 team decides who will perform each task (risk calculation, interventions, follow up)	☐
Local team decides how interventions will be instituted in the hospital	☐
Local ASOS-2 team members to educate all involved departments e.g. surgery department etc., about the trial and the intervention procedures	☐
Local ASOS-2 team members to educate anaesthetists or designees about how to use the ASOS Surgical Risk Calculator and identify high-risk patients	☐
**One week before recruitment begins**Placement of trial advisory ('broadcasting') posters in patient care areas	☐
Two-day ASOS-2 Trial test run to identify barriers to providing 'increased postoperative surveillance'	☐
Determine solutions to barriers identified in the test run	☐
**Monday of start of recruitment**<br>Trial begins	☐
Trial recruitment ends at i) the end of the week when the total recruited patients exceeds 100 patients, or ii) after 4 weeks recruitment	☐
Hospital Leader or designee uploads physical CRFs into electronic database	☐

Supplementary Material S8. Definitions of the components of the secondary outcome ‘severe complications’

These definitions are based on the ‘Standards for definitions and use of outcome measures for clinical effectiveness research in perioperative medicine: European Perioperative Clinical Outcome (EPCO) definitions: a statement from the ESA-ESICM joint taskforce on perioperative outcome measures’.[1]
A severe complication is one that results in significant prolongation of hospital stay and/or permanent functional limitation or death. Almost always requires clinical treatment. The following are considered severe complications in the ASOS-2 trial:

I. Surgical site infection (superficial)

Infection involving only superficial surgical incision which meets the following criteria:

a. Infection occurs within 30 days after surgery and
b. Involves only skin and subcutaneous tissues of the incision and
c. The patient has at least one of the following:
   i. purulent drainage from the superficial incision
   ii. organisms isolated from an aseptically obtained culture of fluid or tissue from the superficial incision and at least one of the following signs or symptoms of infection: pain or tenderness, localized swelling, redness, or heat, or superficial incision is deliberately opened by surgeon and is culture positive or not cultured. A culture-negative finding does not meet this criterion.
   iii. diagnosis of an incisional surgical site infection by a surgeon or attending physician

II. Surgical site infection (deep)

An infection which involves both superficial and deep parts of surgical incision and meets the following criteria:

a. Infection occurs within 30 days after surgery if no surgical implant is left in place or one year if an implant is in place and
b. The infection appears to be related to the surgical procedure and involves deep soft tissues of the incision (e.g. fascial and muscle layers) and
c. The patient has at least one of the following:
   i. purulent drainage from the deep incision but not from the organ/space component of the surgical site
   ii. a deep incision spontaneously dehisces or is deliberately opened by a surgeon and is culture-positive or no cultures were taken whilst the patient has at least one of the following signs or symptoms of infection: fever (>38°C) or localized pain or tenderness. A culture-negative finding does not meet this criterion.
   iii. an abscess or other evidence of infection involving the deep incision is found on direct examination, during surgery, or by histopathologic or radiologic examination
   iv. diagnosis of a deep incisional surgical site infection by a surgeon or attending physician

III. Surgical site infection (organ/space)

An infection which involves any part of the body excluding the fascia or muscle layers and meets the following criteria:

a. Infection occurs within 30 days after surgery and
b. The infection appears to be related to the surgical procedure and involves any part of the body, excluding the skin incision, fascia, or muscle layers, that is opened or manipulated during the operative procedure and
c. The patient has at least one of the following:
   i. purulent drainage from a drain that is placed through a stab wound into the organ/space
   ii. organisms isolated from an aseptically obtained culture of fluid or tissue in the organ/ space
   iii. an abscess or other evidence of infection involving the organ/space that is found on direct examination, during reoperation, or by histopathologic or radiologic examination
   iv. diagnosis of an organ/space surgical site infection by a surgeon or attending physician

IV. Bloodstream infection

An infection which is not related to infection at another site and which meets at least one of the following criteria:

a. Patient has a recognised pathogen cultured from blood cultures which is not related to an infection at another site
b. Patient has at least one of the following signs or symptoms: fever (>38°C), chills, or hypotension and at least one of the following:
   i. common skin contaminant cultured from two or more blood cultures drawn on separate occasions
   ii. common skin contaminant cultured from at least one blood culture from a patient with an intravascular line, and a physician starts antimicrobial therapy
   iii. positive blood antigen test

V. Acute Respiratory Distress Syndrome (ARDS)

Respiratory failure, or new or worsening respiratory symptoms, commencing within one week of surgery; and a chest radiograph or computed tomography scan which demonstrates bilateral opacities not fully explained by effusions, lobar/lung collapse, or nodules; and respiratory failure not fully explained by cardiac failure or fluid overload. Need objective assessment (e.g. echocardiography) to exclude hydrostatic oedema if no risk factor is present.
Severity grading:
Severe: PaO_2:FiO_2 ≤100 mmHg with PEEP ≥5 cmH_2O
Guidance:
If altitude is higher than 1000 m, a correction factor should be calculated as follows: (PaO_2:FiO_2 x [barometric pressure/760 mmHg]). PEEP, positive end-expiratory pressure; CPAP, non-invasive continuous positive airways pressure

VI. Pneumonia

Chest radiographs with new or progressive and persistent infiltrates, or consolidation, or cavitation, and at least one of the following:

a. fever (>38°C) with no other recognized cause
b. leucopaenia (<4,000 white blood cells/mm3) or leucocytosis (>12,000 white blood cells/mm3)
c. for adults >70 years old, altered mental status with no other recognised cause;
d. and at least two of the following:
    i. new onset of purulent sputum or change in character of sputum, or increased respiratory secretions, or increased suctioning requirements
    ii. new onset or worsening cough, or dyspnoea, or tachypnoea
    iii. rales or bronchial breath sounds
    iv. worsening gas exchange (hypoxaemia, increased oxygen requirement or increased ventilator demand)

Guidance: Two radiographs are required for patients with underlying pulmonary or cardiac disease. The definition may be used to identify ventilator associated pneumonia.

VII. Urinary tract infection

An infection associated with at least one of the following signs or symptoms which should be identified within a 24 hour period; fever (>38 °C), urgency, frequency, dysuria, suprapubic tenderness, costovertebral angle pain or tenderness with no other recognised cause, and a positive urine culture of ≥105 colony forming units/mL with no more than two species of microorganisms.

VIII. Acute Kidney Injury (AKI)

Acute Kidney Injury (AKI) Stage	Serum creatinine	Urine output
Severe	Increase of 3.0 times baseline within 7 days or increase in serum creatinine to ≥4.0 mg/dL (≥354 μmol/L) with an acute rise of >0.5 mg/dL (>44 μmol/L) or initiation of renal replacement therapy	≤0.3 ml/kg/h for 24 hours<br><br>or<br><br>Anuria for 12 hours

Guidance: Baseline serum creatinine must be measured before surgery but an estimated value can be used if the patient does not have chronic kidney disease.

IX. Postoperative haemorrhage

Blood loss occurring within 72 hours after the end of surgery which would normally result in transfusion of blood.

X. Cardiac arrest

The cessation of cardiac mechanical activity, as confirmed by the absence of signs of circulation. ECG changes may corroborate the incidence of cardiac arrest.

XI. Other severe complications

If any of the following complications result in a significant prolongation of hospital stay and/or permanent functional limitation or death, then mark ‘Other severe complication’ as ‘Yes’. Note that they will almost always requires clinical treatment.

a. Anastomotic breakdown

Leak of luminal contents from a surgical connection between two hollow viscera. The luminal contents may emerge either through the wound or at the drain site, or they may collect near the anastomosis, causing fever, abscess, septicaemia, metabolic disturbance and/or multiple-organ failure. The escape of luminal contents from the site of the anastomosis into an adjacent localised area, detected by imaging, in the absence of clinical symptoms and signs should be recorded as a sub-clinical leak.

b. Arrhythmia

Electrocardiograph (ECG) evidence of cardiac rhythm disturbance.

c. (Cardiogenic) pulmonary oedema

Evidence of fluid accumulation in the alveoli due to poor cardiac function.

d. Gastro-intestinal bleed

Unambiguous clinical or endoscopic evidence of blood in the gastro-intestinal tract. Upper gastrointestinal bleeding is that originating from the oesophagus, stomach and duodenum. Lower gastro-intestinal bleeding originates from the small bowel and colon.

e. Myocardial infarction

Increase in serum cardiac biomarker values (preferably cardiac troponin) with at least one value above the 99th percentile upper reference limit and at least one of the following criteria:

    i. Symptoms of ischaemia
    ii. New or presumed new ST-segment or T-wave ECG changes or new left bundle branch block
    iii. Development of pathological Q-waves on ECG

iv. Radiological or echocardiographic evidence of new loss of viable myocardium or new regional wall motion abnormality
v. Identification of an intra-coronary thrombus at angiography or autopsy

f. Pulmonary embolism (PE)

A new blood clot or thrombus within the pulmonary arterial system.
Guidance: Appropriate diagnostic tests include scintigraphy and CT angiography. Plasma D-dimer measurement is not recommended as a diagnostic test in the first three weeks following surgery.

I. Stroke

Embolic, thrombotic, or haemorrhagic cerebral event with persistent residual motor, sensory, or cognitive dysfunction (e.g. hemiplegia, hemiparesis, aphasia, sensory deficit, impaired memory).

Reference

1. Jammer I, Wickboldt N, Sander M, et al. Standards for definitions and use of outcome measures for clinical effectiveness research in perioperative medicine: European Perioperative Clinical Outcome (EPCO) definitions: a statement from the ESA-ESICM joint taskforce on perioperative outcome measures. Eur J Anaesthesiol 2015;32(2):88-105. doi: 10.1097/EJA.0000000000000118

Supplementary Material S9. The ASOS-2 trial design

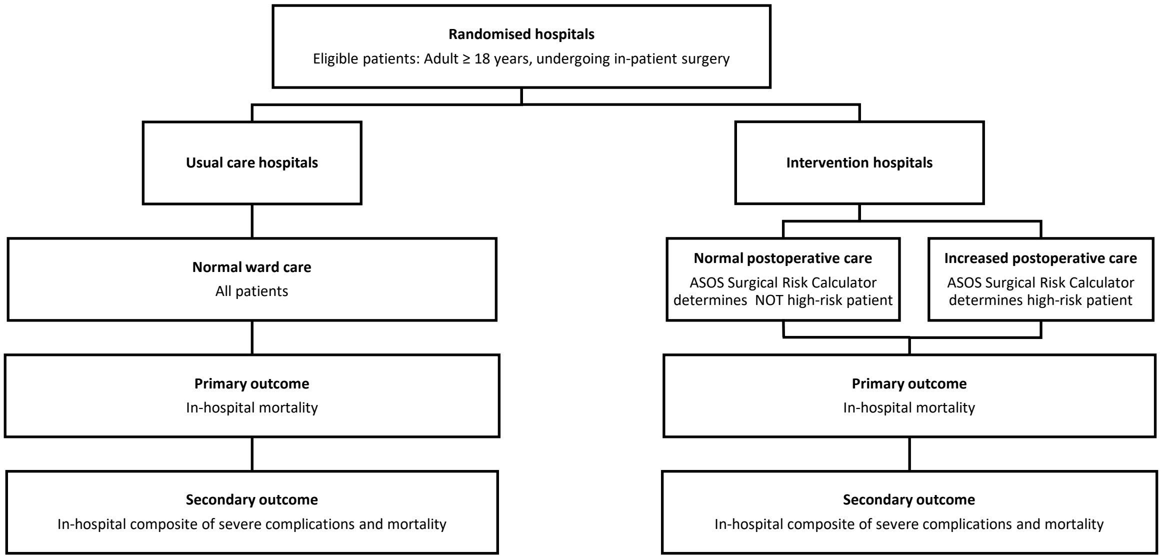

Supplementary Material S10: Sample size calculations for the ASOS-2 Trial based on a power of 80%, 2-sided α = 0.05 and a mean cluster size of 100 patients

**Primary outcome (in-hospital all-cause mortality)**					
**Control event rate**	**Intervention arm**	**Relative risk**	**Intra-cluster correlation coefficient (ICC)**	**Coefficient of variance (CV)**	**Total clusters**
2.0%	1.5%	0.75	0.015	0	536
2.0%	1.5%	0.75	0.015	0.63	664
2.0%	1.6%	0.80	0.015	0.63	1068

Supplementary Material S11. Prespecified sensitivity and subgroup analyses

Other prespecified sensitivity and subgroup analyses on the effectiveness outcomes were also conducted; i) the potential impact of unobserved outcomes on the primary and secondary effectiveness estimates, ii) subgroups treated as stratification variables; including hospital level, recruitment wave, and income category of country, and iii) analysis stratified by individual patient and surgical characteristics that form part of the ASOS Surgical Risk Calculator. The full prespecified additional secondary analyses are shown below.

The following prespecified sensitivity and subgroup analyses on the effectiveness outcomes were also conducted.
1. Sensitivity analyses investigated the potential impact of unobserved outcomes (transfer out or lost to follow up) on the primary and secondary effectiveness estimates, where individuals with unobserved outcomes were alternately assumed to be alive at discharge (censored at thirty days) or to have died in-hospital within 30 days.
2. Primary endpoints were also analysed under the same model as the primary analysis for the following subgroups treated as stratification variables: i) hospital level (primary, secondary, tertiary), iii) recruitment wave, and iii) income category of country (low or middle, according to the World Bank classification in 2020).
3. The relative risk of experiencing the primary and secondary outcome by exposure to the intervention (defined below) was estimated in stratified models for the following individual characteristics: i) age (years), ii) American Society of Anesthesiologists (ASA) status (1-5), iii) surgical procedure category (gynaecologic, obstetric, neurosurgery, ear nose and throat (ENT), orthopaedic, plastics and breast, urology, gastrointestinal and hepatobiliary, cardio-thoracics, and other), iv) indication for surgery (non-communicable disease, caesarean section, trauma, infection), v) urgency of surgery (elective, urgent, emergent), and vi) severity of surgery (minor, intermediate, major).
(Secondary analyses included two analytic approaches to two per protocol populations, based on implementation fidelity. In the first per protocol analysis, we compared all patients from hospitals with data in the standard of care arm to all patients from hospitals with data in the intervention arm where the intervention hospital provided the intervention with fidelity to at least 80% of high-risk patients. Patients from hospitals in the intervention arm where the hospital delivered the intervention with fidelity to fewer than 80% of patients will be excluded from this analysis. In the second per protocol analysis, we compared all patients from hospitals with data in the standard of care arm to all patients from hospitals in the top two tertiles of implementation fidelity. Patients from intervention hospitals in the bottom tertile of implementation fidelity were excluded from this analysis. At the hospital level we report implementation fidelity as the proportion of high-risk patients who received the intervention with fidelity. We used two definitions for implementation fidelity; i) provision of at least the high-risk bedside guide plus one additional component of the intervention on days 0 and 1 postoperatively and ii) provision of at least 2 components of the intervention on days 0 and days 1 postoperatively, regardless whether the high-risk beside guide is one of the components.)

Supplementary Table S1. Hospitals and participants recruited per randomisation wave in the trial

Randomisation wave	Hospitals (n=332)	Patients (n=28855)
Wave 1	63 (19·0%)	6479 (22·5%)
Wave 2	39 (11·7%)	2269 (7·9%)
Wave 3	35 (10·5%)	985 (3·4%)
Wave 4	55 (16·6%)	7025 (24·3%)
Wave 5	66 (19·9%)	5964 (20·7%)
Wave 6	13 (3·9%)	1047 (3·6%)
Wave 7	10 (3·0%)	984 (3·4%)
Wave 8	6 (1·8%)	597 (2·1%)
Wave 9	4 (1·2%)	320 (1·1%)
Wave 10	19 (5·7%)	1238 (4·3%)
Wave 11	22 (6·6%)	1947 (6·7%)

Supplementary Table S2. Hospitals recruited in the trial

	All patients (n=28892)	Increased postoperative surveillance group (n=13275)	Standard of care group (n=15617)
Primary	3846 (13·3%)	1977 (14·9%)	1869 (12·0%)
Secondary	10094 (35·0%)	4442 (33·5%)	5652 (36·3%)
Tertiary	14952 (51·8%)	6856 (51·6%)	8096 (51·8%)

Supplementary Table S3. Physician surgeons, anaesthesiologists and obstetricians, and the number of operating theatres in hospitals recruited in the trial

	All hospitals (n=169)	Increased postoperative surveillance group (n=76)	Standard of care group (n=93)	p-value
**Primary hospitals**	n=36	n=14	n=22	
Physician surgeon	1 (0-4)	2 (0-3)	1 (0-6)	0.74
Physician anaesthesiologist	2 (0-5)	3 (2-7)	1 (0-5)	0.10
Physician obstetrician	2 (0-4)	3 (0-4)	1 (0-5)	0.58
**Secondary hospitals**	n=60	n=27	n=33	
Physician surgeon	4 (1-9)	4 (1-7)	4 (0-10)	0.81
Physician anaesthesiologist	4 (0-8)	5 (0-9)	2 (0-9)	0.54
Physician obstetrician	4 (1-8)	4 (1-8)	3 (0-9)	0.71
**Tertiary hospitals**	n=73	n=35	n=38	
Physician surgeon	8 (2-25)	8 (3-25)	8 (1-24)	0.49
Physician anaesthesiologist	8 (2-16)	8 (2-18)	7 (1-15)	0.34
Physician obstetrician	9 (3-20)	8 (2-20)	10 (1-19)	0.94

Supplementary Table S4. ASOS Surgical Risk Calculator Scores in participating patients

	All patients (n=28892)	Enhanced postoperative surveillance group (n=13275)	Standard of care group (n=15617)
Missing	936	261	675
-3	141 (0·5%)	100 (0·8%)	41 (0·3%)
-2	58 (0·2%)	23 (0·2%)	35 (0·2%)
-1	373 (1·3%)	176 (1·4%)	197 (1·3%)
0	653 (2·3%)	275 (2·1%)	378 (2·5%)
1	931 (3·3%)	438 (3·4%)	493 (3·3%)
2	2302 (8·2%)	970 (7·5%)	1332 (8·9%)
3	2537 (9·1%)	1176 (9·0%)	1361 (9·1%)
4	2643 (9·5%)	1289 (9·9%)	1354 (9·1%)
5	3813(13·6%)	1737 (13·1%)	2076 (13·9%)
6	3093(11·1%)	1499 (11·5%)	1594 (10·2%)
7	2292 (8·2%)	1110 (8·5%)	1182 (7·9%)
8	2214 (7·9%)	1028 (7·9%)	1186 (7·9%)
9	1336 (4·8%)	648 (5·0%)	688 (4·6%)
10	1186 (4·2%)	527 (4·0%)	659 (4·4%)
11	1118 (4·0%)	513 (3·9%)	605 (4·0%)
12	752 (2·7%)	358 (2·8%)	394 (2·6%)
13	694 (2·5%)	322 (2·5%)	372 (2·5%)
14	489 (1·7%)	225 (1·7%)	264 (1·8%)
15	311 (1·1%)	149 (1·1%)	162 (1·1%)
16	302 (1·1%)	117 (0·9%)	185 (1·2%)
17	199 (0·7%)	104 (0·8%)	95 (0·6%)
18	178 (0·6%)	85 (0·7%)	93 (0·6%)
19	125 (0·4%)	65 (0·5%)	60 (0·4%)
20	87 (0·3%)	28 (0·2%)	59 (0·4%)
21	78 (0·3%)	34 (0·3%)	44 (0·3%)
22	44 (0·2%)	14 (0·1%)	30 (0·2%)
23	3 (0·0%)	3 (0·0%)	0 (0·0%)
24	4 (0·0%)	1 (0·0%)	3 (0·0%)

Supplementary Table S5. Number of postoperative surveillance interventions provided to high-risk patients

	**Day 0**	**Day 1**
No intervention	66 (2·6%)	123 (4·8%)
One intervention	244 (9·6%)	319 (12·5%)
Two interventions	345 (13·5%)	402 (15·8%)
Three interventions	603 (23·7%)	554 (21·7%)
Four interventions	879 (34·5%)	814 (31·9%)
Five interventions	411 (16·1%)	336 (13·2%)

Supplementary Table S6. Sensitivity analyses of the potential impact of unobserved outcomes on the primary and secondary effectiveness estimates, where individuals with unobserved outcomes were alternately assumed to be alive at discharge (censored at thirty days) or to have died in-hospital within 30 days

**Sensitivity analysis**	**Relative risk**	**2.5% confidence interval**	**97.5% confidence interval**	**p-value**
Primary outcome				
Unobserved outcomes assumed alive	0·96	0·69	1·32	0·79
Unobserved outcomes assumed dead	1·13	0·67	1·91	0·65
Secondary outcome				
Unobserved outcomes assumed alive	0·92	0·73	1·17	0·50
Unobserved outcomes assumed dead	1·07	0·82	1·38	0·63

Supplementary Table S7. Per protocol analyses of enhanced postoperative surveillance implementation fidelity to decrease in-hospital mortality.

	Fidelity definition 1:<br><br>(High-risk individuals exposed to the bedside guide plus at least 1 additional component of the intervention)	Fidelity definition 2:<br><br>(High-risk individuals exposed to at least any 2 components of the intervention)
Per protocol population 1: (Intervention hospitals where >= 80% of high-risk patients received the intervention with fidelity)	RR 1·20, 95% CI 0·76-1·88<br><br>p-value 0·44	RR 1·12, 95% CI 0·75-1·67<br><br>p-value 0·58
Per protocol population 2: (Top two tertiles of intervention hospitals ranked according to proportion of patients receiving the intervention with fidelity)	RR 1·07, 95% CI 0·75-1·53<br><br>p-value 0·69	RR 1·17, 95% CI: 0·82-1·67<br><br>p-value 0·38

RR relative risk, CI confidence interval

Supplementary Table S8. Per protocol analyses of enhanced postoperative surveillance implementation fidelity to decrease in-hospital severe complications and death

	Fidelity definition 1:<br><br>(High-risk individuals exposed to the bedside guide plus at least 1 additional component of the intervention)	Fidelity definition 2:<br><br>(High-risk individuals exposed to at least any 2 components of the intervention)
Per protocol population 1: (Intervention hospitals where >= 80% of high-risk patients received the intervention with fidelity)	RR 1·16, 95% CI 0·88-1·53)<br><br>p-value 0·30	RR 1·10, 95% CI 0·85-1·43<br><br>p-value 0·46
Per protocol population 2: (Top two tertiles of intervention hospitals ranked according to proportion of patients receiving the intervention with fidelity)	RR 1·07, 95% CI 0·84-1·36<br><br>p-value 0·58	RR 1·06, 95% CI 0·83-1·35<br><br>p-value 0·66

RR relative risk, CI confidence interval

Supplementary Table S9. Stratified models for primary effectiveness outcome associated with enhanced postoperative surveillance

Strata	Relative risk	2.5% confidence interval	97.5% confidence interval	p-value
Hospital level				
Primary	1·68	0·53	5·32	0·38
Secondary	0·98	0·52	1·84	0·96
Tertiary	0·86	0·59	1·26	0·44
Randomisation wave				
Wave 1	1·24	0·56	2·77	0·60
Wave 2	2·41	0·57	10·30	0·23
Wave 3	0·25	0·04	1·49	0·13
Wave 4	2·12	0·68	6·60	0·19
Wave 5	0·35	0·07	1·70	0·19
Wave 6	1·18	0·60	2·34	0·63
Wave 7	1·89	0·79	4·51	0·15
Wave 8	0·86	0·46	1·60	0·63
Wave 9	0·72	0·20	2·60	0·62
Wave 10	0·44	0·14	1·43	0·17
Wave 11	0·66	0·11	4·10	0·66
Country income category				
Low	1·04	0·69	1·58	0·84
Middle	0·56	0·23	1·39	0·21
High	0·91	0·50	1·66	0·76

Supplementary Table S10. Sensitivity analyses for primary effectiveness outcome associated with enhanced postoperative surveillance

Model	Strata	Relative risk	2.5% confidence interval	97.5% confidence interval	p-value
Per protocol definition 1 Fidelity definition 1					
Age					
	18-29	1·60	0·76	3·36	0·22
	30-69	1·10	0·70	1·72	0·69
	≥70	1·17	0·51	2·68	0·72
ASA category					
	ASA-all	1·16	0·76	1·75	0·49
	ASA 1	1·71	0·58	5·10	0·33
	ASA 2	1·85	0·86	4·02	0·12
	ASA 3	1·41	0·88	2·27	0·16
	ASA 4	0·47	0·19	1·14	0·10
	ASA 5	1·83	0·46	7·25	0·39
Grade of surgery					
	Severity-all	1·16	0·76	1·75	0·49
	Minor	2·48	0·47	13·09	0·28
	Intermediate	1·28	0·63	2·63	0·50
	Severe	0·99	0·63	1·56	0·97
Urgency of surgery					
	Urgency-all	1·16	0·76	1·75	0·49
	Elective	2·87	1·15	7·13	0·02
	Urgent	1·12	0·56	2·22	0·75
	Emergency	0·87	0·53	1·43	0·59
Surgical speciality					
	Gynaecology	3·33	0·80	13·85	0·10
	Obstetrics	0·69	0·23	2·02	0·49
	Orthopaedic	1·06	0·35	3·24	0·92
	Ear· nose and throat	3·41	0·63	18·37	0·15
	Plastics and Breast	2·02	0·21	19·77	0·55
	Neurosurgery	1·92	0·61	6·06	0·27
	Gastro-intestinal or Hepato-biliary	1·14	0·66	1·97	0·64
	Cardiothoracic or Vascular	0·26	0·03	2·07	0·20
	Other	0·47	0·06	3·76	0·48
Indication for surgery					
	Non-communicable disease	1·49	0·81	2·76	0·20
	Caesarean section	0·59	0·17	2·08	0·41
	Trauma	0·73	0·31	1·70	0·46
	Infection	1·00	0·53	1·89	1·00
Per protocol definition 1 Fidelity definition 2					
Age					

	18-29	1·35	0·67	2·74	0·40
	30-69	1·16	0·78	1·70	0·46
	≥70	1·03	0·49	2·19	0·94
ASA category					
	ASA-all	1·12	0·78	1·61	0·53
	ASA 1	1·64	0·60	4·47	0·33
	ASA 2	1·58	0·77	3·26	0·21
	ASA 3	1·38	0·89	2·15	0·15
	ASA 4	0·60	0·31	1·19	0·14
	ASA 5	1·20	0·25	5·76	0·82
Grade of surgery					
	Severity-all	1·12	0·78	1·61	0·53
	Minor	2·81	0·68	11·57	0·15
	Intermediate	1·21	0·64	2·29	0·55
	Severe	0·98	0·65	1·46	0·92
Urgency of surgery					
	Urgency-all	1·12	0·78	1·61	0·54
	Elective	2·44	1·07	5·59	0·03
	Urgent	1·12	0·61	2·07	0·71
	Emergency	0·88	0·57	1·38	0·59
Surgical speciality					
	Gynaecology	2·53	0·63	10·06	0·19
	Obstetrics	0·62	0·22	1·74	0·36
	Orthopaedic	0·98	0·36	2·68	0·97
	Ear· nose and throat	3·37	0·67	16·92	0·14
	Plastics and Breast	1·52	0·15	14·91	0·72
	Neurosurgery	1·85	0·67	5·08	0·23
	Gastro-intestinal or Hepato-biliary	1·08	0·66	1·77	0·76
	Cardiothoracic or Vascular	0·42	0·09	1·88	0·26
	Other	0·95	0·27	3·30	0·94
Indication for surgery					
	Non-communicable disease	1·40	0·82	2·41	0·22
	Caesarean section	0·46	0·13	1·63	0·23
	Trauma	0·82	0·38	1·78	0·62
	Infection	1·02	0·60	1·74	0·94
Per protocol definition 2 Fidelity definition 1					
Age	18-29	1·49	0·79	2·80	0·22
	30-69	1·05	0·74	1·49	0·78
	≥70	1·05	0·56	1·96	0·88
ASA category					
	ASA-all	1·07	0·78	1·48	0·66
	ASA 1	1·32	0·50	3·47	0·58

	ASA 2	1·61	0·88	2·95	0·12
	ASA 3	1·22	0·81	1·82	0·34
	ASA 4	0·68	0·38	1·21	0·19
	ASA 5	1·33	0·43	4·15	0·62
Grade of surgery					
	Severity-all	1·07	0·78	1·48	0·66
	Minor	1·44	0·38	5·47	0·59
	Intermediate	1·15	0·67	2·00	0·61
	Severe	1·03	0·72	1·48	0·87
Urgency of surgery					
	Urgency-all	1·07	0·78	1·48	0·66
	Elective	1·92	0·88	4·19	0·10
	Urgent	1·05	0·62	1·79	0·85
	Emergency	0·97	0·64	1·47	0·90
Surgical speciality					
	Gynaecology	2·10	0·52	8·49	0·30
	Obstetrics	0·53	0·18	1·55	0·25
	Orthopaedic	0·67	0·25	1·80	0·43
	Ear· nose and throat	2·58	0·58	11·50	0·22
	Plastics and Breast	1·54	0·26	9·15	0·63
	Urology	0·38	0·08	1·78	0·22
	Neurosurgery	1·30	0·48	3·51	0·61
	Gastro-intestinal or Hepato-biliary	1·16	0·76	1·76	0·49
	Cardiothoracic or Vascular	0·64	0·29	1·40	0·26
	Other	1·06	0·36	3·13	0·92
Indication for surgery					
	Non-communicable disease	1·40	0·87	2·27	0·17
	Caesarean section	0·40	0·11	1·47	0·17
	Trauma	0·65	0·33	1·28	0·21
	Infection	1·02	0·65	1·62	0·92
Per protocol definition 2 Fidelity definition 2					
Age					
	18-29	1·54	0·82	2·90	0·18
	30-69	1·19	0·85	1·68	0·31
	≥70	1·03	0·53	1·99	0·93
ASA category					
	ASA-all	1·17	0·85	1·61	0·35
	ASA 1	1·34	0·49	3·71	0·57
	ASA 2	1·63	0·87	3·06	0·13
	ASA 3	1·33	0·89	2·00	0·17
	ASA 4	0·91	0·53	1·54	0·71
	ASA 5	1·67	0·53	5·20	0·38
Grade of surgery					

	Severity-all	1·17	0·85	1·61	0·35
	Minor	1·81	0·48	6·84	0·38
	Intermediate	1·28	0·72	2·25	0·40
	Severe	1·09	0·76	1·57	0·65
Urgency of surgery					
	Urgency-all	1·17	0·85	1·61	0·35
	Elective	2·10	0·96	4·55	0·06
	Urgent	1·11	0·65	1·93	0·70
	Emergency	1·06	0·70	1·60	0·79
Surgical speciality					
	Gynaecology	2·48	0·64	9·54	0·19
	Obstetrics	0·68	0·25	1·83	0·45
	Orthopaedic	0·87	0·34	2·18	0·76
	Ear· nose and throat	2·49	0·56	11·16	0·23
	Plastics and Breast	1·84	0·31	10·80	0·50
	Urology	0·40	0·08	1·90	0·25
	Neurosurgery	1·50	0·57	3·97	0·41
	Gastro-intestinal or Hepato-biliary	1·21	0·79	1·86	0·39
	Cardiothoracic or Vascular	0·55	0·22	1·36	0·19
	Other	1·35	0·45	4·03	0·59
Indication for surgery					
	Non-communicable disease	1·41	0·86	2·31	0·17
	Caesarean section	0·43	0·12	1·55	0·20
	Trauma	0·94	0·51	1·77	0·86
	Infection	1·09	0·68	1·74	0·73

Per protocol definition 1; where the intervention hospital provided the intervention with fidelity to at least 80% of high-risk patients
Per protocol definition 2; hospitals in the top two tertiles of implementation fidelity
Fidelity definition 1; provision of at least the high-risk bedside guide plus one additional component of the intervention on days 0 and 1 postoperatively
Fidelity definition 2; provision of at least any two components of the intervention on days 0 and days 1 postoperatively, which did not necessarily have to include the high-risk beside guide as one of the components
ASA=American Society of Anesthesiologists

Supplementary Table S11. Sensitivity analyses for primary effectiveness outcome associated with enhanced postoperative surveillance for high-risk patients

Model stratification	Strata	Relative risk	2.5% confidence interval	97.5% confidence interval	p-value
Age					
	18-29	1·34	0·73	2·47	0·35
	30-69	1·14	0·82	1·56	0·44
	≥70	0·97	0·54	1·76	0·93
ASA category					
	ASA-all	1·09	0·81	1·47	0·59
	ASA 1	1·19	0·47	3·02	0·71
	ASA 2	1·59	0·91	2·78	0·10
	ASA 3	1·24	0·84	1·83	0·27
	ASA 4	0·69	0·41	1·15	0·16
	ASA 5	1·62	0·56	4·66	0·37
Grade of surgery					
	Severity-all	1·09	0·81	1·47	0·59
	Minor	1·40	0·40	4·90	0·60
	Intermediate	1·25	0·75	2·09	0·39
	Severe	1·04	0·74	1·46	0·83
Urgency of surgery					
	Urgency-all	1·09	0·81	1·47	0·59
	Elective	1·74	0·83	3·65	0·15
	Urgent	0·98	0·59	1·62	0·94
	Emergency	1·08	0·74	1·58	0·69
Surgical speciality					
	Gynaecology	1·94	0·50	7·59	0·34
	Obstetrics	0·51	0·18	1·40	0·19
	Orthopaedic	0·62	0·25	1·58	0·32
	Ear, nose and throat	2·38	0·54	10·54	0·25
	Plastics and Breast	1·21	0·20	7·26	0·83
	Urology	0·32	0·07	1·50	0·15
	Neurosurgery	1·33	0·54	3·28	0·53
	Gastro-intestinal or Hepato-biliary	1·25	0·85	1·82	0·26
	Cardiothoracic or Vascular	0·57	0·26	1·26	0·17
	Other	0·99	0·36	2·69	0·98
Indication for surgery					
	Non-communicable disease	1·39	0·88	2·18	0·16
	Caesarean section	0·33	0·09	1·20	0·09
	Trauma	0·86	0·48	1·56	0·63
	Infection	1·00	0·65	1·54	0·99

ASA=American Society of Anesthesiologists

Supplementary Figure 1. The African Surgical OutcomeS-2 (ASOS-2) trial results

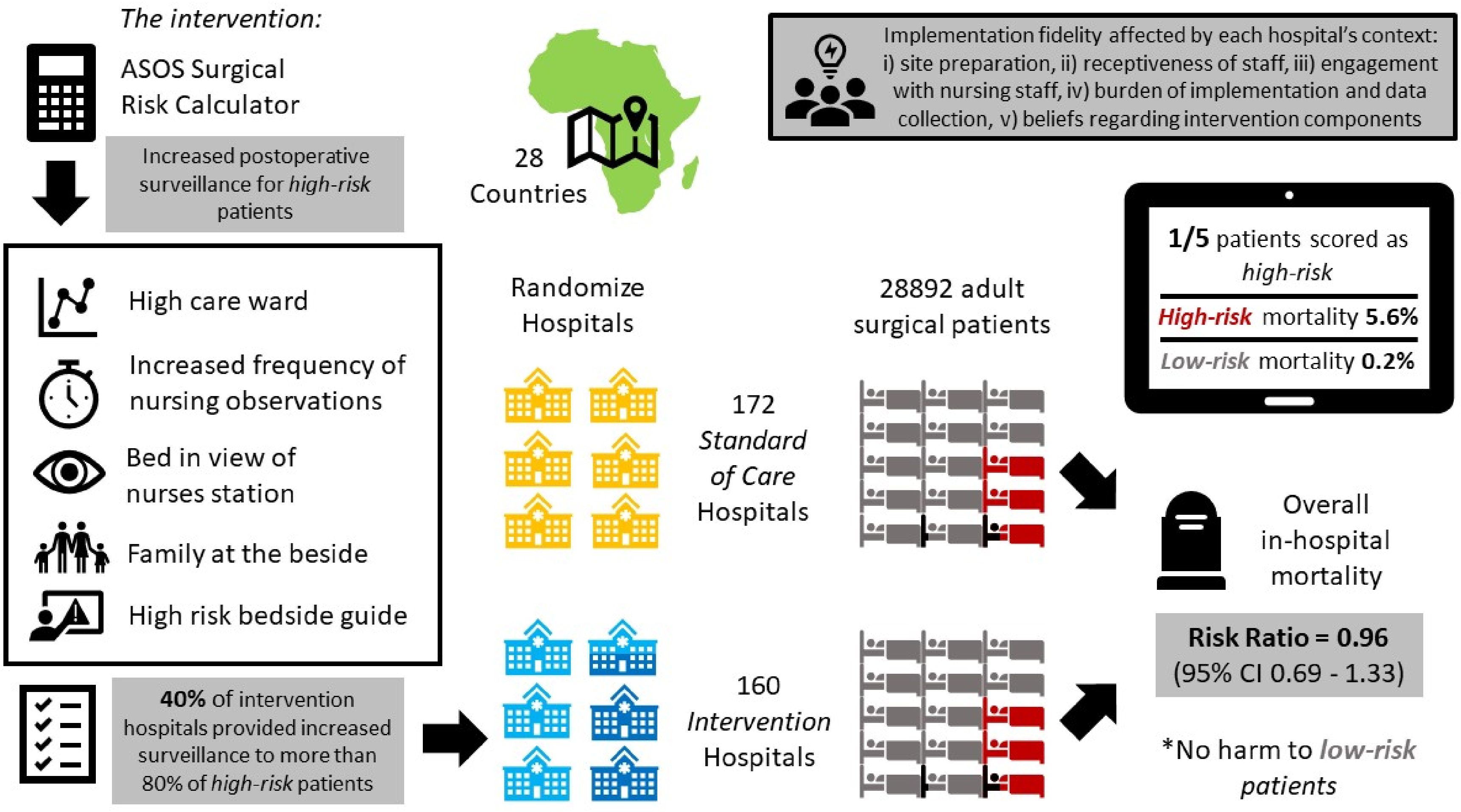

Supplement: Supplementary appendix 4 [file mmc4.pdf]
